# Supplementary material for: Amount and type of physical activity and sports from one year forward after hip or knee arthroplasty—A systematic review
Source: PLoS One. 2021 Dec 28;16(12):e0261784. doi: 10.1371/journal.pone.0261784 (PMC8714096; doi:10.1371/journal.pone.0261784)
Supplement: S1 Appendix — (PDF) [file pone.0261784.s001.pdf]

Appendix 1: Search strategy

**Table 1.** Search strings for Pubmed, EMBASE, PsycInfo, and Cinahl.	
**Database**	**Search string**
**Pubmed**	("Arthroplasty, Replacement, Knee"[Mesh] OR "Arthroplasty, Replacement, Hip"[Mesh] OR hip arthroplast*[ti] OR hip replacement*[ti] OR hip reconstruction*[ti] OR knee arthroplast*[ti] OR knee replacement*[ti] OR knee reconstruction*[ti] OR total joint arthroplast*[ti] OR total joint replacement*[ti] OR total joint reconstruction*[ti] OR lower limb arthroplast*[ti] OR lower limb reconstruction*[ti] OR lower limb replacement*[ti] OR tha[ti] OR thr[ti] OR tka[ti] OR tkr[ti] OR tjr[ti] OR tja[ti]) AND ("Sports"[Mesh] OR sport*[ti] OR activ*[ti] OR inactiv*[ti] OR "Exercise" OR exercise*[ti] OR "Motor Activity"[Mesh:NoExp] OR "Life Style"[Mesh] OR life style*[ti] OR physical fitness[ti] OR lifestyle*[ti] OR pa[ti] OR leisure[ti] OR sedentar*[ti] OR behav*[ti])
**EMBASE**	('knee replacement'/exp OR 'hip replacement'/exp OR 'hip arthroplast*':ti OR 'hip replacement*':ti OR 'hip reconstruction*':ti OR 'knee arthroplast*':ti OR 'knee replacement*':ti OR 'knee reconstruction':ti OR 'total joint arthroplast*':ti OR 'total joint replacement':ti OR 'total joint reconstruction*':ti OR 'lower limb arthroplast*':ti OR 'lower limb replacement*':ti OR 'lower limb reconstruction*':ti OR 'tha':ti OR 'thr':ti OR 'tka':ti OR 'tkr':ti OR 'tjr':ti OR 'tja':ti)<br><br>AND ('sport'/exp OR 'sport*':ti OR ‘activ*’:ti OR ‘inactiv*’:ti OR 'exercise'/exp OR 'exercise*':ti OR 'motor control'/mj OR 'lifestyle'/exp OR 'life style*':ti OR ‘lifestyle*’:ti OR ‘physical fitness’:ti OR ‘pa’:ti OR ‘leisure’:ti OR ‘sedentar*’:ti OR ‘behav*’:ti)
**PsycInfo**	(TI (“hip arthroplast*” OR “hip replacement*” OR “hip reconstruction*” OR “knee arthroplast*” OR “knee replacement*” OR “knee reconstruction*”) OR AB (“hip arthroplast*” OR “hip replacement*” OR “hip reconstruction*” OR “knee arthroplast*” OR “knee replacement*” OR “knee reconstruction*”) OR TI (“total joint arthroplast*” OR “total joint replacement*” OR “total joint reconstruction*” OR “lower limb arthroplast*” OR “lower limb reconstruction*” OR “lower limb replacement”* OR tha OR thr OR tka OR tkr OR tjr OR tja)) AND ((DE "Sports") OR (DE "Exercise") OR (DE "Life Style") OR (DE "Physical Activity") OR TI (sport* OR activ* OR inactiv* OR exercise* OR “life style*” OR “physical fitness” OR lifestyle* OR pa OR leisure OR sedentar* OR behav*))
**Cinahl**	((MH "Arthroplasty, Replacement, Knee+") OR (MH "Arthroplasty, Replacement, Hip") OR TI (“hip arthroplast*” OR “hip replacement*” OR “hip reconstruction*” OR “knee arthroplast*” OR “knee replacement*” OR “knee reconstruction*”) OR AB (“hip arthroplast*” OR “hip replacement*” OR “hip reconstruction*” OR “knee arthroplast*” OR “knee replacement*” OR “knee reconstruction*”) OR TI (“total joint arthroplast*” OR “total joint replacement*” OR “total joint reconstruction*” OR “lower limb arthroplast*” OR “lower limb reconstruction*” OR “lower limb replacement”* OR tha OR thr OR tka OR tkr OR tjr OR tja)) AND ((MH "Sports+") OR (MH "Exercise+") OR (MH "Life Style+") OR (MM "Motor Activity") OR TI (sport* OR activ* OR inactiv* OR exercise* OR “life style*” OR “physical fitness” OR lifestyle* OR pa OR leisure OR sedentar* OR behav*))
