# Supplementary material for: Amount and type of physical activity and sports from one year forward after hip or knee arthroplasty—A systematic review
Source: PLoS One. 2021 Dec 28;16(12):e0261784. doi: 10.1371/journal.pone.0261784 (PMC8714096; doi:10.1371/journal.pone.0261784)
Supplement: S2 Appendix — (PDF) [file pone.0261784.s002.pdf]

Appendix 2. Methodological quality tool.

Selection	Was the sample selected appropriate for the study group, i.e. was it chosen from a representative group in a random/consecutive way?
Inclusion/exclusion criteria	Were the inclusion/exclusion criteria described?
Nonresponse bias	Did the group finally selected represent a proper proportion of the sample? A cut-off for response rate and drop-outs of 80% and 20% was chosen. If less than 80% responded or more than 20% dropped out of the study, this was considered as possible response bias.
Non-respondents analysis	Was a non-respondents analysis performed?
Objective design	Did the design of the study use objective measurements to assess physical activity, like pedometers and accelerometers?
Study size	Was the size of the study large enough to provide a proper estimate of the activity of the sample? Cut-off was set at 40 patients for objective studies and 100 for subjective studies.
Outcome measures	Were the outcome measures appropriate and validated?
Report	Were the outcomes clearly reported?
