# Supplementary material for: Amount and type of physical activity and sports from one year forward after hip or knee arthroplasty—A systematic review
Source: PLoS One. 2021 Dec 28;16(12):e0261784. doi: 10.1371/journal.pone.0261784 (PMC8714096; doi:10.1371/journal.pone.0261784)
Supplement: S3 Appendix — (PDF) [file pone.0261784.s003.pdf]

Appendix 3. Quality assessment

Article	Selection random or consecutive	Inclusion/ exclusion criteria	Response rate ≤80% / Drop-outs ≤20%	Non-respondents analysis	Objective study design	Study size ≥100 subjective/ ≥40 objective	Outcome measures	Clearly reported	Total score
Abe et al. 2014	?	+	?	-	-	+	+	+	4
Alvarez et al. 2015	+	+	?	-	+	+	+	-	5
Amstutz et al. 2019	+	+	-	-	-	+	+	+	5
Banerjee et al. 2010	+	+	+	+	-	+	+	-	6
Batailler et al. 2019	+	+	+	+	-	-	+	+	6
Bercovy et al. 2015	+	+	+	-	-	+	+	+	6
Bin Sheeha et al. 2020	+	+	-	+	+	-	+	+	6
Blikman et al. 2013	+	+	?	-	+	+	+	+	6
Bonnin et al. 2010	+	+	-	-	-	+	+	+	5
Bonnin et al. 2018	+	+	+	-	-	+	+	+	6
Bonnin et al. 2020	?	+	+	+ (NA)	-	+	+	+	6
Brandes et al. 2011	-	+	?	-	+	+	+	+	6
Breuer et al. 2020	+	+	+	-	-	-	?	+	4
Canetti et al. 2018	+	+	+	+ (NA)	-	-	+	+	6
Casazza et al. 2020	?	+	?	-	+	-	+	+	4
Chang et al. 2014 & 2015	?	+	-	-	-	+	+	+	4
Clement et al. 2019	?	+	+	-	+	+	+	+	6
Cowie et al. 2013	+	+	?	-	-	+	+	+	5
Crawford et al. 2020	+	+	+	-	-	+	+	+	6
Daugaard et al. 2018	-	+	?	-	+	+	+	+	5
Delfin et al. 2017	+	+	-	-	-	-	+	+	4
Donner et al. 2019	+	+	+	+ (NA)	-	-	+	+	6
Dubin et al. 2020	+	+	+	-	-	+	+	+	6
Eckhard et al. 2020	?	+	-	+	-	+	+	+	5
Elman et al. 2014	+	-	-	+	-	-	+	+	4
Felts et al. 2010	+	+	+	+ (NA)	-	-	+	+	6
Fisher et al. 2011	+	-	+	+ (NA)	-	+	+	-	5
Foucher et al. 2018	-	+	-	+	-	-	+	+	4
Fouilleron et al. 2012	+	+	+	+ (NA)	-	-	+	+	6
Fujita et al. 2013	+	+	-	+	+	-	+	+	6
Gerhardt et al. 2017	+	+	+	+	-	-	+	+	6
Ghomrawi et al. 2017	?	-	-	+	-	+	+	+	4
Girard et al. 2013	+	+	+	+ (NA)	-	-	+	+	6
Groen et al. 2012	+	+	-	+	-	+	+	+	6
Hara et al. 2018	+	+	-	-	-	+	+	-	4
Hayashi et al. 2012	?	-	?	-	-	-	+	+	2
Hayashi et al. 2016	?	+	?	-	-	-	+	+	3
Hayes et al. 2011	?	+	?	-	+	-	-	-	2
Heiberg et al. 2016	+	+	-	-	-	-	+	+	4
Hepperger et al. 2018	+	+	+	-	-	+	+	+	6
Hjorth et al. 2018	?	+	?	-	+	+	+	+	5
Ho et al. 2016	+	+	-	-	-	-	+	+	4

Article	Selection random or consecutive	Inclusion/ exclusion criteria	Response rate ≤80% / Drop-outs ≤20%	Non-respondents analysis	Objective study design	Study size ≥100 subjective/ ≥40 objective	Outcome measures	Clearly reported	Total score
Hodges et al. 2018	?	+	-	-	-	+	+	+	4
Hylkema et al. 2020	?	+	?	-	+	+	+	+	5
Ibrahim et al. 2019	?	+	?	-	-	-	+	-	2
Innmann et al. 2016	+	+	+	-	-	-	+	+	5
Issa et al. 2015	+	-	+	-	-	+	+	+	5
Jacquet et al. 2020	+	+	+	-	-	-	+	+	5
Jahnke et al. 2015	+	+	+	+	-	+	+	+	7
Jassim et al. 2019	?	+	+	-	-	-	+	+	4
Jeldi et al. 2017	+	+	-	-	+	-	+	+	5
Jelsma et al. 2019	?	+	+	-	+	+	+	+	6
Jelsma et al. 2020a & 2020b	+	+	+	-	+	-	+	+	6
Jonas et al. 2019	+	-	-	+	-	-	+	-	3
Jones et al. 2012	+	+	+	-	-	-	+	+	5
Karampinas et al. 2017	-	+	?	-	-	-	+	+	3
Keeney et al. 2014	+	+	-	-	-	+	+	+	5
Keeney et al. 2015	+	+	-	-	-	+	+	+	5
Kersten et al. 2012	+	+	-	+	-	+	+	+	6
Kim et al. 2016	+	+	+	-	-	+	+	+	6
Kim et al. 2019	?	+	+	+	-	-	+	-	4
Kiran et al. 2019	+	+	+	+ (NA)	-	-	+	+	6
Kleeblad et al. 2020	+	+	-	+	-	+	+	+	6
Krantz et al. 2012	+	+	+	+ (NA)	-	-	+	-	5
Krych et al. 2017	+	+	-	+	-	+	+	+	6
Kuhn et al. 2013	?	+	-	-	+	-	+	+	4
Le Duff & Amstutz, 2011	+	-	-	-	-	+	-	+	3
Lefevre et al. 2013	-	+	-	-	-	-	+	+	3
Lo Presti et al. 2019	+	+	+	-	-	-	+	+	5
Long et al. (2014)	+	+	-	+	-	-	+	+	5
Lubbeke et al. 2014	+	+	-	+	-	+	+	+	6
Lutzner et al. 2014	+	+	-	+	+	+	+	+	7
Lutzner et al. 2016	?	-	-	-	+	+	+	+	4
Madrid et al. 2019	+	+	-	-	-	+	+	-	4
Majewski et al. 2014	+	+	-	+	-	-	+	+	5
Malcolm et al. 2014	+	+	-	+	-	+	+	+	6
Martin et al. 2018	+	+	+	-	-	-	+	+	5
Matsunaga-Myoji et al. 2020a	+	+	-	+	+	+	+	+	7
Matsunaga-Myoji et al. 2020b	+	+	-	+	+	+	+	+	7
Mayr et al. 2015	+	+	+	+	-	-	+	+	6
Meding et al. 2012	+	+	-	+	-	-	+	+	5
Mesko & Heath 2011	+	-	-	-	-	-	-	-	1
Naylor et al. 2019	-	+	-	+	-	+	+	+	5
Ninomiya et al. 2018	+	+	-	-	-	-	+	+	4
Ollivier et al. 2014	+	+	-	-	-	+	-	+	4
Ortmaier et al. 2017	+	+	+	-	-	+	+	+	6

Article	Selection random or consecutive	Inclusion/ exclusion criteria	Response rate ≤80% / Drop-outs ≤20%	Non-respondents analysis	Objective study design	Study size ≥100 subjective/ ≥40 objective	Outcome measures	Clearly reported	Total score
Panzram et al. 2018	+	+	+	+ (NA)	-	-	+	+	6
Panzram et al. 2020	+	-	+	-	-	+	+	+	5
Paxton et al. 2016	+	+	-	+	-	+	+	+	6
Payo-Ollero et al. 2020	+	+	?	-	-	-	+	+	4
Pietschmann et al. 2013	+	+	-	-	-	+	+	-	4
Pioger et al. 2020	+	+	?	-	-	+	+	+	5
Pisanu et al. 2020	+	+	+	-	-	+	+	+	6
Plassard et al. 2020	+	+	+	-	-	+	?	+	5
Plate et al. 2013	+	+	?	-	-	-	+	+	4
Ponzio et al 2018	+	+	-	-	-	+	+	+	5
Poortinga et al. 2014	+	+	+	+	-	+	+	+	7
Postler et al. 2017	+	+	-	-	-	+	+	+	5
Pritchett 2018	?	+	?	-	-	+	+	+	4
Ristolainen et al. 2019	+	+	-	-	-	-	+	+	4
Robertson et al. 2016	-	-	-	-	+	-	+	+	3
Rolving et al. 2013	+	+	+	+	-	-	+	+	6
Rozenlund et al. 2017	+	+	-	+	-	-	+	-	4
Rueckl et al. 2020	+	+	-	-	-	-	+	+	4
Sandberg et al. 2019	?	+	+	+	-	+	+	+	6
Sandiford et al. 2015	+	-	+	+ (NA)	-	-	+	-	4
Schmidutz et al. 2012	+	+	+	+ (NA)	-	-	+	+	6
Schneider et al. 2020	?	+	-	-	-	-	+	+	3
Scott et al. 2017	+	+	+	+ (NA)	-	+	+	+	7
Smith et al. 2017 (T)	+	+	-	+	-	+	+	+	6
Smith et al. 2018 Does PA change	?	+	?	-	-	+	+	+	4
Stambourgh et al. 2014	+	+	+	+ (NA)	-	-	+	+	6
Takenaga et al 2013	+	+	-	-	+	+	+	+	6
Takeuchi et al. 2020	?	+	?	-	-	+	+	+	4
Vielgut et al. 2016	?	-	+	-	-	+	-	+	3
Von Rottkay et al. 2018	?	+	?	-	+	+	+	+	5
Wagenmakers et al. 2011	+	+	-	-	-	+	+	+	5
Walker et al. 2015a (return to sports)	+	+	+	+ (NA)	-	-	+	+	6
Walker et al. 2015b (sports, PA)	+	+	+	+ (NA)	-	-	+	+	6
Webber et al. 2017	-	+	-	+	+	-	+	+	5
Wimmer et al. 2015	?	+	?	-	+	-	-	+	3
Wollmerstedt et al. 2010	-	+	?	-	+	+	+	+	5
Zimmerer et al. 2021	+	+	-	-	-	-	+	-	3
