# Supplementary material for: Amount and type of physical activity and sports from one year forward after hip or knee arthroplasty—A systematic review
Source: PLoS One. 2021 Dec 28;16(12):e0261784. doi: 10.1371/journal.pone.0261784 (PMC8714096; doi:10.1371/journal.pone.0261784)
Supplement: S4 Appendix — (PDF) [file pone.0261784.s004.pdf]

Appendix 4. Overview of characteristics and results of studies reporting on amount and type of physical activity.

Study	Country	Arthroplasty	Study design	Sample size and Characteristics*	Data collection period (FU & time postop)	Type of sport	Measurement method	Outcome variables of interest	Outcomes
**Abe et al. 2014**	Japan	THA<br>HRA	Case-control study	Patients: 608<br>Age: 62 (36-98)<br>14% male<br>BMI: 23 (14-34)<br>32% bilateral<br>Surgery: 90% THA, 10% HRA	4.8 (2.3-7.8) years	Activity<br>Jogging	UCLA<br>Questionnaire on jogging routines	Activity (1-10)<br>Participation (%)<br>Frequency (sessions/wk)<br>Distance (km/session)<br>Duration (min/session)<br>Velocity (km/hour)	Joggers: 10±0; non-joggers: 6.6±2.4<br>a) Preop: 5.4%; postop: 3.8%<br>4 (1-7)<br>3.6 (0.5-15)<br>29 (5-90)<br>7.7 (3-18)
**Alvarez et al. 2015**	Spain	THA	Cross-sectional study	Patients: 47<br>Age: 63.8±11.7<br>55% male<br>BMI: 29.6±5.6<br>19% bilateral	>1 year	Activity	UCLA<br>IPAQ<br>Accelerometer	Activity (1-10)<br>IPAQ score<br>PA (min/wk)	5.5±1.3<br>1.8±0.6<br>148.9±69.8
**Amstutz et al. 2019**	United States	MoM HRA	Survey	Patients: 661<br>Age: 51.9 (14-78)<br>70% male<br>BMI: 26.5 (16.7-46.5)	10.1 (1-16) years	Sports participation	Questionnaire	Sports participation<br>Type<br><br>Activity quantity (frequency*duration)	100%<br>Walking/hiking, cycling, gym workouts most-performed<br>12.4 (0-48)<br>- Frequency (1: 1-4x/mo; 2: 5-8x/mo; 3: 9-12x/mo; 4: >12x/mo)<br>- Duration (1: 0-30min; 2: 30-60min; 3: 60-120min; 4: >120min)
**Banerjee et al. 2010**	Germany	HRA	Case series	Patients: 138<br>Age: 52.6 (38-71)<br>Male hips: 59%<br>10% bilateral	23.5 (12-42) months	Activity<br>Sports participation	Grimby scale<br>Questionnaire	Activity (1-6)<br>Sports participation rate<br># sport activities	Preop: 3.3, postop: 4.6, p< .001<br>Preop: 98%, postop: 97%<br>Preop 3.6 (0-10), postop: 3.2 (0-8)
								Duration of activities (<2, 2-4, >4 hours)<br>Impact of activities (low=1, medium=2, high=3)<br>Sports type	65% same, 25% increased, 10% decreased duration<br>Preop: 2.1, postop: 1.5, p<0.001<br>Impact: 58% performed sports in same impact category, 5% in higher category, 37% in lower category<br>Several high-impact activities showed decreased participation rate, several low-impact activities had higher participation rates<br>Longer participation in less vigorous activities
**Batailler et al. 2019**	France	THA	Comparative retrospective study	Patients: 32<br>Age: 60.7±9.6<br>66% male<br>BMI: 26±4<br>100% bilateral	20.1±11.6 months	Activity<br>Sports participation	UCLA<br>Questionnaire	Activity (1-10)<br>RTS (%)<br>Time to RTS, months<br>RTS intensity	Preop: 6.1±1.7; postop: 5.6±8.1<br>87.5% returned to sports<br>4.2±2.7<br>68% same intensity
				Patients: 64<br>Age: 61.8±10<br>66% male<br>BMI: 25.9±4<br>0% bilateral	28.9±15 months	Activity<br>Sports participation	UCLA<br>Questionnaire	Activity (1-10)<br>RTS (%)<br>Time to RTS, months<br>RTS intensity	Preop: 5.7±1.9; postop: 4.9±2.2<br>57.8% returned to sports<br>5.4±5.1 months<br>56.5% same intensity
**Bercovy et al. 2015**	France	TKA	Therapeutic study	Patients: 403<br>Age: 70.36 (40.11-91.2)<br>34% male<br>BMI: 29.6 (19.8-47.6)<br>23% bilateral	7.5 (5-13) years	Activity<br>Sports activity	UCLA<br>Questionnaire	Activity (1-10)<br><br><br><br># sports, n	Pre-symptom: 7.4±1.4<br>Preop: 3.8<br>Postop: 7.3±1.7 (1-10)<br>80% ≥7; 27% ≥8<br>72% ≥1 sports

Study	Country	Arthroplasty	Study design	Sample size and Characteristics*	Data collection period (FU & time postop)	Type of sport	Measurement method	Outcome variables of interest	Outcomes
**Bin Sheeha et al. 2020**	Saudi Arabia / United Kingdom	TKA	Prospective cohort study	Patients: 33<br><br>Male: 6<br>Age: 76±7 (63-85)<br>BMI: 32.38±2.01<br><br>Female: 27<br>Age: 59±6 (49-76)<br>BMI: 37.21±7.65	12 months	Activity	Accelerometer	Stepping time (hour)<br>Steps (steps/day)<br>Time sedentary (hours)<br>Time standing (hours)<br>Upright time (hours)<br>Cadence:<br><60 steps/min (min/wk)<br>Slow to medium (60-100 steps/min) (min/wk)<br>Moderate/vigorous (>100 steps/min) (min/wk)<br>Meeting guidelines 150 min MVPA:<br>All stepping events, (%)<br>Walk events >10min (%)<br>Minimal 5 min bouts (%)<br>Minimal 1 min bouts (%)	Preop: 0.98±0.43; postop: 1.36±0.38<br>Preop: 4240±2268; postop: 6174±2287<br>Preop: 19.48±1.51; postop: 19.08±1.54<br>Preop: 3.47±1.27; postop: 3.54±0.97<br>Preop: 4.48±1.45; postop: 4.88±1.47<br><br>Preop: 369±189; postop: 593±188<br>Preop: 232±148; postop: 457±179<br><br>Preop: 6.6; Postop: 41.7<br><br>Postop: 24%<br>Postop: 6%<br>Postop: 9%<br>Postop: 12%
**Blikman et al. 2013**	The Netherlands	THA (43%)<br>TKA (45%)<br>THA & TKA (11%)	Psychometric assessment	Patients: 44<br>Age: 72±9<br>23% male<br>BMI: 28±5.4	>1 year	Activity	IPAQ short-form<br><br><br><br><br><br>IPAQ long-form<br><br><br><br><br><br>Accelerometer	Total activity (MET min/wk)<br>Total time (min/wk)<br>MPA (min/wk)<br>VPA (min/wk)<br>Sitting time (min/wk)<br>Total activity (MET min/wk)<br>Total time (min/wk)<br>MPA (min/wk)<br>VPA (min/wk)<br>Sitting time (min/wk)<br>Mean counts/min<br>Total activity (min/wk)<br>MPA (min/wk)<br>VPA (min/wk)<br>Sitting time (min/wk)	3370±3762<br>868±969<br>822±945<br>46±129<br>3123±1214<br>4498±3840<br>1108±922<br>1087±909<br>20±59<br>3255±1118<br>210±142<br>99±156<br>92±127<br>7±35<br>1252±314
**Bonnin et al. 2010**	France	TKA	Case series	Patients: 347<br>Age at FU: 74.8 (28-94)<br>35% male<br>BMI: 27.9±4.6<br>0% bilateral	44 (13-71) months	Sports participation	Questionnaire	Sports activity (%)<br><br><br>Sports impact (low, medium, high) in patients aged <75 (n=141)<br>Sport type	41.5% increased activity level, 26.8% decreased<br>56% participated regularly in light, 66% in intermediate, 10% in strenuous sport<br>You do not list this<br><br>Cycling, hiking, swimming most performed
**Bonnin et al. 2018**	France	CoC THA	Case-control study	Patients: 1042<br>Age: 60.6±8.8<br>57% male<br>BMI: 26.6±4.6<br>16% bilateral	44.4±14.0 months	Sports participation	Questionnaire	Sports participation (%)<br>Sports impact: light, moderate, strenuous (%)<br>Discomfort (none, moderate, severe)<br>Sport level<br>Sport type	91%<br>51% participated in ≥1 light sport, 71% in moderate, 20% in strenuous sport.<br>1-5% in light sport, 5-8% in moderate sport, 8-18% in strenuous sport reported severe discomfort during activity<br>2.6% played at competitive level<br>Hiking, cycling, swimming most performed

Study	Country	Arthroplasty	Study design	Sample size and Characteristics*	Data collection period (FU & time postop)	Type of sport	Measurement method	Outcome variables of interest	Outcomes
**Bonnin et al. 2020**	France	THA	Comparative study	Patients: 259 with posterolateral approach<br>Age: 61±7.8 (39-78)<br>63% male<br>BMI: 27±4.2 (17-41)	44±14.1 (24-74) months	Sports participation	Questionnaire	Participation (%)<br>Sport impact: light, moderate, strenuous (%)<br>Sport type	81% participated regularly/frequently<br>49% participated in ≥1 light sport, 69% in moderate sport and 19% in strenuous sport.<br>Hiking most-performed (41%), followed by cycling
				Patients: 259 with anterolateral approach<br>Age: 62±8.1 (21-76)<br>59% male<br>BMI: 27±4.6 (18-45)	43±14.5 (24-79) months	Sports participation	Questionnaire	Participation (%)<br>Sport impact: light, moderate, strenuous (%)<br>Sport type	81% participated regularly/frequently<br>49% participated in ≥1 light sport, 71% in moderate, 24% in strenuous sport.<br>Hiking most-performed (39%), followed by cycling
**Brandes et al. 2011**	Germany	TKA	Longitudinal follow-up study	Patients: 53<br>Age: 65.8±5.8<br>34% male<br>BMI: 30.7±4.1<br>0% bilateral	1 year	Activity	Activities of daily living monitor<br><br>Step monitor	Time walking %, n=32<br>Gait cycles/day, n=44<br><br>Gait intensity	Preop: 8.4±3.6, postop: 12.0±4.7<br>Preop: 4993±3170 gait cycles/day<br>Postop: 5932±2111 gait cycles/day<br>Walking happened at a higher intensity postop
**Breuer et al. 2020**	Austria	Short-stem THA	Prospective cohort study	Patients: 55<br>Age 61±10<br>53% male<br>BMI: 27.9±4.9	38±4.6 months	Activity<br>Sports participation	UCLA	Activity (1-10)<br>Sports participation (%)<br><br>Number of sports (n)<br>Sports impact (low, high, mixed), %	Preop: 4.5±1.8; postop: 6.9±1.9<br>98% after mean of 13±8 weeks. 89% returned, 9% started sports activity<br>Preop: 1.89±1.1; postop: 1.7±1.1<br>Preop: 28%, 43%, 17%; postop: 47%, 34%, 11%
**Canetti et al. 2018**	France	UKA	Comparative retrospective study	Patients: n=11, UCLA ≥5<br>Age: 66.5±6.8<br>BMI: 24.2±4.3<br>18% male<br>Surgery: Robotic UKA	34.4±10.5 months	Activity<br>Sports participation	UCLA<br>Face-to-face questioning	Activity (1-10)<br>RTS, %<br>Time to RTS, months<br>Number of sports<br>Intensity level<br>Sport type	6.4±1.6<br>100%<br>4.2±1.8 months<br>2.4 different sports<br>91% no change<br>Hiking, cycling and fitness most performed
				Patients: n=17, UCLA ≥5<br>Age: 59.5±9.9<br>BMI: 26.3±3.8<br>28% male<br>Surgery: normal UKA	39.3±15.5 months	Activity<br>Sports participation	UCLA<br>Face-to-face questioning	Activity (1-10)<br>RTS, %<br>Time to RTS, months<br>Number of sports<br>Intensity level<br>Sport type	5.8±0.9<br>94%<br>10.5±6.7 months<br>1.9 different sports<br>82.4% no change<br>Hiking, cycling, fitness most-performed
**Casazza et al. 2020**	United States	TKA	Prospective study	Patients: 7<br>Age: 55.6, SEM 3.5<br>39% male<br>BMI: 32.8, SEM 1.5	12 months	Activity	Accelerometer	Steps (steps/day)<br><br>Energy expenditure (kcal/day)	Preop: 5858 SEM 671; postop: 5981 SEM 751<br>Preop: 2377 SEM 229; postop: 2297 SEM 133
**Chang et al. 2014 & 2015**	South-Korea	TKA	Case series	Patients: 369<br>Age: 68.8 (50-83)<br>8% male<br>BMI: 27.4 (19.3-39.1)<br>68% bilateral	2 (1-3) years	Activity<br>Sports participation	UCLA<br>Questionnaire	Activity (1-10)<br>Sports participation (%)<br><br>Number of activities (n)<br>Sport impact<br><br>Sport type	4.8±1.4<br>24% no regular sport activities, UCLA 4.0±1.9<br>76% no regular sport activities, UCLA 4.6±1.7<br>1.7 activities<br>Decrease in high-impact sports, increase in moderate-impact sports<br>Walking, swimming, cycling most-performed

Study	Country	Arthroplasty	Study design	Sample size and Characteristics*	Data collection period (FU & time postop)	Type of sport	Measurement method	Outcome variables of interest	Outcomes
**Clement et al. 2019**	United Kingdom	THA	Comparative study	Patients: 200<br>Age: 69.9±9.2 (42-92)<br>43% male	12 months, 24 months	Activity	LEAS<br>Accelerometer	Activity (1-18)<br>Energy expenditure (MET/day)<br>Time spent sitting/lying (hrs/day)<br>Time spent standing (hrs/day)<br>Time spent walking (hrs/day)<br>Step count (steps/day)	*Patients aged <65*<br>Preop: 8.7±2.4; 12mo: 12.5±2.8; 24 mo: 12.0±3.1<br>32.8±1.3; 12 mo: 31.1±1.4; 24 mo: 30.4±1.2<br>Preop: 18.9±1.8; 12 mo: 18.3±2.2; 24 mo: 18.6±2.0<br>Preop: 3.9±1.5; 12 mo: 3.4±1.6; 24 mo: 3.0±1.3<br>Preop: 1.2±0.6; 12 mo: 1.2±0.5; 24 mo: 1.0±0.6<br>Preop: 5583±3211; 12 mo: 5370±2524; 24 mo: 4269±2905
								Activity (1-18)<br>Energy expenditure (MET/day)<br>Time spent sitting/lying (hrs/day)<br>Time spent standing (hrs/day)<br>Time spent walking (hrs/day)<br>Step count (steps/day)	*Patients aged 65-74*<br>Preop: 7.9±2.6; 12 mo: 11.5±2.9; 24 mo: 11.6±3.2<br>Preop: 32.3±1.4; 12 mo: 32.2±1.7; 24 mo: 31.1±1.5<br>Preop: 18.4±2.7; 12 mo: 17.6±2.9; 24 mo: 17.6±2.7<br>Preop: 4.5±2.3; 12 mo: 4.4±2.0; 24 mo: 4.3±2.2<br>Preop: 1.0±0.7; 12 mo: 1.3±0.7; 24 mo: 1.2±0.6<br>Preop: 4501±3273; 12 mo: 6071±3256; 24 mo: 5760±3199
								Activity (1-18)<br>Energy expenditure (MET/day)<br>Time spent sitting/lying (hrs/day)<br>Time spent standing (hrs/day)<br>Time spent walking (hrs/day)<br>Step count (steps/day)	*Patients aged ≥75*<br>Preop: 7.5±2.2; 12 mo: 8.7±2.9; 24 mo: 9.0±3.0<br>Preop: 31.7±0.9; 12 mo: 31.4±1.4; 24 mo: 29.9±1.5<br>Preop: 19.3±3.1; 12 mo: 18.5±1.3; 24 mo: 18.1±1.3<br>Preop: 4.0±3.1; 12 mo: 3.8±1.0; 24 mo: 3.3±0.8<br>Preop: 0.6±0.4; 12 mo: 0.8±0.4; 24 mo: 0.9±0.4<br>Preop: 2740±1977; 12 mo: 4344±2195; 24 mo: 3915±2054
**Cowie et al. 2013**	United Kingdom	THA (90%)<br>HRA (10%)	Case series	Patients: 239<br>Age: 55.2±7.2<br>35.2% male<br>BMI: 28.2±4.9<br>19% bilateral	3.1±0.97 years	Activity<br><br><br>Sports participation	Grimby scale<br><br><br>Questionnaire	Activity (1-6)<br><br><br>RTS time (weeks)<br>Activity type	3.75±0.86<br>46% light exercise, 31.2% moderate <2hrs/wk, 6% moderate or hard exercise >2 hrs/wk<br>18.8±8.5<br>(Exercise) walking, swimming, gym most commonly reported
**Crawford et al. 2020**	United States	TKA	Retrospective review	Patients: 1611<br>Age: 63.9<br>34% male<br>BMI: 33.9<br>27% bilateral	11.4 (5.1-15.9) years	Activity	UCLA	Activity (1-10)	7.74±1.70
**Daugaard et al. 2018**	The Netherlands (TKA data)	TKA	Cross-sectional study	Patients: 52<br>Age: 66±7.2<br>50% male<br>BMI: 29.5±4.6	5 years	Activity	Accelerometer	Number of steps<br>Number of short walking bouts (<10sec)<br>Number of transfers (sit/stand)	6702±3203 steps/day, n.s. compared to healthy adults<br>48.7±25.9, significantly less than healthy adults<br>43±16.8, n.s. compared to healthy adults
**Delfin et al. 2017**	Sweden	THA	Matched control study	Patients: 27<br>Age: 72.6±7.1<br>37% male<br>BMI: 27.2±4.1	12.6±5.3 years	Activity	UCLA<br>Questionnaire	Activity (1-10), median IQR<br>Intensity (1: no PA, 5: VPA >1x/wk)	6 [4-7]<br>4 [3-4]

Study	Country	Arthroplasty	Study design	Sample size and Characteristics*	Data collection period (FU & time postop)	Type of sport	Measurement method	Outcome variables of interest	Outcomes
**Donner et al. 2019**	Germany	Bilateral short-stem THA	Case series	Patients: 51<br>Age: 63.1 (36.7-76.8)<br>56.9% male<br>BMI (median): 27.6 (16.6-41.8)<br>100% bilateral	4.9 (4.3-5.8) years	Activity<br>Sports participation	UCLA<br>Questionnaire	Activity (1-10)<br>Participation rate (%)<br>Activity (hours)<br>Sport activity (hours)<br>Frequency (n)<br>Number of activities (n)<br>Activity type	Preop: 3.8±2.0, postop: 4.7±1.7, p= .001<br>Preop 60.8% did sports, postop 76.5%<br>Preop: 3.4, postop: 4.2, p=.26<br>Preop: 3.4±2.4, postop: 3.7±4.1, p=.44<br>Preop: 2.8±3.1, postop: 3.4±2.9, p=.21<br>Preop: 2.3, postop: 1.8.<br>Postop more participation in low-impact, less in high-impact sports compared to preop
**Dubin et al. 2020**	United States	TKA	Case series	Patients: 121, LEAS <10 preop<br>Age: 61.7<br>43% male<br>BMI: 33.2	2.0 years	Activity	LEAS	Activity (1-18)	Preop: 7.1<br>Postop: 8.7
				Patients: 127, LEAS ≥10 preop<br>Age: 61.7<br>49% male<br>BMI: 32.2	2.0 years	Activity	LEAS	Activity (1-18)	Preop: 11.8<br>Postop: 10.8
**Eckhard et al. 2020**	Australia	TKA	Prospective study	Patients: 563 preop<br>Age: 68.0±8.5<br>50% male<br>BMI: 31.3±6.6<br><br>Patients: 381 postop<br>Age: 68.0±8.0<br>47% male<br>BMI: 31.2±6.3	1 year	Activity	UCLA	Activity (1-10)	Preop: 4.3±1.7<br>Postop: 5.9±1.7
**Elman et al. 2014**	Dominica n Republic	THA (22%)<br>TKA (81%)	Case series	Patients: 64<br>Age: 61.1±13.9<br>20% male<br>BMI <35 kg/m2: 23%<br>41% bilateral	2.1 (1-4) years	Activity	YPAS	Mean total time summary index (hours/wk)<br>Mean energy expenditure summary index (kcal/wk)<br>Participate in PA (min/wk)<br>MVPA (min/wk)	23.6±29.1<br><br>5178.9±6552.4<br><br>189.6±233.1<br>84.6±158.9
**Felts et al. 2010**	France	Medial UKA	Therapeutic study	Patients: 62<br>Age: 54.7±5<br>47% male<br>BMI: 28±4<br>5% bilateral	11.2±5 years	Activity<br>Sports participation	UCLA<br>Questionnaire	Activity (1-10)<br>Activity type<br>Sport level<br><br>RTS (%)	6.4±2.3, 40% ≥8<br>Hiking, cycling, swimming most-performed<br>All patients except two returned to their presymptomatic activity level<br>83.4%
**Fisher et al. 2011**	United Kingdom	HRA	Case series	Patients: 117<br>Age: 54 (30-73)<br>57% male<br>14% bilateral	30 (16-50) months	Activity<br>Sports participation	UCLA<br>Questionnaire	Activity (1-10)<br>Participation rate<br>RTS (%)<br><br>Sport type	Preop: 4.4 (1-10); postop 6.8 (3-10), p<.01<br>73%<br>87% of 86 (73%) patients active preop returned to sports, 32% picked up sport<br>Swimming, cycling, golf most popular preop & postop
**Foucher et al. 2018**	United States	THA	Cross-sectional cohort study	Patients: 16<br>Age: 56.8±8.3<br>31% male<br>BMI: 30.9±7.7	23.8 ±10.2 (12-48) months	Activity	UCLA	Activity (1-10)	6±2 (mode: 3)<br>(most common response: 3, 2 out ot 10 subjects (12.5%)

Study	Country	Arthroplasty	Study design	Sample size and Characteristics*	Data collection period (FU & time postop)	Type of sport	Measurement method	Outcome variables of interest	Outcomes
**Fouilleron et al. 2012**	France	HRA	Case series	Patients: 40 runners preop<br>Age: 50.7 (31-61)<br>90% male<br>BMI: 24.8 (21.7-33.6)<br>8% bilateral	33.3 (26-41) months	Running activity	UCLA<br>Devane activity score<br>Questionnaire	Activity (1-10)<br>Activity (1-5)<br>Return to running (%)<br>Number of sports (n)<br>Time jogging (hrs/wk)<br>Distance (km/wk)	Preop: 6.9 (5-10), postop: 9.1 (4-10), p<.001<br>Preop: 3.7 (3-5) postop: 4.6 (2-5), p<.001<br>91.6%, mean 16.4 weeks postop<br>Preop: 4.2, postop: 3.8, p=.196<br>3.1<br>Preop: 38.9, postop: 28.4
**Fujita et al. 2013**	Japan	THA	Case-control series	Patients: 38<br>Age: 60.9±9.1<br>0% male<br>BMI: 23.0±3.6<br>13.2% bilateral	12 months	Activity	Pedometer	Steps/day, % healthy controls<br>LPA (min/day), % healthy controls<br>MPA (min/day), % healthy controls<br>VPA (min/day), % healthy controls	Preop: 4632±2246, 65%; 79%; 12mo: 6163±2410, 85%<br>Preop: 107±49, 78%; 12mo: 125±42, 90%<br>Preop: 16±18, 27%; 12mo: 46±50, 77%<br>Preop: 1±3, 8%; 12mo: 3±4, 25%
**Gerhardt et al. 2017**	The Netherlands	HRA	Prospective cohort study	Patients: 38<br>Age: 54.4±9.4<br>55% male<br>BMI: 26.1±3.1	1, 2, 3 years	Activity	UCLA	UCLA (1-10)	Preop: 5.0 (4.0-7.0)<br>1yr: 7.5 [6.5-9.0]<br>2yr: 7.0 [7.0-8.5]<br>3yr: 7.0 [6.5-8.0]
**Ghom-rawi et al. 2017**	United States	THA (52%)<br>TKA (48%)	Retrospective cohort study	THA: 403 patients (85 had 2yr FU)<br>Age: 65.5±10.8<br>41% male<br><br>TKA: 364 patients (69 had 2yr FU)<br>Age: 67, 1±9.4<br>33% male	2 years	Activity	UCLA<br><br>LEAS	Activity (1-10)<br><br>Activity (1-18)	THA: Preop: 4.4±2.1, postop: 6.0±2.0<br>TKA: Preop: 4.5±2.1; postop: 5.9±2.1<br>THA: Preop: 9.4±3.1, postop: 11.6±3.3<br>TKA: Preop: 9.1±2.8, postop: 10.9±3.0
**Girard et al. 2013**	France	HRA	Prospective case series	Patients: 50 did high-impact sports preop<br>Age: 51.5 (30.8-64.8)<br>90% male<br>BMI: 23.7 (21.7-33.6)<br>10% bilateral	44.1 (39.1-54.5) months	Activity<br>Sports participation	UCLA<br>Questionnaire	Activity<br>RTS (%)<br>RTS time (wk)<br>Sport type<br>Number of activities (n)<br>Time spent (<2, 2-4, 4-7, >7), (hrs/wk)	Preop: 6.6 (4-10), postop: 9.1 (8-10), p< .001<br>98% (82% high-impact)<br>14,6 (7-29)<br>Jogging, soccer most-performed of the high-impact sports<br>Preop: 4 (1-7), postop: 3.6 (1-8)<br>Preop: 3.2 (1-8), postop: 2.9 (2-9)
**Groen et al. 2012**	The Netherlands	TKA	Observational study	Patients: 830<br>Age: 72±9<br>25% male<br>0% bilateral	(1-6) years	Activity	SQUASH	Activity (min/wk)<br>Meeting health recommendation (%)<br>Meeting fitness recommendation (%)<br>Meeting health & fitness recommendation (%)	1337±1260<br>51%<br><br>53%<br><br>46%
**Hara et al. 2018**	Japan	THA	Cohort study	Patients: 524<br>Age: 62.9±10.1<br>16% male<br>BMI: 22.9±3.3<br>19% bilateral	68.2 months	Activity<br>Sports participation	UCLA<br>Questionnaire	Activity<br>Sports participation and impact<br>Sport type	Preop: 3.8±2.1; postop: 4.6±1.7, p<.001<br>Preop: 15.5%, postop: 30.5%, mostly in low-impact sport, 2.5% high impact<br>Most-reported: walking, swimming, gymnastics

Study	Country	Arthroplasty	Study design	Sample size and Characteristics*	Data collection period (FU & time postop)	Type of sport	Measurement method	Outcome variables of interest	Outcomes
**Hayashi et al. 2012**	Japan	THA G2 stem	Prospective cohort study	Patients: 43<br>Age: 66.7±8.2<br>21% male<br>BMI: 23.7±3.1<br>16% bilateral	12 months, 24 months	Activity	UCLA	Activity (0-10)	1yr: 5.4±2.1<br>2yr: 6.3±2.2
		THA VerSys FMT stem	Prospective cohort study	Patients: 15<br>Age: 57.8±6.8<br>BMI: 22.5±2.2<br>20% male<br>0% biltateral	12 months, 24 months	Activity	UCLA	Activity (0-10)	1yr: 6.3±1.5<br>2yr: 6.3±1.5
**Hayashi et al. 2016**	Japan	THA	Prospective cohort study	Patients: 65<br>Age: 65.1±10.4<br>17% male<br>BMI: 23.4±3.5	24 months	Activity	UCLA	Activity (0-10)	5.9±1.6
**Hayes et al. 2011**	Australia	TKA	Cross-sectional study	Patients: 16<br>Age: 64.4±4.6<br>50% male<br>BMI: 69.5±2.6	12 months	Activity	Intelligent device for energy expenditure and activity (IDEEA)	Energy expenditure (j/min/kg)<br>Activity (% recording interval)	103<br><br>4.6%
**Heiberg et al. 2016**	Norway	THA	Long-term FU of RCT (controls)	Patients: 30<br>Age: 70.6±8.4<br>56.7% male	5 years	Activity<br>Sports participation	UCLA<br>Self-reported	Activity (1-10)<br>Training sessions (n/wk)	7±1.9<br>3±1.0
**Hepperger et al. 2018**	Austria	TKA	Prospective single-cohort study	Patients: 200<br>Age: 72±7.7<br>40% male<br>BMI: 27.2±5.0<br>17.5% bilateral	1 year<br>2 years	Activity<br><br>Sports participation	Tegner activity scale<br>Questionnaire	Activity, median [25%, 75%], (1-10)<br>Frequency (none, occasionally, 2-3x/wk, >5x/wk)<br>Sport type<br><br>Sport impact (highest):	12mo: 3.0 [3.0, 4.0]; 24mo: 3.0 [3.0, 4.0]<br>12mo: 18% none, 13% occasionally, 59% 2-3x/wk, 10% >5x/wk<br>24mo: 17% none, 13% occasionally, 57% 2-3x/wk, 13% >5x/wk)<br>Summer: swimming, hiking, cycling<br>Winter: Hiking, skiing, cross-country skiing<br>Summer: low: 9%; medium: 86%, high: 5%<br>Winter: low: 3%; medium: 43%, high: 54%
**Hjorth et al. 2018**	Denmark	MoM THA<br>MoM HRA<br>MoP THA	Prospective cohort study	MoM THA/HRA: 77<br>Age at FU: 59.1 (51.3-64.4)<br>66% male<br><br>MoP THA: 71<br>Age at FU: 68.3 (60.9-69.9)	MoM THA/HRA baseline: 7.1 (4.6-7.6) years<br><br>MoP THA baseline: 8.9 (4.7-10.7) years?<br><br>And baseline + 3, 6, 9, 12 months	Activity	Accelerometer	Mean time spent on walking, bicycling or high-impact activities during daily wear time (%)	MOM THA/HRA:<br>BL + 3m: 12.66% [11.62-13.69]<br>BL + 6m: 11.82% [10.81-12.83]<br>BL + 9m: 11.12% [10.19-12.05]<br>BL+ 12m: 13.15% [12.20-14.09]<br><br>MoP THA:BL + 3m: 13.43% [12.13-14.72]<br>BL + 6m: 12.33% [10.50-14.17]<br>BL + 9m: 10.94% [9.35-12.53]<br>BL+ 12m: 13.34% [11.66-15.03]

Study	Country	Arthroplasty	Study design	Sample size and Characteristics*	Data collection period (FU & time postop)	Type of sport	Measurement method	Outcome variables of interest	Outcomes
**Ho et al. 2016**	United States	TKA<br>UKA	Retrospective cohort study	TKA group<br>Patients: 39<br>Age: 59 (57-64)<br>30% male<br>BMI: 32.5 [28.8-38.4]<br>3% bilateral	4.0±1.2 years	Activity<br>Sports participation	UCLA<br>Phone interview	Activity (1-10)<br>Participation (%)<br>RTS (%), time to RTS (mo), new sport (%)<br>Frequency<br>Duration<br>Sport type	6.1±1.4<br>70%<br>83%, 4 [2-12], 8%<br><br>High range, most 4x or 12x/wk<br>30 min most common<br>Cycling, dancing, swimming most-performed
				UKA group:<br>Patients: 33<br>Age: 60 (53-64)<br>33% male<br>BMI: 30.3 [27.6-33.7]<br>9% bilateral	4.0±1.2 years	Activity<br>Sports participation	UCLA<br>Phone interview	Activity (1-10)<br>Participation (%)<br>RTS (%), time to RTS (mo), new sport (%)<br>Frequency<br>Duration<br>Sport type	7.4±1.6<br>72%,<br>87%, 2 [1.5-4.9], 0%<br><br>4x/wk most common<br>60 min/session most common<br>Golf, cycling, hiking most-performed
**Hodges et al. 2018**	Australia	TKA	Secondary analysis of cohort study	Patients: 434<br>Age (at FU): 66.0±6.3<br>47% male	12 months	Activity	Active Australia Survey	Meeting activity guidelines<br>≥6 hrs/day sedentary<br>Inadequate PA and sedentariness ≥6 hrs/day	47%<br>45%<br>24%
**Hylkema et al. 2020**	The Netherlands	TKA	Observational repeated-measures study	Patients: 57 employees working ≥24 hrs/wk<br>Age: 59±4 (48-65)<br>47% male<br>BMI: 20% normal, 33% overweight, 47% obese	1 year	Activity	Accelerometer	Sedentary (% wear time)<br>Prolonged sedentary bouts, ≥30min (% wear time)<br>LPA (% wear time)<br>MVPA (% wear time)<br>Meeting guidelines, 150 min/wk of MVPA (%)	60.1%<br>16.5%<br><br>36.8%<br>3.1%<br>70%
**Ibrahim et al. 2019**	Sweden	THA	Prospective cohort study	Patients: 42<br>Age female: 68.5 (54-85)<br>Age male: 68 (50-80)<br>48% male	5 years	Activity	Tegner activity scale	Activity (1-10), median and range	Female:<br>Preop: 1 (0-3), postop: 2 (1-4)<br>Male:<br>Preop: 1 (0-4); postop: 2 (1-5)
**Innmann et al. 2016**	Germany	THA	Retrospective cohort study	Patients: 86<br>Age: 52 (21-60)<br>61% male<br>BMI: 27 (18-39)<br>4% bilateral	11 (10-12) years	Activity<br><br>Sports participation	UCLA<br><br>Schultness Clinic sports and activity questionnaire	Activity (1-10)<br>Intense activity (≥7), (%)<br>Sports participation (%)<br>Disciplines (n)<br>Frequency (times/wk)<br>Duration (min)<br>Sport type	Preop: 3.8±1.6; postop: 6.2±1.5, p< .001<br>41%<br>79%, 11% exercising preop stopped sports, 20% non-sporters preop started exercising<br>Preop: 1.8; postop: 1.7<br>Preop: 2.3; postop: 2.6<br>Preop: 53, postop: 55<br>Walking most-performed, most other low-impact sports follow with 10-20% participation rate
**Issa et al. 2015**	United States	TKA	Prospective case series	Patients: 281<br>Age: 66 (39-80)<br>38% male<br>2% bilateral	1, 2, 3, 4, 5 years	Activity	Lower extremity activity scale	Activity (1-18)	Preop: 9.18, 1yr: 11.19, 2yr: 11.49; 3yr: 11.19; 4yr: 11.11; 5yr: 11.47
**Jacquet et al. 2020**	France	UKA	Retrospective case-control study	Patients: 50 did high-impact sports preop<br>Age: 50.8±4.4	3.9±1.8 (2-5.7) years	Sports participation	UCLA	UCLA<br>RTS time (months)<br>Return to impact activities (UCLA >8) (%)	6.5±2<br>5.8±4.2<br>28%<br><br>High tibial osteotomy superior to UKA considering RTS, sport impact and UCLA score.

Study	Country	Arthroplasty	Study design	Sample size and Characteristics*	Data collection period (FU & time postop)	Type of sport	Measurement method	Outcome variables of interest	Outcomes
**Jahnke et al. 2015**	Germany	UKA	Case series	Patients: 135<br>Age: 67.1 (38-88)<br>53.4% male<br>9% bilateral	2±1.47 years	Activity<br><br>Sports participation	UCLA (1-10)<br>Tegner (1-10)<br>Heidelberg Sports Activity Score (HAS)	Activity (1-10)<br>Activity (1-10)<br>HAS<br>Sports participation<br>Sport type	6.33±1.08, small increase<br>3.96±0.96, no change<br>29.29±18.48, high increase<br>93.2%, 84% 1x/wk or more<br>Hiking, cycling, swimming most-performed. High-impact sports participation decreased postop.
**Jassim et al. 2019**	United Kingdom	THR<br>TKR	Case series	THA:<br>Patients: 40<br>Age: 53.1±8.4<br>47% male<br><br>TKA:<br>Patients: 24<br>Age: 60±2.5<br>42% male	THA: 3.3±1.1 years<br>TKA: 3±0.9 years	Activity<br><br><br><br>Sports participation	UCLA<br><br><br><br>Questionnaire	Activity (1-10)<br><br><br><br>RTS<br>Sport type<br>Frequency	14% improved, 11% decreased UCLA. THA patients improved (preop: 7.78; postop: 7.93), TKA patients decreased (preop: 7.5; postop 7.29) activity.<br>100%<br>Golf, running, gym most-performed postop. Remarkable increase in tennis, remarkable decrease in cycling<br>Most participated in sports 4-6x/wk postop .
**Jeldi et al. 2017**	United Kingdom	THA	Observational cohort study	Patients: 27<br>Age: 67 (50-82)<br>33% male<br>BMI: 31 (19-43)	1 year	Activity	Activity monitor	Steps/day	6155±2631
**Jelsma et al. 2019**	The Netherlands	MoM THA	Cohort study	Patients: 62<br>Age: 60.8±9.3<br>58% male<br>BMI: 27.6 (20.2-37.9)	6.3 (3.7–9.6) years	Activity	Accelerometer	Time walking (s/day)<br>Time cycling (s/day)<br>Steps (n/day)<br>High intensity (# peaks)	5403±2472 (1646–17126)<br>282±531 (0–2040)<br>6694±3011 (1712–15443)<br>78.4±197.5 (0–1406)
**Jelsma et al. 2020a & Jelsma et al. 2020b**	The Netherlands	THA<br>HRA	Observational matched cohort study	Patients: 16 HRA<br>Age: 55.5±9.7 (43-67)<br>75% male<br>BMI: 26.1±3.8 (22-37)<br>0% bilateral	10 (9-11) years	Activity	SQUASH<br>Accelerometer	Total activity score (MET/wk), median<br>Time standing (hrs), median<br>Time sitting (hrs), median<br>Time walking (hrs), median<br>Time cycling (hrs), median<br>Steps (n/day), median<br><br>Sit-stand transfers (n), median<br>Time active %	6150 (1110-18480)<br><br>3.0 (1.8-5.7)<br>7.6 (4.6-12)<br>1.3 (0.5-1.9)<br>0.05 (0-0.48)<br>5546 (2274-9966), 31% < 5000, 13% 8000-10000, 6% >10000<br>39 (21-74)<br>10.8±7.0
				Patients: 16 THA<br>Age: 60 (53-68)<br>75% male<br>BMI: 29 (20-40)<br>0% bilateral	10 (8-12) years	Activity	SQUASH<br>Accelerometer	Total activity score (MET/wk), median<br>Time standing (hrs), median<br>Time sitting (hrs), median<br>Time walking (hrs), median<br>Time cycling (hrs), median<br>Steps (n), median<br>Sit-stand transfers (n), median	4560 (1050-9300)<br><br>3.0 (1.6-6.2)<br>9.6 (3.8-13)<br>1.1 (0.4-0.8)<br>0.01 (0.0-1.2)<br>4600 (1567-11749), 50% <5000, 19% ≥8000<br>37 (24-65)
**Jonas et al. 2019**	United Kingdom	HRA	Matched control study	Patients: 51<br>Age 49.8 (18-67)<br>78% male<br>BMI 25.7 (19.7-35.1)	17.6±0.53 years	Activity	UCLA	Activity (1-10)	8 [6-10]
		Hybrid THA	Matched control study	Patients: 53<br>Age 50.4 (21-66)<br>75% male<br>BMI 27.0 (18.5-37.0)	19.9±0.62 years	Activity	UCLA	Activity (1-10)	6 [5-7]

Study	Country	Arthroplasty	Study design	Sample size and Characteristics*	Data collection period (FU & time postop)	Type of sport	Measurement method	Outcome variables of interest	Outcomes
**Jones et al. 2012**	United States	TKA	Prospective cohort study	Patients: 83<br>Age: 66±9.7<br>46% male<br>0% bilateral	1 year	Activity<br><br><br><br>Sports participation	Historical Leisure Activity Questionnaire (HLAQ)<br><br>HLAQ	Activity (MET hrs/wk)<br><br><br><br><br>Sport type<br><br>Sport intensity	21.4±24.9 (0-125.6) MET hrs/wk: significantly more than preop but significantly less than predicted by patients preop.<br>Walking, calisthenics, gardening, strength-training most-reported.<br>Almost all activities performed at moderate intensity (3-5.9 MET), 19.6 of 21.4 MET hrs/wk<br>1.5±6.5 MET at high-intensity<br>0.1±0.9 (0-7.5) at low-intensity
**Karampinas et al. 2017**	Greece	SMF (38%)<br>BFH (33%)<br>HRA (31%)	Retrospective comparative non-blinded study	Patients: 48<br>Age: 65 (52-70)<br>60% male<br>15% bilateral	2 years	Activity<br><br>Sports participation	UCLA<br><br>Questionnaire	Activity (1-10)<br><br>Impact level of sports (low, medium, high)	BFH: 6.7; HRA: 8.1; SMF: 7.9. All improved compared to preop.<br>Most patients in all groups participated in low-impact activities.
**Keeney et al. 2014**	United States	TKA	Therapeutic study	Young patients (≤55): 181<br>Age: 49.0<br>26% male<br>BMI: 34.4<br>21% bilateral<br><br>Old patients (65-75): 262<br>Age: 69.9<br>40% male<br>BMI: 31.5<br>20% bilateral	Young: 36 (12-118) months<br><br><br><br><br>Old: 31 (12-110) months	Activity	UCLA	Sedentary (1-2), mildly active (2-4), moderately active (5-7), highly active (8-10)	Young: Mean UCLA score was 4.6, 57% improved activity level, 14% decreased activity level.<br>56% sedentary or mildly active, 10% returning to regular activity<br><br>Old: Mean UCLA score was 4.9, 65% improved activity level, 14% decreased activity level. 52% sedentary or mildly active, 12% returning to regular activity
**Keeney et al. 2015**	United States	THA	Therapeutic study	Young patients (≤50): 704<br>Age: 39.0<br>51.1% male<br>BMI: 29.1±6.8<br>17% bilateral<br><br>Old patients (65-75): 484<br>Age: 69.4<br>40.3% male<br>BMI: 29.0±5.5<br>7% bilateral	Young: 52 (12-136) months<br><br><br><br>Old: 35 (12-156) months	Activity	UCLA	Sedentary (1-2), mildly active (2-4), moderately active (5-7), highly active (8-10)	Young: Mean UCLA score was 6.4±2.2. 81% did at least in moderate activities, 37% did impact activities.<br><br>Old: Mean UCLA score was 5.3±1.9, 61% did at least moderate activities, 15.5% did impact activities.
**Kersten et al. 2012**	The Netherlands	TKA	Cohort study	Patients: 830<br>Age: 72.0±9.3<br>25.9% male<br>BMI: 29.4±5.0	3.0±1.2 years	Activity	SQUASH	Total activity (min/wk)<br>LPA (min/wk)<br>MPA (min/wk)<br>HPA (min/wk)<br>Household (min/wk)<br>Sport activities (min/wk)<br>Walking (min/wk)<br>Cycling (min/wk)<br>Meeting guidelines (%)	1347±1278<br>780±874<br>337±577<br>223±374<br>715±794<br>52±140<br>167±335<br>122±242<br>50.8

Study	Country	Arthroplasty	Study design	Sample size and Characteristics*	Data collection period (FU & time postop)	Type of sport	Measurement method	Outcome variables of interest	Outcomes
**Kim et al. 2016**	South Korea	Short-stem THA	Case series	Patients: 400<br>Age: 53±13<br>66% male<br>BMI: 29±4<br>33% bilateral	Short stem: 17.8 (13-20) years	Activity	UCLA	Activity (1-10)	8.6 (8-10)
		Ultra-short stem THA	Case series	Patients: 201<br>Age: 53±9<br>59% male<br>BMI: 30±4<br>10% bilateral	Ultra-short stem: 12.3 (10-13) years	Activity	UCLA	Activity (1-10)	9 (8-10)
**Kim et al. 2019**	South Korea	UKA	Prospective comparative study	Patients: 42<br>Age: 63.6±5.5<br>17% male<br>BMI: 25.3±2.4<br>0% bilateral	24 months	Activity<br><br>Sports participation	UCLA<br>Tegner<br>Questionnaire	Activity (1-10)<br>Activity (1-10)<br>Sports participation %<br>Time to return (months)<br>Number of sport<br>Sessions (n/wk)<br>Session duration<br>Level of impact	~5.1<br>~3.6<br>76.2%<br>3.8 months postop<br>2.2±0.8<br>3.0±1.3<br>1.3±0.7<br>Decrease in high-impact activities, increase in swimming & gymnastic riding
**Kiran et al. 2019**	United Kingdom	HRA	Prospective case series	Patients: 66<br>Age: 45.7 (36.8-62.8)<br>62% male<br>BMI: 27.65±4.3<br>9% bilateral	1 year, 10.6±0.5 years	Activity	UCLA	Activity (1-10)	1yr: 8.07±0.93<br>10yr: 7.07±1.16<br>All patients were able to return to their pre-arthritic level of activity, this included some high-level amateur sports.
**Kleeblad et al. 2020**	United States	UKA	Case series	Patients: 164<br>Age: 62.3±8.8<br>55% male<br>BMI: 27.6±4.4<br>9% bilateral	20.2 months	Activity<br>Sports participation	UCLA<br>Questionnaire	Activity (1-10)<br>Participation (%)<br>RTS (%)<br>Level of sport<br>Time to return (months)<br>Number of sports (n)<br>Sport type<br><br>Impact level (% participants)	Preop: 5.93±2.19; postop: 6.78±1.92<br>89.6 %<br>95%<br>42% increased, 43% similar<br>4.43±3.54<br>3.6±3.0<br>Cycling (45%), swimming (38%) most-performed<br>32.7% performed high-impact sports, 63.9% medium-impact, 93.9% low-impact
**Krantz et al. 2012**	France	HRA	Case series	Patients: 22<br>Age: 24.9 (17.1-29.9)<br>31% male<br>BMI: 24.2 (18.8-36.2)<br>9% bilateral	50.6 (44-59) months	Activity	UCLA	Activity (1-10)	Preop: 5.5 (1-9); postop: 7.6 (1-10)
**Krych et al. 2017**	United States	UKA	Therapeutic study	Patients: 183<br>Age: 49.2<br>55% male<br>BMI: 32.4	1, 2, 5, 5.8 years	Activity	Tegner activity scale	Activity (1-10)	Preop (n=183): 2.65<br>1yr (n=76): 4.07<br>2yr (n=133): 4.33<br>5yr (n=67): 4.48<br>5.8yr (n=48): 4.48<br>UKA scored higher than proximal tibial osteotomy at 3 months, 2 years and final FU (5.8yr for UKA, 7.2yr for proximal tibial osteotomy)

Study	Country	Arthroplasty	Study design	Sample size and Characteristics*	Data collection period (FU & time postop)	Type of sport	Measurement method	Outcome variables of interest	Outcomes
**Kuhn et al. 2013**	United States	THA	Prospective case series	Patients: 37<br>Age: 42.1±7.7 (17.8-50.3)<br>32% male<br>BMI: 29.0±5.6 (20.1-44.3)	1.3±0.2 years	Activity	UCLA<br>Stepwatch activity monitor	Activity (1-10)<br>Daily strides<br>Inactive time (%/day)<br>Low-intensity (%/day)<br>Medium-intensity (%/day)<br>High-intensity (%/day)	Preop: 6.0±2.2, postop: 7.2±1.6<br>Preop: 4541±1594; postop: 5584±1626<br>Preop: 74.8%, postop: 71.1%±6.1, p<.001<br>Preop: 16.8%, postop: 18.7%±4.1, p=.004<br>Preop: 7.4%, postop: 8.8%±2.7, p=.001<br>Preop: 1.0%, postop: 1.4%±0.9, p=.006
**Le Duff & Armstutz, 2011**	United States	HRA	Prospective case series	Patients: 201<br>Age: 49.6<br>74.6% male<br>BMI: 26.9 (19-46)<br>28% bilateral	1.8 (1.0-4.9) years<br>9.1 (5.0-13.4) years	Activity<br>Sports participation	UCLA<br>Questionnaire	Activity (1-10)<br>Sports participation<br>Number of activities<br>Activity type<br>Activity impact<br>Activity level<br>Frequency and duration	1.8yr: 7.8; 9.1yr: 7.7<br>1.8yr: 96%; 9.1yr: 90%, p= .50<br>1.8yr: 2.47; 9.1yr: 2.13, p= .02<br>Walking, cycling, weightlifting, swimming, golf most popular at both measurement times<br>High-impact represented 12.4% after 1.8yr and 17.5% and 9.1yr, p=.045<br>Competitive playing in 5.5% after 1.8yr and 10.1% after 9.1yr; 33.9% after 1.8yr and 33.3% after 9.1yr participated in their favorite activity >12x/month, with 45% and 36% of sessions lasting 30 and 60 min, respectively.
**Lefevre et al. 2013**	France	THA<br>TKA	Cross-sectional study	THA group:<br>Patients: 27<br>Age: 63±7.2<br>33% bilateral<br><br>TKA group:<br>Patients: 8<br>Age: 72.8±5.2<br>25% bilateral	THA: 8.8±7.1 years<br><br>TKA: 7±5.9 years	Judo	Mailed survey	Return to judo<br>Time to return<br>Intensity<br>Frequency (times/wk)	THA: 81.5%; TKA: 62.5%<br>THA: 3.9±2.7; TKA: 5.2±2.2<br>All participants stopped competitive judo<br>2.5±1
**Lo Presti et al. 2019**	Italy	Medial UKA	Prospective case series	Patients: 53 did ≥1 sport preop<br>Age: 59.7 (46-66)<br>28.3% male	48±6 (24-72) months	Sports participation	Questionnaire	RTS (%)<br>Sport type<br>Impact (high/low)<br>Frequency (times/wk)<br>Session length (min)<br>Intensity	90%, 13.2% within 3 months, 35.8% within 6 months<br>Cycling (29%), swimming (31%) most-commonly reported.<br>Preop. 32% participated in high-impact sport; postop. 21%<br>Preop: 2.1; postop: 1.9<br>Preop: 50; postop: 43<br>None played professional sports, all sports performed as recreational activities
**Long et al. (2014)**	United States	TKA	Therapeutic study	Patients: 88 (36 filled out Tegner scale)<br>Age: 51 (22-55)<br>25% bilateral	8 (3-18) years<br>25.1 (20-35) years	Activity	Tegner activity scale	Activity (1-10)	Preop: 1.5;<br>8yr: 3.5±1.1;<br>25yr: 3.0±1.6<br>Patients with no contralateral hip pain or knee pain had a 3.5 Tegner score at both time points
**Lubbeke et al. 2014**	Switzerland	THA (97%)<br>HRA (3%)	Prospective single-cohort (BL to 5yr comparison) + cross-sectional study (10yr FU)	5yr FU (n=1085):<br>Age: 67.1±11.7<br>44.6% male<br>BMI: 27.0±4.6<br><br>10yr FU (n=757):<br>Age: 65.1±11.0<br>45.8% male<br>BMI: 26.7±4.2	5 years, 10 years	Activity	UCLA	Activity (1-10),<br>UCLA ≥7 (%)	Preop: 3.5, 4.9%;<br>5yr: 5.7, 27.9%;<br>10yr: 5.5, 28.2%

Study	Country	Arthroplasty	Study design	Sample size and Characteristics*	Data collection period (FU & time postop)	Type of sport	Measurement method	Outcome variables of interest	Outcomes
**Lutzner et al. 2014**	Germany	TKA	Prospective randomized study	Patients: 97<br>Age: 68.9 (CI: 67-70)<br>54% male<br>BMI: 31.3 (CI: 30.3-32.3)	1 year	Activity	Accelerometer (activPAL activity monitor)	Steps/day<br>MVPA (steps/day)<br>Time spent lying (hrs/day)<br>Time spent sitting and standing (hrs/day)<br>Walking time (hrs/day)<br>Meeting guidelines (%)<br>Comparison with healthy population	Preop: 5278±2777; postop: 6473±3654<br>Preop: 1150±982; postop: 1935±1728, ~19 min/day<br>Preop: 12.2±2.4; postop: 12.2±2.7<br>Preop: 10.8±3.7; postop: 10.3±2.6<br><br>Preop: 1.4±1.3; postop: 1.5±0.8<br>Postop: 16.5%<br>In the healthy population more steps were taken in total and in MVPA
**Lutzner et al. 2016**	Germany	TKA	Case series	Patients: 221<br>Age: 68.1±9.5<br>43.4% male<br>BMI: 31.3±4.9	1 year	Activity	Accelerometer (activPAL activity monitor)	Steps/day<br>MVPA (steps/day)<br>Time spent lying (hrs/day)<br>Time spent sitting and standing (hrs/day)<br>Walking time (hrs/day)<br>Meeting guidelines (%)<br>Lifestyle (<5000 sedentary, 5000-7500 low-active, >7500 active)	Preop: 5371±2820; postop: 6587±3299;<br>Preop: 1149±1128; postop: 1835±1646<br>Preop: 12.3±2.3; postop: 12.0±2.4<br>Preop: 10.6±2.9; 10.4±2.3<br><br>Preop: 1.4±1.0; postop: 1.6±0.7<br>22.6%<br>Preop: 48.0% sedentary, 34.8% low-active, 17.2% active; postop: 34.8% sedentary; 34.4% low-active, 30.8% active; p<.001
**Madrid et al. 2019**	Colombia	THA	Cross-sectional study	Patients: 535<br>Age: 67 (13-91)<br>31% male<br>BMI: 25.5±3.9	>1 year	Sports participation (institutionalized)	Phone survey	Sports participation<br>RTS (%), time (months)<br>Sport type<br><br>Sports activity (<5, 5-10, 10-20, >20), (hrs/wk)<br>Level	Preop: 13.5%; postop: 7.1%<br>44%, 2.60±1.2 (1-4)<br>Golf, hiking, swimming most-performed. Increase in hiking, decrease in jogging, soccer, tennis<br>Preop: 39%, 35%, 25%, 3%; postop: 42%, 37%, 21%, 0%<br><br>71% perceived performance equal or better postop
**Majewski et al. 2014**	Switzerland	THA	Cohort study	Patients: 64<br>Age: 60<br>Male: 42 (65.6%)	>10 years FU<br><br>Mean FU 148 months (120-191)	Sports activity	Sports activity index	Sports activity (0-6= non-active; 7-10= moderately active; 11-25= active)	Mean: 13 points<br>Nonactive: 31% (13% scored 0/2, 11% scored 3/4, 7% 5/4)<br>Moderately active: 38% (11% scored 7/8, 26% scored 9/10)<br>Active: 31% (16% scored 11/12, 11% scored 13/14, 11% scored 15/16, 2% 17/18
**Malcolm et al. 2014**	United States	THA	Cross-sectional study	Young patients (≤30): 70<br>Age: 23.3 (12.8-30.7)<br>40% male<br>41% bilateral	6.6 (2.1-14.9) years	Activity	UCLA	Activity (1-10)	6.5±2.3
				Old patients (>30): 158<br>Age: 63.6 (33.6-91.5)<br>49% male<br>23% bilateral	5.3 (2.0-10.8) years	Activity	UCLA	Activity (1-10)	6.4±2.0
**Martin et al. 2018**	United Kingdom	HRA	Case series	Patients: 80<br>Age: 54±8.5<br>100% male	1 year	Activity	UCLA	Activity (1-10)	Preop: 5.4±2.1; postop: 7.2±1.7; p<.001
**Matsunaga-Myoji et al. 2020a**	Japan	TKA	Prospective case series	Patients: 58<br>Age: 72.6±6.0<br>16% male<br>BMI: 26.1±4.4<br>58.6% bilateral	2 years	Activity	Accelerometer	Meeting PA guidelines for older persons (MVPA ≥52.5 min/wk) (%)<br>Light PA (min/wk)<br>MVPA (min/wk)<br>Steps (steps/day)	41.4%<br><br><br>Preop: 267 (159-368); postop: 330 (241-469)<br>Preop: 8.2 (1.3-29.7); postop: 42.8 (10.0-89.2)<br>Preop: 3542 (1958-4828); postop: 4687 (3147-7293)

Study	Country	Arthroplasty	Study design	Sample size and Characteristics*	Data collection period (FU & time postop)	Type of sport	Measurement method	Outcome variables of interest	Outcomes
**Matsunaga-Myoji et al. 2020b**	Japan	THA	Prospective case series	Patients: 107<br>Age: 61.4±8.1<br>14% male<br>BMI: 23.1±3.5<br>0% bilateral	1 year<br>3 years	Activity	Accelerometer	Steps (steps/day)<br>MVPA (min/wk)<br>Meeting guideline of 150min/wk MVPA (%)	Preop: 4776±2259; 1yr: 6634±3242; 3yr: 6736±3150<br>Preop: 35.4±35.1; 1yr: 58.3±64.6; 3 yr: 72.3±67.4<br>Preop: 2.0%, 3yr: 18.0%
**Mayr et al. 2015**	Germany	TKA	Retrospective case series	Patients: 81<br>Age at FU: 71.8±5.4<br>47% male<br>BMI: 28.4	6.4±0.9 years	Sports participation	Questionnaire	Sport type<br>Frequency (n/wk)<br>Duration (hrs/wk)<br>Sport impact	Biking, swimming, mountain hiking most performed sports<br>Preop: 2; postop: 3.5<br>Preop: 3.3; postop: 5.3<br>25% participated in high-impact sports, 47% in medium-impact sports, 52% in low-impact sports
**Meding et al. 2012**	United States	TKA	Retrospective therapeutic study	Patients: 62<br>Age: 63.8±8.9<br>27% male<br>58% bilateral	21.1±1.6 years	Activity	UCLA	Activity (1-10)	8.3±1.2 (5-10)<br>35% UCLA 9 or 10
**Mesko & Heath 2011**	USA	CoC-THA	Cohort study	Patients: 62<br>Age: 52 (26-70)<br>62.5% male<br>BMI: 29.5 (20-50)<br>7% bilateral	121 (98-168) months	Activity	UCLA	Activity (1-10)<br>High-impact (UCLA 9/10), (very) active (UCLA 7/8), inactive/moderately active (UCLA 1-6)	6.6±2.04<br>16% performed high-impact sports, 35% highly active. 48% inactive to moderately active
**Naylor et al. 2019**	Australia	TKA<br>THA	Prospective case series	Patients: 1289<br>Age: 67.2±9<br>45% male<br>BMI: 30.9±6<br>5% bilateral	3 years	Activity	Phone interview	Participation in PA ≥1x/wk (%)<br>Sport type	THA: Preop: 50.6; postop: 67.3, p< .001<br>TKA: pre: 40.8; postop: 60.6, p< .001<br>Walking performed most (47%). General increase in low-impact activities (walking, cycling, gym, swimming), general decrease in high-impact activities (tennis, squash, jogging)
**Ninomiya et al. 2018**	Japan	THA	Cross-sectional controlled study	N: 58<br>Age: 68.0±5.4<br>17.3% male<br>BMI: 22.1±2.8<br>100% unilateral	10.4±0.4 years	Activity	IPAQ	Activity (high ≥1000kcal, low <1000kcal)	High: 34.4%, low: 65.5%<br>Healthy control group: High: 69.6%, low: 30.4%
**Ollivier et al. 2014**	France	THA	Retrospective case series	Patients: 571<br>Age: 61.3±10.9<br>Gender: 52% male<br>BMI: 27±3.2<br>0% bilateral	9.8±2.9 years	Activity<br>Sports participation	UCLA<br>Questionnaire	Activity (1-10)<br>Sports participation<br>RTS time (months)<br>Sport type	6.8±2.2 (1-10)<br>64%<br>6.6±3.2 (2-48)<br>Highest frequencies in low-impact sports, 26.6% participated in non-recommended activities
**Ortmaier et al. 2017**	Austria	Short-stem THA	Retrospective case series	Patients: 137<br>Age: 65.6±12.4<br>BMI: 26.6±4	20.4±2.3 (18-22) months	Activity<br>Sports participation	UCLA<br>Questionnaire	Activity (1-10)<br>Participation (%), RTS (%)<br>Time to RTS<br>Disciplines (n)<br>Sport type<br><br>Frequency (0, 1, 2, 3, 4, >4x/wk), (%)<br>Session length (0-15, 15-30, 30-60, 60-120, >120 minutes), (%)<br>Sport level	7.1 (4-10)<br>Preop: 92%; postop: 87%, RTS: 91%<br>62% within 3mo, 86% within 6mo<br>Preop: 2.9±2.1; postop: 2.6±1.9<br>Swimming, hiking, cycling, nordic walking, alpine skiing most-performed, decrease in most high-impact sports<br>18%, 12%, 20%, 14%, 9%, 27%.<br><br>>4 n/wk preop: 18%; postop: 27%, p<.01, 24%, 9%, 15%, 22%, 29%<br><br>>80% reported participation in recreational sports

Study	Country	Arthroplasty	Study design	Sample size and Characteristics*	Data collection period (FU & time postop)	Type of sport	Measurement method	Outcome variables of interest	Outcomes
**Panzram et al. 2018**	Germany	Medial UKA	Retrospective cohort study	Patients: 27<br>Age: 62.5±8.3 (49-76)<br>56% male<br>11% bilateral	60±8.3 (47-69) months	Activity<br><br><br>Sports participation	UCLA<br><br><br>Tegner<br>Schulthess Clinical Activity Questionnaire	Activity (1-10)<br>High, medium, low (>6, 4-6, <4)<br>Activity (1-10)<br>Participation (%), RTS (%)<br>RTS time<br>Sport type	Preop: 4.9±2.3; postop: 6.1±1.8, p<.05<br>37%, 51%, 11%<br><br>Preop: 2.9±1.4; postop: 3.4±1.0<br>89%, 100%<br>67% returned within 3 months<br>Cycling, hiking, long walks most-performed. Impact sports decreased, barely different participation in low-impact sports
**Panzram et al. 2020**	Germany	UKA	Retrospective case series	Patients: 177<br>Age at FU: 64.4±9.7 (38-82)<br>BMI at FU: 30.9±5.4<br>8% bilateral	37.1±9.8 (24-60) months	Activity<br><br><br>Sports participation	UCLA<br><br>Tegner<br>Schulthess Clinical Activity Questionnaire	Activity (1-10), UCLA ≥7 (%)<br>Activity (1-10)<br>Sports participation (%)<br>RTS (%), STS (%)<br>Disciplines (n)<br>Frequency (n), >4x/wk ≥ 60 min (%)<br>Sport type	Preop: 2.9±1.7 (1-10); postop: 6.3±1.4 (2-10), 53.7%<br>3.5±1.0 (0-7)<br>83.4%<br>92.9%, 35.7%<br>Preop: 2.8±2.2; postop: 2.0±1.8<br>Preop: 2.8±1.3; postop: 2.8±1.4<br>15.6%<br>Low numbers in high-impact sports; cycling & walking most-performed
**Paxton et al. 2016**	United States	THA	Cohort study	Patients: 5678<br>Age: 67 [60-75]<br>41% male<br>62% BMI <30<br>0% bilateral	1-2 years	Activity	Questions by nursing staff	Activity (min/wk)<br>Meeting guidelines	Preop: 50 [0-140]; postop: 150 [60-280]<br>50%
		TKA	Cohort study	Patients: 11084<br>Age: 68 [62-75]<br>38% male<br>49% BMI <30<br>0% bilateral	1-2 years	Activity	Questions by nursing staff	Activity (min/wk)<br>Meeting guidelines	Preop: 58 [3-143]; postop: 150 [60-280]<br>50%
**Payo-Ollero et al. 2020**	Spain	THA	Case series	Patients: 46<br>Age: 41 (37-48)<br>72% male<br>BMI: 26.1 (24.5-29)<br>25% bilateral	7.5 (1-11) years	Activity<br>Sports participation	UCLA<br>Phone questionnaire	Activity (1-10)<br>Sports participation (%)<br>Number of sports, 1, 2, 3, ≥4 (%)<br>Sport type	Preop: 6.85±3.01; postop: 6.22±2.24<br>Preop: 74%; postop: 78%<br>Preop: 20%, 24%, 11%, 20%; postopp: 28%, 28%, 11%, 11%<br>Swimming, walking, cycling most-often performed
**Pietsch-mann et al. 2013**	Germany	Medial UKA	Case series	Patients: 131<br>Age: 65.3 (44-83)<br>44% male<br>6% bilateral	4.2 (1-10) years	Activity<br>Sports participation	UCLA<br>Questionnaire	Activity (1-10)<br>Participation (%)<br>RTS (%)<br>Frequency<br>Disciplines<br>Impact<br><br>Sport type	6<br>Preop: 60%, postop: 53%<br>80.1%, 11% of non-sporters started<br>Increase in frequency<br>Preop: 1.73, postop: 1.74<br>Shift from high- to low-impact (7 participated in impact sport preop, 3 postop)<br>Cycling most popular activity
**Pioger et al. 2020**	France	TKA	Cross-sectional study	Patients: 143 active golfers<br>Age: 65.7±(43-85)<br>85.3% male<br>0% bilateral	4.6 (2.0-14.2) years	Activity<br><br>Golf	UCLA<br><br>Questionnaire	Activity (1-10)<br><br>Time to RTS (18-hole golf course), (months)<br>Playtime (hrs/wk)<br>Sport level (handicap)	Preop: 7.02±2.04; postop: 7.85±1.14; p< .01<br>3.7 (0.7-2.5)<br>Preop: 8.9±6.1; postop: 10.2±6.6; p<.01<br>Preop: 25.2±13.1; Postop: 24.4±13.3; p=.01

Study	Country	Arthroplasty	Study design	Sample size and Characteristics*	Data collection period (FU & time postop)	Type of sport	Measurement method	Outcome variables of interest	Outcomes
**Pisanu et al. 2020**	Italy	TKA PS (89%)<br>TKA CNH (5%)<br>TKA CR (3%)<br>UKA (4%)	Retrospective case series	Patients: 118<br>Age: 73 [67-77]<br>28.7% male<br>BMI: 29.1±4.7<br>3% bilateral	3.1±1.1 years	Sports participation	Phone questionnaire	Participation (%)<br>RTS, STS (%)<br>Time to RTS, (months)<br>Disciplines (n)<br>Activity type	87%,<br>93%, 50%<br>4 [3-6]<br>Preop: 1.1; postop: 1.1<br>Most performed low-impact activities, mainly walking. No decrease in high- or medium-impact sports
**Plassard et al. 2020**	France	TKA	Retrospective case series	Patients: 443<br>Age: 69 (41-90)<br>36.6% male<br>BMI: 29.3 (19-46)	43 [23-49] months	Activity<br><br><br>Sports participation	UCLA<br><br><br>Questionnaire	Activity (1-10)<br>Activity (%), low: UCLA ≤3, moderate 4-6, high ≥7<br>Sports participation (%)<br>Sport type	Preop: 4.45; postop: 5.92<br>Preop: 27%, 64%, 8%<br>Postop: 12%, 55%, 33%<br>85%<br>Walking, hiking, swimming, hiking most-performed
**Plate et al. 2013**	United States	HRA (33%)<br>CoC THA (33%)<br>MoP THA (33%)	Therapeutic study	Patients: 3x30 matched groups<br>Age:<br>HRA & MoP THA: 53 (37-79)<br>CoC THA: 52 (39-69)<br>37% male	HRA: 38 (24-66)<br>MoP THA: 29 (24-60)<br>CoC THA: (24-62) months	Activity	Weighted activity score questionnaire	Activity participation at recreational or professional level (0-9 low, >9 high)	HRA: 10.5 (1-28)<br>CoP THA: 5.6 (1-18)<br>CoC THA: 6.9 (0-34)<br>HRA significantly higher than CoP and CoC THA.
**Ponzio et al 2018**	USA	TKA	Matched case-control study	Patients:1008 LEAS >12 preop (N=772 at FU)<br>Age: 66.3±9.1<br>56.6% male<br>BMI: 28.3±5.0	2 years	Activity	LEAS	Activity (1-18)	Preop: 14.6±1.2<br>Postop: 13.7±2.7
				Patients: 1008 LEAS 7-12 preop (N=727 at FU)<br>Age: 66.3±9.0<br>56.6% male<br>BMI: 28.4±4.9	2 years	Activity	LEAS	Activity (1-18)	Preop: 9.1±1.7; postop: 11.6±2.9
**Poortinga et al. 2014**	The Netherlands	THA (64%)<br><br>TKA (36%)	Retrospective cohort study	Patients: 658<br>Age: 68±10.6<br>32% male<br>BMI: 28.7±4.9	1 year	Activity	SQUASH	Total PA (min/wk)<br>Leisure-time PA (min/wk)<br>Meeting guideline (%)	Preop: 1140 [975-2040]; postop: 1525 [1200-1980]; p=.118<br>270 [120-570]; postop: 420 [210-720]; p<.001<br>Preop: 29%; postop: 40%; p=.005
**Postler et al. 2017**	Germany	THA	Comparative study	Patients: 124<br>Age: 64.1±11.0<br>29.8% male<br>BMI: 27.5±3.7	2.3±0.3 years	Activity	UCLA	Activity (1-10)	Preop: 4.2±1.4; postop: 5.2±1.7
**Pritchett 2018**	United States	Tripolar THA	Prospective cohort study	Patients: 160 needing unrestricted activity<br>Age: 43 (19-76)<br>48% male	11 (6-19) years	Activity<br><br>Sports participation	UCLA<br><br>Questionnaire	Activity (1-10)<br><br>Sport type	Preop: 4 (2-7); postop: 8.8 (6-10), p<.001<br>80% UCLA ≥8<br>Many high-impact activities or participation at high level stated

Study	Country	Arthroplasty	Study design	Sample size and Characteristics*	Data collection period (FU & time postop)	Type of sport	Measurement method	Outcome variables of interest	Outcomes
**Ristolainen et al. 2019**	Finland	TKA	Retrospective cohort study	Patients: 70, knee injury as cause of TKA<br>Age: 51.8±5.2<br>BMI: 29.0±5.0<br><br>Sports-related: 18<br>Age: 50.0±5.3<br>39% male<br>BMI: 27.8±4.1<br>44% bilateral<br><br>Other causes: 45<br>Age: 52.2±5.3<br>56% male<br>BMI: 29.9±5.5<br>29% bilateral	All: 6.2±3.7 years<br><br>Sports-related: 4.5±3.0 years<br><br>Other: 6.6±3.7 years	Activity<br><br>Sports participation	Mailed questionnaire<br>Mailed questionnaire	Leisure-time PA (MET hrs/wk)<br>Participation<br><br>Intensity<br><br>Session duration<br>Frequency	Sport: 42.1; Other: 18.5<br><br>Sport: preop: 100%, postop: 89%<br>Other: preop: 71%, postop: 74%<br>Total: 68% walking intensity, 14% high-intensity activity.<br>Sports group participated more often at higher intensity<br>>50% patients 1 hour<br>>50% exercised >10x/month
**Robertson et al. 2016**	United States	THA (35%)<br>TKA (15%)<br><br>HRA (50%)	Case series	Patients: 13 active<br>Age: 48.7 (37-61)<br>77% male<br>BMI: 23.9 (20.6-28.5)<br>54% bilateral	8.7 (1.8-15.8) years	Activity	Accelerometer	Cycles/day<br>Maximum cycles/day (over mean of 9.4 days)<br>Cycles/year<br>High PA cycles (%)<br>Moderate PA cycles (%)<br>Low PA cycles (%)<br>High PA (%), (min/day)<br>Moderate PA (%) (min/day)	8273 (5973-12557)<br>11794 (7925-16983)<br><br>3.019.737 (2.176.704-4.583.461)<br>39.7 (8-62)<br>42.6 (27.1-63.9)<br>18.7 (10.7-30.6)<br>4.3%, 58 (11.5-118.1)<br>9.4%, 135 (83.5-204.5)
**Rolving et al. 2013**	Denmark	THA	Historical cohort study	Patients: 95<br>Age: 72.3±6.2<br>33% male	22.4 (18.4-23.9) months	Activity<br><br>Activity/ Sports participation	Physical activity scale<br>Questionnaire	Physical activity scale (MET/day), >40 moderate<br>Time spent on leisure activities (min/wk)<br>Activity type	41 [38.5; 48.5]<br><br>480 [240; 870]<br><br>Walking (79%), bicycling (48%)
**Rozenlund et al. 2017**	Denmark	THA	Randomized controlled trial	Patients:<br>Lateral approach: 38<br>Age: 60±7<br>68% male<br>BMI: 27±3<br><br>Posterior approach: 39<br>Age: 62±6<br>67% male<br>BMI 28±4	12 months	Activity	UCLA	Activity (1-10)	Lateral approach:<br>Preop: 5 [4-7]; postop: 6.5<br>Posterior approach:<br>Preop: 5 [3-6]; postop: 7.3

Study	Country	Arthroplasty	Study design	Sample size and Characteristics*	Data collection period (FU & time postop)	Type of sport	Measurement method	Outcome variables of interest	Outcomes
**Ruecknl et al. 2020**	United States	THA	Prospective study	Patients: 39, desired UCLA ≥8<br>Age: 48.3±6.1<br>100% male<br>BMI: 27.5±3.7<br>18% bilateral	56.7±13.9 months	Activity	UCLA<br>LEAS	Activity (1-10)<br>Activity (1-18)	Preop: 5.8±2.4; postop: 7.7±2.0<br>14.1±2.5
		HRA	Prospective study	Patients: 34, desired UCLA ≥8<br>Age: 47.6±5.2<br>100% male<br>BMI: 27.6±3.3<br>3% bilateral	54.4±21.3 months	Activity	UCLA<br>LEAS	Activity (1-10)<br>Activity (1-18)	Preop: 6.0±2.3; postop: 9.4±2.1<br>15.9±1.5
**Sandberg et al. 2019**	United States	TKA	Matched case-control study	Patients: 183<br>Age: 72.1±7.8<br>27.9% male<br>BMI: 33.1±6.4	12.9 months	Activity	UCLA	Activity (1-10)	TKA:<br>Preop: 3.90±1.57; postop: 5.12±1.68<br>6.2% UCLA >7
		Dual-pivot TKA	Matched case-control study	Patients: 183<br>Age: 67.8±7.9<br>27.2% male<br>BMI: 33.4±6.5	12.6 months	Activity	UCLA	Activity (1-10)	Dual-pivot TKA:<br>Preop: 4.03±1.72; postop: 5.31±1.85<br>12.2% UCLA >7<br>No significant differences between standard TKA and dual-pivot TKA
**Sandiford et al. 2015**	United Kingdom	HRA	Case series	Patients: 79 active<br>Age: 54.9 (34.5-73.6)<br>67% male<br>BMI: 25.2 (19.8-31.9)<br>1% bilateral	8.5 (8-10) years	Activity<br>Sports participation	UCLA<br>Questionnaire	Activity (1-10)<br>RTS (%)<br>RTS time<br>Disciplines (n), >1 (%)<br>Sport type<br>Sport level<br>Frequency (n/wk)<br>Session duration (min)	Preop: 4 (1-9); postop: 7.6 (2-10)<br>6 months: 100%, 8.5yr: 97%<br>85% returned within 3 months, 100% within 6 months<br>2 (1-5): 37% performed more disciplines postop.<br>Walking, swimming. aerobics most-frequently performed<br>14% competitive, 86% recreational<br>All >3x/wk<br>All 60-90
**Schmidutz et al. 2012**	Germany	Short-stem THA	Case series	Patients: 68<br>Age: 55±12<br>Gender: 60% male<br>BMI: 26±4 kg/m2<br>12% bilateral	2.7±0.7 (2.0-4.2) years	Activity<br>Sports participation	UCLA<br>Questionnaire	Activity (1-10)<br>Participation, RTS (%)<br>RTS time (1-2, 3-4, >5 mo)<br>Disciplines (n)<br>Sport impact<br>Sport type<br><br><br>Frequency, (n/wk)<br>Session duration (min)	7.6±1.9 (3-10)<br>91%, 98%<br>26%, 25%, 47%<br>Preop: 3.9±2.4; 3.5±2.0<br>41% fewer disciplines, 59% equal or more disciplines<br>Decrease in high- and medium-impact sports, 41% participating in high- or medium-impact activities<br>Cycling, hiking, swimming most popular<br>Preop: 1.5±0.9; postop: 1.8±1.1; p=.022<br>Preop: 67±35; postop: 66±33; p=.753
**Schneider et al. 2020**	United States	TKA (50%)<br>medial UKA (32%)<br>lateral UKA (19%)	Retrospective cohort study	Patients: 92 did sports preop<br>Age: 56±9.1<br>13% male<br>BMI: 28±5.6	1-2 years	Activity<br>Sports participation	UCLA<br>Questionnaire	Activity (1-10)<br>UCLA ≥7 (%)<br>Sport impact (low, medium, high), (number of sports)	TKA:<br>Preop: 5.26±2.4; postop: 6.62±1.9<br>Preop: 39.2%; 60.8%<br>2.61±1.6; 1.00±0.9; 0.33±0.7
						Activity<br>Sports participation	UCLA<br>Questionnaire	Activity (1-10)<br>UCLA ≥7 (%)<br>Sport impact (low, medium, high), (number of sports)	UKA:<br>Preop: 5.93±2.5; postop: 6.89±2.0<br>Preop: 32.6%; 54.3%<br>2.82±2.0; 1.07±1.0; 0.48±0.7

Study	Country	Arthroplasty	Study design	Sample size and Characteristics*	Data collection period (FU & time postop)	Type of sport	Measurement method	Outcome variables of interest	Outcomes
**Scott et al. 2017**	United Kingdom	TKA	Case series	Patients: 289<br>Age: 59 (42-65)<br>49% male<br>BMI: 31.4 (15-55)<br>8% bilateral	3.3 (2-4) years	Activity	UCLA	Activity (1-10) (Median)	Preop: 4<br>Postop: 6<br>58% improved<br>46% ≥7
**Smith et al. 2017 (T)**	United Kingdom	THA (57%)<br>TKA (43%)	Prospective cohort study	Patients: 201<br>Age: 72.9±10.7<br>38% male<br>0% unilateral	2-4 years	Activity	Face-to-face interviews	Summary index (sedentary, mild, moderate, high)	Preop: 7.5% (5-12); 17.4% (13-23); 61.7% (55-68); 13.4% (9-18)<br>Postop: 12.9% (9-18); 20.4% (15-27); 54.7% (48-61); 11.9% (8-17)
**Smith et al. 2018 Does PA change**	United Kingdom	THA<br>TKA	Prospective case-control study	THA patients: 105<br>Age: 68.2±9.3<br>42.9% male<br>BMI: 28.8±4.2<br><br>TKA patients: 116<br>Age: 67.3±8.3<br>41.4% male<br>BMI: 30.1±4.9	12 months, 24 months	Activity<br><br><br><br>Sports participation	PASE<br><br><br><br>PASE	Total score<br><br><br><br>Light sport/ recreation frequency (never, 1-2, 3-4, >4 days)<br>Moderate sport/ recreation frequency (never, 1-2, 3-4, >4 days)<br>Strenuous sport/ recreation frequency (never, 1-2, 3-4, >4 days)	THA preop: 136; 12mo: 135; 24mo: 132<br>TKA preop: 126; 12mo: 121; 24mo: 142 TKA preop to 24 mo significant increase<br>No differences with control group<br>THA 24 mo: 87.5%, 9.7%, 2.8%, 0%<br>TKA 24 mo: 84.5%, 13.6%, 1.0%, 1.0%<br>THA 24 mo: 94.4%, 2.8%, 1.4%, 1.4%<br>TKA 24 mo: 87.4%, 6.8%, 4.9%, 0.9%<br>THA 24 mo: 73.6%, 4.2%, 13.9%, 8.3%<br>TKA 24 mo: 66%, 8.7%, 18.4%, 6.9%
**Stambourgh et al. 2014**	USA	TKA	Matched case-control study	Patients: 76<br>Age: 48.7 (26-55)<br>30% male<br>BMI: 33.9 (14.9-54.9)<br>7% bilateral	Mean FU 4.8 (2-13.9) years	Activity	UCLA	Activity (1-10)	Preop: 3.47 (1-9)<br>Postop: 4.71 (2-10)
**Takenaga et al 2013**	USA	THA	Cohort study	Patients: 55<br>Age: 39 (18-50)<br>65% male<br>BMI: 29 (18-42)<br>5% bilateral	12.1 (10-16) years	Activity	UCLA<br>Tegner<br>Step Activity Monitor	Activity (1-10)<br>Activity (1-10)<br>Steps/year	6.1 (2-10)<br>3 (0-6)<br>1.56 million steps (77,000-3.26 million)
**Takeuchi et al. 2020**	Japan	THA	Retrospective case series	Patients: 204<br>Age: 53.7 (30-60)<br>18% male<br>18% bilateral	59.5 months	Activity	UCLA	Activity (1-10)	Preop: 4.55; postop: 6.17; p<.01<br>Preop highest UCLA was 6, achieved by 16.7%, but postop 62.3% had UCLA 6 and 18.6% >7
**Vielgut et al. 2016**	Austria	TKA	Retrospective case series	Patients: 236<br>Age: 62.7±11.4<br>18% male<br>10% bilateral	14.9±3.0 years	Activity<br>Sports participation	Tegner score	Activity (1-10)<br>Participation, RTS (%)<br>Sport impact<br><br>Sport type	Preop: 2.18±1.5; postop: 3.04±1.5<br>70.8%, 70.8%<br>71.3% continued participating in low-impact, 43.7% medium-impact, 16.7% high-impact sports<br>Low-impact activities most-performed, especially cycling & hiking.
**Von Rottkay et al. 2018**	Germany	THA	Prospective cohort study	Patients: 64<br>Age: 63.0<br>53% male<br>BMI: 27.1	12 months	Activity	Daily activity questionnaire<br>Step counter	Load cycles/day<br>Steps/day	Preop: 3727±947; postop: 4226±948,<br>Preop: 5541±2048; postop: 5658±2213

Study	Country	Arthroplasty	Study design	Sample size and Characteristics*	Data collection period (FU & time postop)	Type of sport	Measurement method	Outcome variables of interest	Outcomes
**Wagenmakers et al. 2011**	The Netherlands	THA	Prospective multicentre cohort study	Patients: 653<br>Age: 70.3±8.2<br>26% male<br>BMI: 27.0±4.1	52.4±3.9 weeks	Activity	SQUASH	Activity (min/wk)<br>LPA (min/wk)<br>MPA (min/wk)<br>VPA (min/wk)<br>Meeting guidelines (%)	1468.1±1138.3<br>Most time spent in household (756.3±766.5) and leisure (584.5±657.8) activities<br>805.1±800.4<br>333.6±508.0<br>329.4±458.5<br>67%
**Walker et al. 2015a (return to sports)**	Germany	Lateral UKA	Retrospective case series	Patients: 45<br>Age: 60.1±10.5<br>42% male<br>BMI: 27<br>0% bilateral	3 (2.0-4.3) years	Activity<br><br>Sports participation	UCLA<br>Tegner score<br>Schulthess Clinic Activity score	Activity (1-10), ≥7 (%)<br>Activity (1-10)<br>Participation, RTS (%)<br>RTS time (<3, <6, >6 mo)<br>Disciplines (n)<br>Frequency (n/wk)<br>>1-hr session (%)<br><br>Sport impact<br>Sport type	Preop: 5.3±2.3; postop: 6.7±1.5, 66%<br>Preop: 2.6±1.6; postop: 3.5±0.8<br>Preop: 93%; postop: 95.6%, RTS: 97.8%<br>55%, 77.8%, 22.2%<br>Preop: 3; postop: 3<br>Preop: 3; postop: 3<br>Preop: 52%; postop: 44%<br>Decrease in high-impact activities, increase in low-impact activities<br>Biking, walking/hiking most performed
**Walker et al. 2015b (Sports, PA)**	Germany	Medial UKA	Retrospective case series	Patients: 93<br>Age: 55 (36-60)<br>48% male<br>BMI: 32 (20-58)<br>17% bilateral	4.4±1.6 (2.3-8.4) years	Activity<br><br>Sports participation	UCLA<br>Tegner score<br>Schulthess Clinic Activity score	Activity (1-10), ≥7 (%)<br>Activity (1-10)<br>Participation (%)<br>RTS; STS (%)<br>RTS time (<1, <3, <6, >6 mo)<br>Disciplines (n)<br>Frequency (n/wk)<br>>1-hr session (%)<br>Sport impact<br><br>Sport type	Preop: 3.3±1.5; postop: 6.8±1.5; 62%<br>Preop: 2.0±1.1; postop: 3.8±1.1<br>Preop: 93%, postop: 91%;<br>93%; 6%<br>27%; 56%; 77%; 23%<br>Preop: 4, postop: 3.3<br>53% participated in ≥3 sessions/wk<br>45%<br>Decrease in high-impact activities, increase in low-impact activities. 29% participated in high-impact sports<br>Biking, walking, swimming most-performed
**Webber et al. 2017**	Canada	TKA	Cross-sectional study	Patients: 38<br>Age: 67.9±7.3<br>42% male<br>BMI: 30.5±6.1	1 year	Activity	Activity monitor	Steps (steps/day)<br>Sedentary, (hrs/day); (%/wear time)<br><br>LPA (min/day)<br>MVPA (min/day)	Preop: 3724±2338; postop: 5935±3316<br>Preop: 9.3±1.4, postop: 9.2±1.4, p=.62<br>Preop: 66.9%±9.0, postop: 63.8%±10.0; p=.18<br>Preop: 264.0±73.3; postop: 291.7±91.0<br>Preop: 0±1.8; postop: 2.1±10.4<br>Patients had a higher cadence postop
**Wimmer et al. 2015**	United States	TKA	Cross-sectional cohort study	Patients: 32<br>Age: 77.8±6.1<br>31% male<br>BMI: 29.9±5.7	11.5±3.3 years	Activity	Activity monitor and goniometer	Lying (%)<br>Sitting (%)<br>Standing (%)<br>Level-walking (%)<br>Stair-walking (%)<br>Unrecognized (%)<br>Steps (steps/12hrs)	7.8%±10.5<br>59.9%±14.7<br>12.7%±5.8<br>7.8%±3.8<br>0.1%±0.1<br>11.7%±3.6<br>3102±1553, of which 2.1% stairs
**Wollmerstedt et al. 2010**	Germany	THA	Cross-sectional cohort study	Patients: 59<br>Age: 58 (37-77)<br>50% male	5 years	Activity	Pedometer Daily Activity Questionnaire (DAQ)	Steps (steps/day)<br>Steps (steps/day)	Preop: 4967±2154; 5yr: 6564±2795<br>Preop: 4665±2053; 5yr: 6375±2971
				Patients: 75<br>Age: 70 (52-86)<br>48% male	10 years	Activity	UCLA<br>Pedometer Daily Activity Questionnaire (DAQ)	Activity (1-10)<br>Steps (steps/day)<br>Steps (steps/day)	Preop: 6±1; postop: 6±1<br>5101±2412<br>5210±2738

**Zimmerer et al. 2021**	Germany	Lateral UKA	Case series	Patients: 19<br>Age: 26.7±10.5 (36.0-76.0)<br>26% male<br>BMI: 27.2±5.7 (17.3-38.0)	4.6±2.2 (2.0-9.7) years	Activity<br><br>Sports activity	UCLA<br><br>Schulthess Clinic Activity Score	Activity (1-10)<br>UCLA ≥7 (%)<br>Participation (%)<br>RTS (%), STS (%)<br>Disciplines (n)<br>Sport frequency (n/wk)<br>Session length (min)<br>Sport type	Preop: 4.1±1.7 (2-9); postop: 6.4±1.3 (4-9)<br>47%<br>68%<br>86%, 0%<br>Preop: 2.8; postop: 2.6<br>Preop: 2.7; postop: 2.3<br>Preop: 41.5; postop: 45.9<br>Long walks & biking most-performed. High-impact sports (soccer, skiing) had decreased participation rates postop. Nordic walking & jogging significantly increased.

*At time of surgery, unless indicated otherwise; ? Unknown/not reported.

Abbreviations: BFH: big femoral head; BMI: body mass index; CNH: constrained non-hinged; CoC: ceramic-on-ceramic; CoP: ceramic-on-polyethylene; CR: cruciate-retaining; FU: follow-up; HPA: high physical activity; HRA: hip-resurfacing arthroplasty; IPAQ: International Physical Activity Questionnaire; IQR: interquartile range; LEAS: Lower Extremity Activity Scale; LPA: low physical activity; MET: metabolic equivalent of task; MoM: metal-on-metal; MoP: metal-on-polyethylene; MPA: moderate physical activity; MVPA: moderate-to-vigorous physical activity; PA: physical activity; PASE: Physical Activity Scale for the Elderly; postop: postoperatively; preop: preoperatively; PS: posterior stabilized; RTS: return to sports; SMF: short metaphyseal; SQUASH: Short QUestionnaire to ASsess Health-enhancing physical activity; STS: start to sport; THA: total hip arthroplasty; TKA: total knee arthroplasty; UCLA: University of California Los Angeles activity scale; UKA: unicompartimental knee arthroplasty; VPA: vigorous physical activity; YPAS: Yale Physical Activity Scale

Grimby scale (range 1-4) 1 sedentary – 4 regular hard physical training for competition sports (vigorous physical activity; LEAS: Lower Extremity Activity Scale (range 1-18) 1 I am confined to my bed all day – 18 I am up and about at will in my house and outside. I also participate in vigorous physical activity such as competitive level sports daily; Sports activity index (range 0-25) 0-6 = non-active; 7-10 = moderately active; 11-25 = active; Tegner activity scale (range 0-10) 0 is on sick leave/disability - 10 is participation in competive sports at a national or international elite level; UCLA: University of California Los Angeles activity scale (range 1-10) 1 wholly inactive: dependent on others; cannot leave residence – 10 regular participation in impact sports such as tennis, acrobatics, ballet, heavy labour or backpacking; Weighted activity score (0-9 low, >9 high)
