# Supplementary material for: Amount and type of physical activity and sports from one year forward after hip or knee arthroplasty—A systematic review
Source: PLoS One. 2021 Dec 28;16(12):e0261784. doi: 10.1371/journal.pone.0261784 (PMC8714096; doi:10.1371/journal.pone.0261784)
Supplement: S5 Appendix — (PDF) [file pone.0261784.s005.pdf]

Appendix 5. Results on amount and intensity of physical activity.

Arthroplasty/Measurement method/Study	Age/BMI/gender	Follow-up time	Outcome
**Total hip arthroplasty**			
Objective meaurement devices			
Alvarez et al. 2015	N: 47<br>Age: 63.8±11.7<br>55% male<br>BMI: 29.6±5.6	>1 year	148.9±69.8
Clement et al. 2019	N: 200<br>Age: 69.9±9.2 (42-92)<br>43% male	12 & 24 months	*Patients aged <65:*<br>Steps: 12 mo: 5370±2524; 24 mo: 4269±2905 steps/day<br>Energy expenditure: 12 mo: 31.1±1.4; 24 mo: 30.4±1.2 MET/day<br>Time spent walking: 12 mo: 1.2±0.5; 24 mo: 1.0±0.6 hrs/day<br>Time spent standing: 12 mo: 3.4±1.6; 24 mo: 3.0±1.3 hrs/day<br>Time spent sitting/lying down: 12 mo: 18.3±2.2; 24 mo: 18.6±2.0 hrs/day<br>*Patients aged 65-74*<br>Steps: 12 mo: 6071±3256; 24 mo: 5760±3199 steps/day<br>Energy expenditure: 12 mo: 32.2±1.7; 24 mo: 31.1±1.5 MET/day<br>Time spent walking: 12 mo: 1.3±0.7; 24 mo: 1.2±0.6 hrs/day<br>Time spent standing: 12 mo: 4.4±2.0; 24 mo: 4.3±2.2 hrs/day<br>Time spent sitting/lying down: 12 mo: 17.6±2.9; 24 mo: 17.6±2.7 hrs/day<br>*Patients aged ≥75*<br>Steps: 12 mo: 4344±2195; 24 mo: 3915±2054 steps/day<br>Energy expenditure: 12 mo: 31.4±1.4; 24 mo: 29.9±1.5 MET/day<br>Time spent walking: 12 mo: 0.8±0.4; 24 mo: 0.9±0.4 hrs/day<br>Time spent standing: 12 mo: 3.8±1.0; 24 mo: 3.3±0.8 hrs/day<br>Time spent sitting/lying down: 12 mo: 18.5±1.3; 24 mo: 18.1±1.3 hrs/day
Fujita et al. 2013	N: 38<br>Age: 60.9±9.1<br>0% male<br>BMI: 23.0±3.6<br>13.2% bilateral	12 months	Steps: 6163±2410 steps/day<br>LPA: 125±42 min/day<br>MPA: 46±50 min/day<br>VPA: 3±4 min/day
Jeldi et al. 2017	N: 27<br>Age: 67 (50-82)<br>33% male<br>BMI: 31 (19-43)	1 year	Steps: 6155±2631 steps/day
Jelsma et al. 2020ab	N: 16<br>Age: 60 (53-68)<br>75% male<br>BMI: 29 (20-40)<br>0% bilateral	10 (8-12) years	Steps: 4600 (1567-11749) steps/day, 50% <5000, 19% ≥8000 steps/day<br>Time cycling: 0.01 (0.0-1.2) hrs/day<br>Time walking: 1.1 (0.4-0.8) hrs/day<br>Time standing: 3.0 (1.6-6.2) hrs/day<br>Time sitting: 9.6 (3.8-13) hrs/day

Arthroplasty/Measurement method/Study	Age/BMI/gender	Follow-up time	Outcome
Kuhn et al. 2013	N: 37<br>Age: 42.1±7.7 (17.8-50.3)<br>32% male<br>BMI: 29.0±5.6 (20.1-44.3)	1.3±0.2 years	Steps: 5584±1626 steps/day<br>Inactive time: 71.1±6.1 %/day<br>Low-intensity: 18.7±4.1 %/day<br>Medium-intensity: 8.8%±2.7 %/day<br>High-intensity: 1.4%±0.9 %/day
Matsunaga-Myoji et al. 2020b	N: 107<br>Age: 61.4±8.1<br>14% male<br>BMI: 23.1±3.5<br>0% bilateral	1 & 3 years	Steps 1yr: 6634±3242 steps/day<br>Steps 3yr: 6736±3150 steps/day<br>MVPA 1yr: 58.3±64.6 min/week<br>MVPA 3yr: 72.3±67.4 min/week<br>Meeting guideline of 150min/week MVPA: 18%
Hjorth et al. 2018	N: 71<br>Age at FU: 68.3 (60.9-69.9)<br>44% male	8.9 (4.7-10.7)<br>FU at 3, 6, 9, 12 months	% spent on walking, bicycling or high-impact activities during wear time<br>FU + 3mo: 13.43% [12.13-14.72]<br>FU + 6mo: 12.33% [10.50-14.17]<br>FU + 9mo: 10.94% [9.35-12.53]<br>FU + 12mo: 13.34% [11.66-15.03]
Takenaga et al. 2013	N: 55<br>Age: 39 (18-50)<br>65% male<br>BMI: 29 (18-42)<br>5% bilateral	12.1 (10-16) years	1.56 million (77,000-3.26 million) steps/year (4274 steps/day)
Von Rottkay et al. 2018	N: 64<br>Age: 63.0<br>53% male<br>BMI: 27.1	12 months	Steps: 5658±2213 steps/day
Wollmerstedt et al. 2010	N: 59<br>Age: 58 (37-77)<br>50% male	5 years	Steps: 6564±2795 steps/day
	N: 75<br>Age: 70 (52-86)<br>48% male	10 years	Steps: 5101±2412 steps/day
Self-reported questionnaires			
Elman et al. 2014	N: 64<br>Age: 61.1±13.9<br>20% male<br>41% bilateral	2.1 (1-4) years	Questionnaire: YPAS<br>Total time summary index: 23.6±39.1 hrs/week<br>Energy expenditure: 5178.9±6552.4 kcal/week<br>Participation in PA: 189.6±233.1 min/week<br>MVPA: 84.6±158.9 min/week
Jelsma et al. 2020ab	N: 16 THA<br>Age: 60 (53-68)<br>75% male<br>BMI: 29 (20-40)<br>0% bilateral	10 (8-12) years	Questionnaire: SQUASH<br>Total activity score: 6150 (1110-18480) MET/week

Arthroplasty/Measurement method/Study	Age/BMI/gender	Follow-up time	Outcome
Ninomiya et al. 2018	N: 58<br>Age: 68.0±5.4<br>17.3% male<br>BMI: 22.1±2.8<br>100% unilateral	10.4±0.4 years	Questionnaire: IPAQ<br>34.4% highly active (≥1000 kcal/week)<br>65.5% low-active (<1000 kcal/week)
Paxton et al. 2016	N: 5678<br>Age: 67 [60-75]<br>41% male<br>62% BMI <30<br>0% bilateral	1-2 years	Questions by nursing staff:<br>Activity: 150 [60-280] min/week<br>Met guidelines: 50%
Rolving et al. 2013	N: 95<br>Age: 72.3±6.2<br>33% male	22.4 (18.4-23.9) months	Questionnaire: Physical activity scale<br>PA: 41 [38.5; 48.5] MET/day<br>Leisure activities: 480 [240; 870] min/week
Smith et al. 2018	N: 105<br>Age: 68.2±9.3<br>42.9% male<br>BMI: 28.8±4.2	12 months<br>24 months	Physical activity scale for the elderly (PASE)<br>Total score: 12 mo: 135; 24 mo: 132
Von Rottkay et al. 2018	N: 64<br>Age: 63.0<br>53% male<br>BMI: 27.1	12 months	Daily activity questionnaire<br>Load cycles: 4226±948 cycles/day
Wagenmakers et al. 2011	N: 653<br>Age: 70.3±8.2<br>26% male<br>BMI: 27.0±4.1	52.4±3.9 weeks	Questionnaire: SQUASH<br>Total activity: 1468.1±1138.3 min/week<br>Leisure activity: 584.5±657.8 min/week<br>LPA: 805.1±800.4 min/week<br>MPA: 333.6±508.0 min/week<br>VPA: 329.4±458.5 min/week<br>67% met activity guidelines
Wollmerstedt et al. 2010	N: 59<br>Age: 58 (37-77)<br>50% male	5 years	Daily activity questionnaire<br>Steps: 6375±2971 steps/day
	N: 75<br>Age: 70 (52-86)<br>48% male	10 years	Daily activity questionnaire<br>Steps: 5210±2738 steps/day

Arthroplasty/Measurement method/Study	Age/BMI/gender	Follow-up time	Outcome
**Hip resurfacing arthroplasty**			
Objective measurement devices			
Jelsma et al. 2020ab	N: 16<br>Age: 55.5±9.7 (43-67)<br>75% male<br>BMI: 26.1±3.8 (22-37)<br>0% bilateral	10 (9-11) years	Steps: 5546 (2274-9966) steps/day, 31% <5000, 13% 8000-10000, 6% >10000 steps/day<br>10.8±7.0 [4.1-18.3] % time spent active<br>Time cycling: 0.05 (0-0.48) hrs/day<br>Time walking: 1.3 (0.5-1.9) hrs/day<br>Time standing: 3.0 (1.8-5.7) hrs/day<br>Time sitting: 7.6 (4.6-12) hrs/day
Self-reported questionnaires			
Jelsma et al. 2020ab	N: 16<br>Age: 55.5±9.7 (43-67)<br>75% male<br>BMI: 26.1±3.8 (22-37)<br>0% bilateral	10 (9-11) years	Questionnaire: SQUASH<br>Total activity score: 6150 (1110-18480) MET/week
**Total knee arthroplasty**			
Objective measurement devices			
Bin Sheeha et al. 2020	N: 33<br>Males: 6<br>Age: 76±7 (63-85)<br>BMI: 32.38±2.01<br><br>Females: 27<br>Age: 59±6 (49-76)<br>BMI: 37.21±7.65	12 months	Steps: 6174±2287 steps/day<br>Stepping time: 1.36±0.38 hrs/day<br>Time standing: 3.54±0.97 hrs/day<br>Upright time: 4.88±1.47 hrs/day<br>Sedentary time: 19.08±1.54 hrs/day<br><br>Time <60 steps/min: 593±188 min/week<br>Time 60-100 steps/min: 457±179 min/week<br>Time >100 steps/min (MVPA): 41.7 min/week<br><br>Met guidelines 150 min MVPA:<br>24% considering all stepping moments<br>12% considering >1-min bouts<br>9% considering >5-min bouts<br>6% considering >10-min bouts
Brandes et al. 2011	N: 53<br>Age: 65.8±5.8<br>34% male<br>BMI: 30.7±4.1	1 year	Gait cycles: 5932±2111 cycles/day<br>Time spent on walking: 12.0±4.7% wear time
Casazza et al. 2020	N: 7<br>Age: 55.6, SEM 3.5<br>39% male<br>BMI: 32.8, SEM 1.5	12 months	Steps: 5981 SEM 751 steps/day<br>Energy expenditure: 2297 SEM 133 kcal/day

Arthroplasty/Measurement method/Study	Age/BMI/gender	Follow-up time	Outcome
Daugaard et al. 2018	N: 52<br>Age: 66±7.2<br>50% male<br>BMI: 29.5±4.6	5 years	Steps: 6702±3203 steps/day
Hylkema et al. 2020	N: 57 employees working ≥24 hrs/week<br>Age: 59±4 (48-65)<br>47% male<br>BMI: 20% normal, 33% overweight, 47% obese	1 year	LPA: 36.8% wear time<br>MVPA: 3.1% wear time<br>Sedentary: 60.1% wear time<br>Prolonged sedentary bouts ≥30 min: 16.5% wear time<br>Meeting guidelines 150 min of MVPA/week: 70%
Lutzner et al. 2014	N: 97<br>Age: 68.9 (CI: 67-70)<br>54% male<br>BMI: 31.3 (CI: 30.3-32.3)	1 year	Steps: 6473±3654 steps/day<br>MVPA steps: 1935±1728 steps/day<br>MVPA steps: ~19 min/day<br>Walking time: 1.5±0.8 hrs/day<br>16.5% met PA guidelines
Lutzner et al. 2016	N: 221<br>Age: 68.1±9.5<br>43.4% male<br>BMI: 31.3±4.9	1 year	Steps: 6587±3299 steps/day<br>MVPA steps: 1835±1646 steps/day<br>Walking time: 1.6±0.7 hrs/day<br>Patient's lifestyle: 34.8% sedentary, 34.4% low-active, 30.8% active
Matsunaga-Myoji et al. 2020a	N: 58<br>Age: 72.6±6.0<br>16% male<br>BMI: 26.1±4.4<br>58.6% bilateral	2 years	Steps: 4687 (3147-7293) steps/day<br>LPA: 330 (241-469) min/week<br>MVPA: 42.8 (10.0-89.2) min/week<br>Met PA guidelines for older persons (MVPA ≥52.5 min/week): 41.4%
Webber et al. 2017	N: 38<br>Age: 67.9±7.3<br>42% male<br>BMI: 30.5±6.1	1 year	Steps: 5935±3316 steps/day<br>Sedentary: 9.2±1.4 hrs/day, 63.8%±10.0 wear time<br>LPA: 291.7±91.0 min/day<br>MVPA: 2.1±10.4 min/day
Wimmer et al. 2015	N: 32<br>Age: 77.8±6.1<br>31% male<br>BMI: 29.9±5.7	11.5±3.3 years	Steps: 3102±1553 steps/12hrs<br>Walking: 7.8%±3.8 of time
Self-reported questionnaires			
Groen et al. (2012)	N: 830<br>Age: 72±9<br>Gender: 27% male<br>0% bilateral	(1-6) years	Questionnaire: SQUASH<br>Activity: 1337±1260 min/week<br>51% met health recommendation<br>53% met fitness recommendation<br>46% met both health and fitness recommendation
Hayes et al. 2011	N: 16<br>Age: 64.4±4.6<br>50% male<br>BMI: 69.5±2.6	12 months	Energy expenditure: 103 joule/min/kg<br>Activity: 4.6% recording interval

Arthroplasty/Measurement method/Study	Age/BMI/gender	Follow-up time	Outcome
Hodges et al. 2018	N: 434<br>Age (at FU): 66.0±6.3<br>47% male	12 months	Questionnaire: Active Australia Survey<br>Met guidelines: 47%<br>≥6 hrs/day sedentary: 45%<br>Inadequate physical activity and sedentary behavior: 24%
Jones et al. 2012	N: 83<br>Age: 66±9.7<br>46% male<br>0% bilateral	1 year	Historical leisure activity questionnaire (HLAQ):<br>Total activity: 21.4±24.9 MET hrs/week<br>Low-intensity: 0.1±0.9 MET hrs/week<br>Medium-intensity: 19.6±23.6 MET hrs/week<br>High-intensity: 1.5±6.5 MET hrs/week
Kersten et al. 2012	N: 830<br>Age: 72.0±9.3<br>25.9% male<br>BMI: 29.4±5.0	3.0±1.2 years	Questionnaire: SQUASH<br>Total activity: 1347±1278 min/week<br>Light PA: 780±874 min/week<br>Moderate PA: 337±577 min/week<br>Vigorous PA: 223±374 min/week<br>Sports activities: 52±140 min/week<br>Walking: 167±335 min/week<br>Cycling: 122±242 min/week<br>50.8% met physical activity guidelines
Paxton et al. 2016	N: 11084<br>Age: 68 [62-75]<br>38% male<br>49% BMI <30<br>0% bilateral	1-2 years	Questions by nursing staff:<br>Activity: 150 [60-280] min/week<br>Met guidelines: 50%
Ristolainen et al. 2019	N: 18 sports injury-related TKAs<br>Age: 50.0±5.3<br>39% male<br>BMI: 27.8±4.1<br>44% bilateral	4.5±3.0 years	Mailed questionnaire:<br>Leisure time PA: 42.1 MET hrs/week
	N: 45 non-sports injury-related TKA<br>Age: 52.2±5.3<br>56% male<br>BMI: 29.9±5.5<br>29% bilateral	6.6±3.7 years	Mailed questionnaire:<br>Leisure-time PA: 18.5 MET hrs/week
Smith et al. 2018	N: 116<br>Age: 67.3±8.3<br>41.4% male<br>BMI: 30.1±4.9	12 & 24 months	Physical activity scale for the elderly (PASE)<br>Total score: 12 mo: 121; 24 mo: 142

Arthroplasty/Measurement method/Study	Age/BMI/gender	Follow-up time	Outcome	
**Unicompartimental knee arthroplasty − Mixed arthroplasty**				
Objective measurement devices				
Blikman et al. (2013)<br>THA/TKA	N: 44<br>Age: 72±9<br>23% male<br>BMI: 28±5.4	>1 year	Mean counts/minute: 210±142<br>Total activity: 99±156 min/week<br>MPA: 92±127 min/week<br>VPA: 7±35 min/week<br>Sitting time: 1252±314 min/week	
Hjorth et al. 2018<br>THA/HRA	N: 77<br>Age at FU: 59.1 (51.3-64.4)<br>66% male	7.1 (4.6-7.6) years and FU + 3, 6, 9, 12 months	% spent on walking, bicycling or high-impact activities during wear time<br>FU + 3m: 12.66% [11.62-13.69]<br>FU + 6m: 11.82% [10.81-12.83]<br>FU + 9m: 11.12% [10.19-12.05]<br>FU+ 12m: 13.15% [12.20-14.09]	
Robertson et al. 2016<br>THA/HRA/<br>TKA	N: 13 active patients<br>Age: 48.7 (37-61)<br>77% male<br>BMI: 23.9 (20.6-28.5)<br>54% bilateral	8.7 (1.8-15.8) years	Cycles: 8273 (5973-12557) cycles/day<br>% high activity cycles: 39.7% (8-62)<br>% moderate activity cycle: 42.6% (27.1-63.6)<br>% low activity cycle: 18.7% (10.7-30.6)<br>High PA: 4.3%, 58 (11.5-118.1) min/day<br>Moderate PA: 9.4%, 135 (83.5-204.5) min/day	
Self-reported questionnaires			Short-form	Long-form
Blikman et al. (2013)<br>THA/TKA	N: 44<br>Age: 72±9<br>23% male<br>BMI: 28±5.4	>1 year	Questionnaire: IPAQ short-form<br>Total activity: 3370±3762 MET min/week<br>Total time: 868±969 min/week<br>MPA: 822±945 min/week<br>VPA: 46±129 min/week<br>Sitting time: 3123±1214 min/week	Questionnaire: IPAQ long-form<br>Total activity: 4498±3840 MET min/week<br>Total time: 1108±922 min/week<br>MPA: 1087±909 min/week<br>VPA: 20±59 min/week<br>Sitting time: 3255±1118 min/week
Poortinga et al. 2014<br>THA/TKA	N: 658<br>Age: 68±10.6<br>32% male<br>BMI: 28.7±4.9	1 year	Questionnaire: SQUASH<br>Total PA: 1525 [1200-1980] min/week<br>Leisure time PA: 420 [210-720] min/week<br>40% meets PA guideline	

*At time of surgery, unless indicated otherwise.

Abbreviations: BMI: body mass index; FU: follow-up; HPA: high physical activity; HRA: hip-resurfacing arthroplasty; IPAQ: International Physical Activity Questionnaire; LPA: low physical activity; MET: metabolic equivalent of task; MPA: moderate physical activity; MVPA: moderate-to-vigorous physical activity; PA: physical activity; PASE: Physical Activity Scale for the Elderly; SEM: standard error of measurement; SQUASH: Short QUestionnaire to ASsess Health-enhancing physical activity; THA: total hip arthroplasty; TKA: total knee arthroplasty; UKA: unicompartimental knee arthroplasty; VPA: vigorous physical activity; YPAS: Yale Physical Activity Scale
