# Supplementary material for: Amount and type of physical activity and sports from one year forward after hip or knee arthroplasty—A systematic review
Source: PLoS One. 2021 Dec 28;16(12):e0261784. doi: 10.1371/journal.pone.0261784 (PMC8714096; doi:10.1371/journal.pone.0261784)
Supplement: S6 Appendix — (PDF) [file pone.0261784.s006.pdf]

Appendix 6. Overview of one-item questionnaires giving a general indication of overall physical activity performed.

Arthroplasty/Measurement Method/Study	Age/BMI/gender	Follow-up time	Outcome
**Total hip arthroplasty**			
UCLA			
Alvares et al. 2015	N: 47<br>Age: 63.8±11.7<br>55% male<br>BMI: 29.6±5.6<br>19% bilateral	>1 year	5.5±1.3
Batailler et al. 2019	N: 32<br>Age: 60.7±9.6<br>66% male<br>BMI: 26±4 kg/m2<br>100% bilateral	20.1±11.6 months	5.6±1.8
	N: 64<br>Age: 61.8±10<br>66% male<br>BMI: 25.9±4 kg/m2<br>0% bilateral	28.9±15 months	4.9±2.2
Breuer et al. 2020	N: 55<br>Age 61±10<br>53% male<br>BMI: 27.9±4.9	38±4.6 months	6.9±1.9
Delfin et al. 2017	N: 27<br>Age: 72.6 (±7.1)<br>37% male<br>BMI: 27.2 (±4.1)	12.6 ±5.3 years	6 [4-7]
Donner et al. 2019	N: 51<br>Age: 63.1 (36.7-76.8)<br>56.9% male<br>BMI (median): 27.6 (16.6-41.8)<br>100% bilateral	4.9 (4.3-5.8) years	4.7±1.7
Foucher et al. 2018	N: 16<br>Age: 56.8±8.3<br>31% male<br>BMI: 30.9±7.7	23.8 ±10.2 (12-48) months	6±2
Ghomrawi et al. 2017	N: 403, 85 had 2yr FU<br>Age: 65.5±10.8<br>41% male	2 years	6.0±2.0
Hara et al. 2018	N: 524<br>Age: 62.9±10.1<br>16% male<br>BMI: 22.9±3.3<br>19% bilateral	68.2 months	4.7±1.7
Hayashi et al. 2012	N: 43, G2 stem<br>Age: 66.7±8.2<br>21% male<br>BMI: 23.7±3.1<br>16% bilateral	12 & 24 months	5.4±2.1<br>6.3±2.2
	N: 15, VerSys FMT stem<br>Age: 57.8±6.8<br>BMI: 22.5±2.2<br>20% male<br>0% biltateral	12 & 24 months	6.3±1.5<br>6.3±1.5
Hayashi et al. 2016	N: 65;<br>Age: 65.1±10.4 17% male<br>BMI: 23.4±3.5	24 months	5.9±1.6
Heiberg et al. 2016	N: 30<br>Age: 70.5±8.4<br>56.7% male	5 years	7±1.9

Arthroplasty/Measurement Method/Study	Age/BMI/gender	Follow-up time	Outcome
Innmann et al. 2016	N: 86<br>Age: 52 (21-60)<br>61% male<br>BMI: 27 (18-39)<br>4% bilateral	11 (10-12) years	6.2±1.5<br>41% UCLA >6
Jassim et al. 2019	N: 40<br>Age: 53.1±8.4<br>47% male	3.3±1.1 years	7.9±1.2 (6-10)
Jonas et al. 2019	N: 53<br>Age: 50.4 (21–66)<br>75% male<br>BMI 27.0 (18.5–37.0)	19.9±0.62 years	6 [5-7]
Keeney et al. 2015	N: 704<br>Age: 39.0<br>51.1% male<br>BMI: 29.1±6.8<br>17% bilateral	52 (12-136) months	6.4±2.2<br>81% UCLA >4<br>37% UCLA >7
	N: 484<br>Age: 69.4<br>40.3% male<br>BMI: 29.0±5.5<br>7% bilateral	35 (12-156) months	5.3±1.9<br>61% UCLA >4<br>15.5% UCLA >7
Kim et al. 2016	Short stem hip arthroplasty:<br>N: 400<br>Age: 53±13<br>66% male<br>BMI: 29±4<br>33% bilateral	17.8 (13-20) years	8.6 (8-10)
	Ultra-short stem hip arthroplasty:<br>Patients: 201<br>Age: 53±9<br>59%<br>BMI: 30±4<br>10% bilateral	12.3 (10-13) years	9 (8-10)
Kuhn et al. 2013	N: 37<br>Age: 42.1±7.7 (17.8-50.3)<br>32% male<br>BMI: 29.0±5.6 (20.1-44.3)	1.3±0.2 years	7.2±1.6
Malcolm et al. 2014	N: 70<br>Age: 23.3 (12.8-30.7)<br>40% male<br>41% bilateral	6.6 (2.1-14.9) years	6.5±2.3
	N: 158<br>Age: 63.6 (33.6-91.5)<br>49% male<br>23% bilateral	5.3 (2.0-10.8) years	6.4±2.0
Mesko & Heath 2011	N: 62<br>Age: 52 (26-70)<br>Male: 55 (62.5%)<br>BMI: 29.5 (20-50)<br>7% bilateral	121 (98-168) months	6.6±2.04<br>48% UCLA <7<br>35% UCLA 7 or 8<br>16% UCLA >8
Ollivier et al. 2014	N: 571<br>Age: 61.3±10.9<br>Gender: 52% male<br>BMI: 27±3.2<br>0% bilateral	9.8±2.9 years	6.8±2.2 (1-10)
Ortmaier et al. 2017	N: 137<br>Age: 65.6±12.4<br>BMI: 26.6±4	20.4±2.3 (18-22) months	7.1 (4-10)
Payo-Ollero et al. 2020	N: 46<br>Age : 41 (37-48)<br>72% male<br>BMI : 26.1 (24.5-29)<br>25% bilateral	7.5 (1-11) years	6.22±2.24

Arthroplasty/Measurement Method/Study	Age/BMI/gender	Follow-up time	Outcome
Postler et al. 2017	N: 124<br>Age: 64.1±11.0<br>29.8% male<br>BMI: 27.5±3.7	2.3±0.3 years	5.2±1.7
Pritchett et al. 2018	N: 160 needing unrestricted activity<br>Age: 43 (19-76)<br>48% male	11 (6-19) years	8.8 (6-10)<br>80% ≥8
Rosenlund et al. 2017	N: 38 lateral approach<br>Age: 60±7<br>68% male<br>BMI: 27±3	12 months	6
	N: 39 posterior approach<br>Age: 62±6<br>67% male<br>BMI: 28±4	12 months	7
Schmidutz et al. 2012	N: 68<br>Age: 55±12<br>Gender: 60% male<br>BMI: 26±4 kg/m2<br>12% bilateral	2.7±0.7 (2.0-4.2) years	7.6±1.9 (3-10)
Rueckl et al. 2020	N: 39, desired UCLA ≥8<br>Age: 48.3±6.1<br>100% male<br>BMI: 27.5±3.7<br>18% bilateral	56.7±13.9 months	7.7±2.0
Takenaga et al. 2013	N: 55<br>Age: 39 (18-50)<br>65% male<br>BMI: 29 (18-42)<br>5% bilateral	12.1 (10-16) years	6.1 (2-10)
Takeuchi et al. 2020	N: 204<br>Age: 53.7 (30-60)<br>18% male<br>18% bilateral	59.5 months	6.2<br>62.3% UCLA 6, 18.6% UCLA >7
Wollmerstedt et al. 2010	N: 75 at FU<br>Age: 70 (52-86)<br>48% male	10 years	6±1
Tegner			
Ibrahim et al. 2019	N: 42<br>Age females: 68.5 (54-85)<br>Age males: 68 (50-80)<br>48% male	5 years	Females: median: 2, range: 1-4<br>Males: median: 2, range: 1-5
Takenaga et al. 2013	N: 55<br>N: 39 (18-50)<br>65% male<br>BMI: 29 (18-42)<br>5% bilateral	12.1 (10-16) years	3 (0-6)
Other			
Clement et al. 2019	N: 200<br>Age: 69.9±9.2 (42-92)<br>43% male	12 & 24 months	*Patients aged <65*<br>LEAS:<br>12 months: 12.5±2.8;<br>24 months: 12.0±3.1<br>*Patients aged 65-74*<br>LEAS:<br>12 months: 11.5±2.9<br>24 months: 11.6±3.2<br>*Patients aged ≥75*<br>LEAS:<br>12 months: 8.7±2.9<br>24 months: 9.0±3.0

Arthroplasty/Measurement Method/Study	Age/BMI/gender	Follow-up time	Outcome
Cowie et al. 2013	N: 239<br>Age: 55.2±7.2<br>35.2% male<br>BMI: 28.2±4.9	3.1±0.97 years	Grimby scale:<br>3.75±0.86
Ghomrawi et al. 2017	N: 403, 85 had 2yr FU<br>Age: 65.5±10.8,<br>41% male	2 years	LEAS:<br>11.6±3.3
Delfin et al. 2017	N: 27<br>Age: 72.6 (±7.1)<br>37% male<br>BMI: 27.2 (±4.1)	12.6 ±5.3 years	Scale (1: no PA, 5 VPA >1x/week):<br>4 [3-4]
Majewski et al. 2014	N: 64<br>Age: 60<br>65.6% male	148 (120-191) months	Sports activity index<br>0-6 = non-active; 7-10 = moderately active; 11-25 = active<br>13 points<br>31% non-active, 38% moderately active, 31% active
Plate et al. 2013	N: 30<br>Age: 52 (39-69)<br>37% male<br>CoC THA:	CoC THA: (24-62) months	Weighted activity score (0-9 low, >9 high):<br>CoC THA: 6.9 (0-34)
Rueckl et al. 2020	N: 39, desired UCLA ≥8<br>Age: 48.3±6.1<br>100% male<br>BMI: 27.5±3.7<br>18% bilateral	56.7±13.9 months	LEAS:<br>14.1±2.5
**Hip resurfacing arthroplasty**			
UCLA			
Fisher et al. 2011	N: 117<br>Age: 54 (30-73)<br>57% male<br>14% bilateral	30 (16-50) months	6.8 (3-10)
Fouilleron et al. 2012	N: 40 preoperative runners<br>Age: 50.7 (31-61)<br>90% male<br>BMI: 24.8 (21.7-33.6)<br>8% bilateral	33.3 (26-41) months	9.1 (4-10)
Gerhardt et al. 2017	N: 38<br>Age: 54.4±9.4<br>55% male;<br>BMI: 26.1±3.1	1, 2, 3 years	1yr: 7.5 [6.5-9.0]<br>2yr: 7.0 [7.0-8.5]<br>3yr: 7.0 [6.5-8.0]
Girard et al. 2013	N: 50 participating in high-impact sport pre-surgery<br>Age: 51.5 (30.8-64.8)<br>90% male<br>BMI: 23.7 (21.7-33.6)<br>10% bilateral	44.1 (39.1-54.5) months	9.1 (8-10)
Jonas et al. 2019	N: 51<br>Age 49.8 (18–67)<br>78% male<br>BMI: 25.7 (19.7–35.1)	17.6±0.53 years	8 [6-10]
Kiran et al. 2019	N: 66<br>Age: 45.7 (36.8-62.8)<br>62% male<br>BMI: 27.65±4.3<br>9% bilateral	1, 10.63±0.54 years	1yr: 8.1±0.93<br>10yr: 7.1±1.16
Krantz et al. 2012	N: 22<br>Age: 24.9 (17.1-29.9)<br>31% male<br>BMI: 24.2 (18.8-36.2)<br>9% bilateral	50.6 (44-59) months	7.6 (1-10)

Arthroplasty/Measurement Method/Study	Age/BMI/gender	Follow-up time	Outcome
Le Duff et al. 2011	N: 201<br>Age: 49.6<br>74.6% male<br>BMI: 26.9 (19-46)<br>28% bilateral	1.8 (1.0-4.9) years,<br>9.1 (5.0-13.4) years	1.8yr: 7.8<br>9.1yr: 7.7
Martin et al. 2018	N: 80<br>Age: 54±8.5<br>100% male	1 year	7.2±1.7
Rueckl et al. 2020	N: 34, desired UCLA score ≥8<br>Age: 47.6±5.2<br>100% male<br>BMI: 27.6±3.3<br>3% bilateral	54.4±21.3 months	9.4±2.1
Sandiford et al. 2015	N: 79<br>Age: 54.9 (3405-73.6)<br>67% male<br>BMI: 25.2 (19.8-31.9)<br>1% bilateral	8.5 (8-10) years	7.6 (2-10)
Other			
Fouilleron et al. 2012	N: 40 preoperative runners<br>Age: 50.7 (31-61)<br>90% male<br>BMI: 24.8 (21.7-33.6)<br>8% bilateral	33.3 (26-41) months	Devane activity score (1-5): 4.6 (2-5)
Banerjee et al. 2010	N: 138<br>Age: 52.6 (38-71)<br>59% male	23.5 (12-42) months	Grimby scale:<br>4.6
Rueckl et al. 2020	N: 34, desired UCLA score ≥8<br>Age: 47.6±5.2<br>100% male<br>BMI: 27.6±3.3<br>3% bilateral	54.4±21.3 months	LEAS:<br>15.9±1.5
**Total knee arthroplasty**			
UCLA			
Bercovy et al. 2015	N: 403<br>Age: 70.36 (40.11-91.2)<br>34% male<br>BMI: 29.6 (19.8-47.6)<br>23% bilateral	7.5 (5-13) years	7.3±1.7 (1-10)<br>80% UCLA ≥7<br>27% UCLA ≥8
Chang et al. 2014	N: 369<br>Age: 68.8 (50-83)<br>8% male<br>BMI: 27.4 (19.3-39.1)	2 (1-3) years	4.8±1.4
Crawford et al. 2020	N: 1611<br>Age: 63.9<br>34% male<br>BMI: 33.9	11.4 (5.1-15.9) years	4.7±1.70
Eckhard et al. 2020	N: 381<br>Age: 68.0±8.0<br>47% male<br>BMI: 31.2±6.3	1 year	5.9±1.7
Ghomrawi et al. 2017	N: 364, 69 had 2yr FU<br>Age: 67.1±9.4<br>33% male	2 years	5.9±2.1
Ho et al. 2016	N: 39<br>Age: 59 (57-64)<br>30% male<br>BMI: 32.5 [28.8-38.4]<br>3% bilateral	4.0±1.2 years	6.1±1.4
Jassim et al. 2019	N: 24<br>Age: 60±2.5<br>42% male	3±0.9 years	7.3±0.9 (6-10)

Arthroplasty/Measurement Method/Study	Age/BMI/gender	Follow-up time	Outcome
Keeney et al. 2014	N: 181<br>Age 49.0<br>26% male<br>BMI: 34.4<br>21% bilateral	36 (12-118) months	4.6<br>56% UCLA <5
	N: 262<br>Age: 69.9<br>40% male<br>BMI: 31.5<br>20% bilateral	31 (12-110) months	4.9<br>52% UCLA <5
Meding et al. 2012	N: 62<br>Age: 63.8±8.9<br>27% male<br>58% bilateral	21.1±1.6 years	8.3±1.2 (5-10)<br>35% UCLA ≥9
Pioger et al. 2020	N: 43 preoperatively active golfers<br>Age: 65.7±(43-85)<br>85.3% male<br>0% bilateral	4.6 (2.0-14.2) years	7.9±1.14
Plassard et al. 2020	N: 443<br>Age: 69 (41-90)<br>36.6% male<br>BMI: 29.3 (19-46)	43 [23-49] months	5.92<br>12% UCLA ≤3<br>55% UCLA 4-6<br>33% UCLA ≥ 7
Sandberg et al. 2019	N: 183<br>Age: 72.1±7.8<br>27.9% male<br>BMI: 33.1±6.4	12.9 months	5.1±1.68<br>6.2% UCLA >7
	N: 183 Dual pivot TKA<br>Age: 67.8±7.9<br>27.2% male<br>BMI: 33.4±6.5	12.6 months	5.3±1.85<br>12.2% UCLA >7
Scott et al. 2017	N: 289<br>Age: 59 (42-65)<br>49% male<br>BMI: 31.4 (15-55)<br>8% bilateral	3.3 (2-4) years	6<br>46% UCLA ≥7
Stambourgh et al. 2014	N: 76<br>Age: 48.7 (26-55)<br>30% male<br>BMI: 33.9 (14.9-54.9)<br>7% bilateral	4.8 (2-13.9) years	4.7 (2-10)
Tegner			
Hepperger et al. 2018	N: 200<br>Age: 72±7.7<br>40% male<br>BMI: 27.2±5.0<br>17.5% bilateral	1 & 2 years	1yr: 3.0 [3.0, 4.0]<br>2yr: 3.0 [3.0, 4.0]
Long et al. 2014	N: 36<br>Age: 51 (22-55)<br>25% bilateral	8 (3-18) years;<br>25.1 (20-35) years	8yr: 3.5±1.1<br>25yr: 3.0±1.6
Vielgut et al. 2016	N: 236<br>Age: 62.7±11.4<br>18% male<br>10% bilateral	14.9±3.0 years	3.04±1.5
Other			
Dubin et al. 2020	N: 121,LEAS <10 preop<br>Age: 61.7<br>43% male<br>BMI: 33.2	2.0 years	LEAS:<br>8.7
	N: 127, LEAS ≥10 preop<br>Age: 61.7<br>49% male<br>BMI: 32.2	2.0 years	LEAS:<br>10.8

Arthroplasty/Measurement Method/Study	Age/BMI/gender	Follow-up time	Outcome
Ghomrawi et al. 2017	N: 364, 69 had 2yr FU<br>Age: 67.1±9.4<br>33% male	2 years	LEAS:<br>10.9±3.0
Issa et al. 2015	N: 281<br>Age: 66 (39-80)<br>38% male<br>2% bilateral	1, 2, 3, 4, 5 years	LEAS:<br>1yr: 11.19, 2yr: 11.49, 3yr: 11.19, 4yr: 11.11, 5yr: 11.47
Ponzio et al. 2018	N: 1008 active group preop (LEAS >12) (N: 772 at FU)<br>Age: 66.3±9.1<br>56.6%<br>BMI: 28.3±5.0	2 years	LEAS:<br>13.7±2.7
	N: 1008 inactive group preop (LEAS 7-12) (N=727 at FU)<br>Age: 66.3±9.0<br>56.6% male<br>BMI: 28.4±4.9	2 years	LEAS:<br>11.6±2.9
**Unicompartimental knee arthroplasty**			
UCLA			
Canetti et al. 2018	N: 11, UCLA ≥5<br>Age: 66.5±6.8<br>BMI: 24.2±4.3<br>18% male<br>Surgery: Robotic UKA	34.4±10.5 months	6.4±1.6
	N: 17, UCLA ≥5<br>Age: 59.5±9.9<br>BMI: 26.3±3.8<br>28% male<br>Surgery: normal UKA	39.3±15.5 months	5.8±0.9
Felts et al. 2010	N: 62<br>Age: 54.7±5<br>47% male<br>BMI: 28±4<br>5% bilateral	11.2±5 years	6.4±2.3<br>40% UCLA ≥8
Ho et al. 2016	N: 33<br>Age: 60 (53-64)<br>33% male<br>BMI: 30.3 [27.6-33.7]<br>9% bilateral	4.0±1.2 years	7.4±1.6
Jacquet et al. 2020	N: 50 participating in high-impact sport preop<br>Age: 50.8±4.4	3.9±1.8 (2-5.7) years	6.5±2<br>28% UCLA >8
Jahnke et al. 2015	N: 135<br>Age: 67.1 (38-88)<br>53.4% male<br>9% bilateral	2±1.47 years	6.3±1.08
Kim et al. 2019	N: 42<br>Age: 63.6±5.5<br>17% male<br>BMI: 25.3±2.4<br>0% bilateral	24 months	5.1
Kleeblad et al. 2020	N: 164<br>Age: 62.3±8.8<br>55% male<br>BMI: 27.6±4.4<br>9% bilateral	20.2 months	6.8±1.9
Panzram et al. 2018	N: 27<br>Age: 62.5±8.3 (49-76)<br>56% male<br>11% bilateral	60±8.3 (47-69) months	6.1±1.8<br>11% UCLA <4<br>51% UCLA 4-6<br>37% UCLA >6

Arthroplasty/Measurement Method/Study	Age/BMI/gender	Follow-up time	Outcome
Panzram et al. 2020	N: 177<br>Age at FU: 64.4±9.7 (38-82)<br>BMI at FU: 30.9±5.4<br>8% bilateral	37.1±9.8 (24-60) months	Post: 6.3±1.4 (2-10)<br>53.7% UCLA ≥7
Pietschmann et al. 2013	N: 131<br>Age: 65.3 (44-83)<br>44% male<br>6% bilateral	4.2 (1-10) years	6
Walker et al. 2015a	N: 45<br>Age: 60.1±10.5<br>42% male<br>BMI: 27<br>0% bilateral	3 (2.0-4.3) years	6.7±1.5<br>66% UCLA ≥7
Walker et al. 2015b	N: 93<br>Age: 55 (36-60)<br>48% male<br>BMI: 32 (20-58)<br>17% bilateral	4.4±1.6 (2.3-8.4) years	6.8±1.5<br>62% UCLA ≥7
Zimmerer et al. 2021	N: 19<br>Age: 26.7±10.5 (36.0-76.0)<br>26% male<br>BMI: 27.2±5.7 (17.3-38.0)	4.6±2.2 (2.0-9.7) years	6.4±1.3 (4-9)<br>47% UCLA ≥7
Tegner			
Jahnke et al. 2015	N: 135<br>Age: 67.1 (38-88)<br>53.4% male<br>9% bilateral	2±1.47 years	3.96±0.96
Kim et al. 2019	N: 42<br>Age: 63.6±5.5<br>17% male<br>BMI: 25.3±2.4<br>0% bilateral	24 months	3.6
Krych et al. 2017	N: 183 at baseline<br>Age: 49.2<br>55% male<br>BMI: 32.4	1, 2, 5, 5.8 years	1yr (n=76): 4.07<br>2yr (n=133): 4.33<br>5yr (n=67): 4.48<br>5.8yr (n=48): 4.48
Panzram et al. 2018	N: 27<br>Age: 62.5±8.3 (49-76)<br>56% male<br>11% bilateral	60±8.3 (47-69) months	3.4±1.0
Panzram et al. 2020	N: 177<br>Age at FU: 64.4±9.7 (38-82)<br>BMI at FU: 30.9±5.4<br>8% bilateral	37.1±9.8 (24-60) months	3.5±1.0 (0-7)
Walkner et al. 2015a	N: 45<br>Age: 60.1±10.5<br>42% male<br>BMI: 27<br>0% bilateral	3 (2.0-4.3) years	3.5±0.8
Walker et al. 2015b	N: 93<br>Age: 55 (36-60)<br>48% male<br>BMI: 32 (20-58)<br>17% bilateral	4.4±1.6 (2.3-8.4) years	3.8±1.1
**Mixed arthroplasty**			
UCLA			
Abe et al. 2014	N: 608<br>Age: 62 (36-98)<br>14% male<br>BMI: 23 (14-34) kg/m^2<br>32% bilateral<br>90% THA, 10% HRA	4.8 (2.3-7.8) years	Joggers: 10±0;<br>Non-joggers: 6.6±2.4

Arthroplasty/Measurement Method/Study	Age/BMI/gender	Follow-up time	Outcome
Karampinas et al. 2017	N: 48<br>Age: 65 (52-70)<br>60% male<br>15% bilateral	2 years	Short metaphyseal hip arthroplasty (n=18): 7.9<br>Hip-resurfacing arthroplasty (n=15): 8.1<br>Big femoral head arthroplasty (n=16): 6.7
Lubbeke et al. 2014 (THA 97%, HRA 3%)	N: 1085<br>Age: 67.1±11.7<br>44.6% male<br>BMI: 27.0±4.6	5 years	5.7<br>27.9% UCLA ≥7
	N: 757<br>Age: 65.1±11.0<br>45.8% men<br>BMI: 26.7±4.2	10 years	5.5<br>28.2% UCLA ≥7
Schneider et al. 2020 (TKA 50%, medial UKA 32%, lateral UKA19%)	N: 92 did sports preoperatively<br>Age: 56±9.1<br>13% male<br>BMI: 28±5.6	1-2 years	TKA: 6.62±1.9<br>60.8% UCLA ≥7<br>UKA: 6.89±2.0<br>54.3% UCLA ≥7
Other			
Plate et al. 2013	N: 2 matched groups of 30<br>Age: 53 (37-79)<br>37% male	HRA: 38 (24-66) months<br>MoP THA: 29 (24-60) months	Weighted activity score (0-9 low, >9 high):<br>HRA: 10.5 (1-28)<br>MoP THA: 5.6 (1-18)
Smith et al. 2017b (THA/TKA)	N: 201<br>Age: 72.9±10.7<br>38% male<br>0% unilateral	2-4 years	Face-to-face interviews, summary index:<br>Sedentary (mild exercise 1-3x/month, no MPA or VPA): 12.9% (9-18)<br>Mildly active (≥1x/week MPA): 20.4% (15-27)<br>Moderately active (>1x/week moderately active or 1-4x/ month VPA): 54.7% (48-61)<br>Highly active (>1x/week vigorous work or VPA): 11.9% (8-17)

*At time of surgery, unless indicated otherwise.

BMI: body mass index; CoC: ceramic-on-ceramic; FU: follow-up; HRA: hip resurfacing arthroplasty ; LEAS: Lower Extremity Activity Scale; MoP: metal-on-polyethylene; MPA: moderate physical activity; PA: physical activity; THA: total hip arthroplasty; TKA: total knee arthroplasty; UCLA: University of California Los Angeles activity scale; UKA: unicompartimental knee arthroplasty; VPA: vigorous physical activity

Devane activity score (range 1-5) 1 strenuous labour/contact sports - 5 sedentary dependent; Grimby scale (range 1-4) 1 sedentary – 4 regular hard physical training for competition sports (vigorous physical activity; LEAS: Lower Extremity Activity Scale (range 1-18) 1 I am confined to my bed all day – 18 I am up and about at will in my house and outside. I also participate in vigorous physical activity such as competitive level sports daily; Sports activity index (range 0-25) 0-6 = non-active; 7-10 = moderately active; 11-25 = active; Tegner activity scale (range 0-10) 0 is on sick leave/disability - 10 is participation in competive sports at a national or international elite level; UCLA: University of California Los Angeles activity scale (range 1-10) 1 wholly inactive: dependent on others; cannot leave residence – 10 regular participation in impact sports such as tennis, acrobatics, ballet, heavy labour or backpacking; Weighted activity score (0-9 low, >9 high)
