# Supplementary material for: Amount and type of physical activity and sports from one year forward after hip or knee arthroplasty—A systematic review
Source: PLoS One. 2021 Dec 28;16(12):e0261784. doi: 10.1371/journal.pone.0261784 (PMC8714096; doi:10.1371/journal.pone.0261784)
Supplement: S7 Appendix — (PDF) [file pone.0261784.s007.pdf]

Appendix 7. Sports activity categorized into levels of sports participation, time spent playing sports (including session length and frequency), and impact and intensity.

	Characteristics*	Time after surgery	Participation rate	Time spent playing sports	Frequency	Session length	Intensity level
**Total hip arthroplasty**							
General sports							
Bonnin et al. 2018	N: 1042<br>Age: 60.6 ± 8.8<br>57% male<br>BMI: 26.6 ± 4.6	44.4 ± 14.0 months	91% participated regularly or frequently in sports				2.6% competitive
Bonnin et al. 2020	N: 259, posterolateral approach<br>Age: 61 ± 7.8 (39-78)<br>63% male<br>BMI: 27 ± 4.2 (17-41)	44 ± 14.1 (24-74) months	81% participated regularly or frequently in sports				
	N: 259, anterolateral approach<br>Age: 62±8.1 (21-76)<br>59% male<br>BMI: 27±4.6 (18-45)	43±14.5 (24-79) months	81% participated regularly or frequently in sports				
Breuer et al. 2020	N: 55<br>Age 61±10<br>53% male<br>BMI: 27.9±4.9	38±4.6 months	83%†				
Donner et al. 2019	N: 51<br>Age: 63.1 (36.7-76.8)<br>56.9% male<br>BMI (median): 27.6 (16.6-41.8)<br>100% bilateral	4.9 (4.3-5.8) years	76.5% participated regularly in sports	4.2 hours/wk	3.4±2.9 sessions/wk		
Hara et al. 2018	N: 524<br>Age: 62.9±10.1<br>16% male<br>BMI: 22.9±3.3<br>19% bilateral	68.2 months	30.5% participated at least once a month in sports				

	Characteristics*	Time after surgery	Participation rate	Time spent playing sports	Frequency	Session length	Intensity level
Heiberg et al. 2016	N: 30<br>Age: 70.5±8.4<br>56.7% male	5 years			3±1.0 sessions/wk		
Innmann et al. 2016	N: 86<br>Age: 52 (21-60)<br>61% male<br>BMI: 27 (18-39)<br>4% bilateral	11 (10-12) years	79%†		2.6 sessions/wk	55 min	
Jassim et al. 2019	N: 40<br>Age: 53.1±8.4<br>47% male	3.3±1.1 years			7.5% 1 session/wk<br>43% 2-3 sessions/wk<br>45% 4-6 sessions/wk<br>5% daily		
Madrid et al. 2019	N: 535<br>Age: 67 (13 -91)<br>31% male<br>BMI: 25.5±3.9	>1 year	7.1%† participated in institutionalized sports	42% <5 hrs/wk<br>37% 5-10 hrs/wk<br>21% 10-20 hrs/wk<br>0% >20 hrs/wk			
Ollivier et al. 2014	N: 571<br>Age: 61.3±10.9<br>Gender: 52% male<br>BMI: 27±3.2<br>0% bilateral	9.8±2.9 years	64%†				
Ortmaier et al. 2017	N: 137<br>Age: 65.6±12.4<br>BMI: 26.6±4	20.4±2.3 (18-22) months	87%†		18% 0 sessions/wk<br>12% 1 session/wk<br>20% 3 sessions/wk<br>9% 4 sessions/wk<br>27% >4 sessions/wk	24% 0-15min<br>9% 15-30 min<br>15% 30-60 min<br>22% 60-120 min<br>29% >120 min	>80% recreational
Payo-Ollero et al. 2020	N: 46<br>Age: 41 (37-48)<br>72% male<br>BMI: 26.1 (24.5-29)<br>25% bilateral	7.5 (1-11) years	78%†				
Schmidutz et al. 2012	N: 68<br>Age: 55±12<br>Gender: 60% male<br>BMI: 26±4 kg/m2<br>12% bilateral<br>Short-stem hip arthroplasty	2.7±0.7 (2.0-4.2) years	91%†		1.8±1.1 sessions/wk	66±33 min	

	Characteristics*	Time after surgery	Participation rate	Time spent playing sports	Frequency	Session length	Intensity level
Smith et al. 2018	N: 105<br>Age: 68.2±9.3<br>42.9% male<br>BMI: 28.8±4.2	12 & 24 months		22.2% <1 hr/wk<br>0% 1-2 hrs/wk<br>66.7% 2-4 hrs/wk<br>11.1% >4 hrs/wk	87.5% never<br>9.7% 1/2 sessions/wk<br>2.8% 3/4 sessions/wk<br>0% 5-7 sessions/wk		Light intensity
				11.1% <1 hr/wk<br>11.1% 1-2 hrs/wk<br>33.3% 2-4 hrs/wk<br>45.4% >4 hrs/wk	94.4% never<br>2.8% 1/2 sessions/wk<br>1.4% 3/4 sessions/wk<br>1.4% 5-7 sessions/wk		Moderate intensity
				36.8% <1 hr/wk<br>47.4% 1-2 hrs/wk<br>15.8% 2-4 hrs/wk<br>0% >4 hrs/wk	73.6% never<br>4.2% 1/2 sessions/wk<br>13.9% 3/4 sessions/wk<br>8.3% 5-7 sessions/wk		Strenuous intensity
Judo							
Lefevre et al. 2013	N: 27 judokas<br>Age: 63±7.2<br>33% bilateral	8.8±7.1 years	81.5%†		2.5±1 sessions/wk		0% competitive
**Hip resurfacing**							
General sports							
Amstutz et al. 2019	N: 661<br>Age: 51.9 (14-78)<br>70% male<br>BMI: 26.5 (16.7-46.5)	10.1 (1-16) years		Activity quantity (frequency†duration):<br>Frequency:<br>1: 1-4x/month;<br>2: 5-8x/month;<br>3: 9-12x/month;<br>4: >12x/month;<br>Duration<br>1: 0-30min;<br>2: 30-60min;<br>3: 60-120min;<br>4: >120min<br>12.4			
Banerjee et al. 2010	N: 138<br>Age: 52.6 (38-71)<br>Male hips: 59%<br>10% bilateral	23.5 (12-42) months	97%†,<br>61% daily				
Fisher et al. 2011	N: 117<br>Age: 54 (30-73)<br>57% male<br>14% bilateral	30 (16-50) months	73% participated in sports at least once a month				

	Characteristics*	Time after surgery	Participation rate	Time spent playing sports	Frequency	Session length	Intensity level
Girard et al. 2013	N: 50 participating in high-impact sport preop<br>Age: 51.5 (30.8-64.8)<br>90% male<br>BMI: 23.7 (21.7-33.6)<br>10% bilateral	44.1 (39.1-54.5) months	98%†	2.9 (2-9) hrs/wk			
Le Duff & Armstutz, 2011	N: 201<br>Age: 49.6<br>74.6% male<br>BMI: 26.9 (19-46)<br>28% bilateral	1.8 (1.0-4.9) years,<br>9.1 (5.0-13.4) years	1.8yr: 96%<br>9.1yr: 90% in at least one sporting activity		1.8yr: 33.9% >12 sessions/month<br>9.1yr: 33.1% >12 sessions/month	1.8yr: 45% sessions 30-60 min;<br>9.1yr: 36% sessions 30-60 min	1.8yr: 5.5% competitive<br>9.1yr: 10.1% competitive
Sandiford et al. 2015	N: 79 active patients<br>Age: 54.9 (3405-73.6)<br>67% male<br>BMI: 25.2 (19.8-31.9)<br>1% bilateral	8.5 (8-10) years	97%†		100% >3 sessions/wk	100% 60-90 min	14% competitive
Jogging							
Fouilleron et al. 2012	N: 40 runners preop<br>Age: 50.7 (31-61)<br>90% male<br>BMI: 24.8 (21.7-33.6)<br>8% bilateral	33.3 (26-41) months	91.6%†	3.1 hrs/wk			s: 28.4 km/wk
**Total knee arthroplasty**							
General sports							
Hepperger et al. 2018	N: 200<br>Age: 72±7.7<br>40% male<br>BMI: 27.2±5.0<br>17.5% bilateral	1 year<br><br><br><br>2 years	82% at least occasionally, 69% at least2x/week<br><br>83% at least occasionally,<br>70% at least 2x/week		18% no sports<br>13% occasionally<br>59% 2-3 sessions/wk<br>10% >5 sessions/wk<br>17% no sports<br>13% occasionally<br>57% 2-3 sessions/wk<br>13% >5 sessions/wk		
Ho et al. 2016	N: 39<br>Age: 59 (57-64)<br>30% male<br>BMI: 32.5 [28.8-38.4]<br>3% bilateral	4.0±1.2 years	70%†				

	Characteristics*	Time after surgery	Participation rate	Time spent playing sports	Frequency	Session length	Intensity level
Jassim et al. 2019	N: 24<br>Age: 60±2.5<br>42% male	3±0.9 years			17% once a week<br>25% 2-3 sessions/wk<br>46% 4-6 sessions/wk<br>13% daily		
Mayr et al. 2015	N: 81<br>Age at FU: 71.8±5.4<br>47% male<br>BMI: 28.4	6.4±0.9 years		5.3 hrs/wk	3.5 sessions/wk		
Plassard et al. 2020	N: 443<br>Age: 69 (41-90)<br>36.6% male<br>BMI: 29.3 (19-46)	43 [23-49] months	85%†				
Ristolainen et al. 2019	N: 70, knee injury as cause of TKA<br>Age: 51.8±5.2<br>BMI: 29.0±5.0	6.2±3.7	All: 78%<br>Sports injury group: 89%<br>Non-sports injury group: 74%<br>Regular exercise		>50% exercised >10x/month	>1 hour for 50% of patients	Total: 68% walking intensity, 14% high-intensity activity
Smith et al. 2018	N: 116<br>Age: 67.3±8.3<br>41.4% male<br>BMI: 30.1±4.9	12 & 24 months		0% <1 hr/wk<br>12.5% 1-2 hrs/wk<br>56.3% 2-4 hrs/wk<br>31.2% >4 hrs/wk	84.5% never<br>13.6% 1/2 sessions/wk<br>4.9% 3/4 sessions/wk<br>0% 5-7 sessions/wk		Light
				38.5% <1 hr/wk<br>15.4% 1-2 hrs/wk<br>46.1% 2-4 hrs/wk<br>0% >4 hrs/wk	87.4% never<br>6.8% 1/2 sessions/wk<br>4.9% 3/4 sessions/wk<br>0.9% 5-7 sessions/wk		Moderate
				51.4% <1 hr/wk<br>34.3% 1-2 hrs/wk<br>14.3% 2-4 hrs/wk<br>0% >4 hrs/wk	66.0% never<br>8.7% 1/2 sessions/wk<br>18.4% 3/4 sessions/wk<br>6.9% 5-7 sessions/wk		Strenuous
Vielgut et al. 2016	N: 236<br>Age: 62.7±11.4<br>18% male<br>10% bilateral	14.9±3.0 years	70.8% Participated regularly in sports				
Jogging							
Ho et al. 2016	N: 39<br>Age: 59 (57-64)<br>30% male<br>BMI: 32.5 [28.8-38.4]<br>3% bilateral	4.0±1.2 years	2% (n=1)		15 sessions/wk	60 min	

	Characteristics*	Time after surgery	Participation rate	Time spent playing sports	Frequency	Session length	Intensity level
Tennis							
Ho et al. 2016	N: 39<br>Age: 59 (57-64)<br>30% male<br>BMI: 32.5 [28.8-38.4]<br>3% bilateral	4.0±1.2 years	2% (n=1)				
Other							
Ho et al. 2016	N: 39<br>Age: 59 (57-64)<br>30% male<br>BMI: 32.5 [28.8-38.4]<br>3% bilateral	4.0±1.2 years	Golf: 18%<br>Bowling: 0%<br>Cycling: 38%<br>Swimming: 22%<br>Hiking: 12%<br>Dancing: 28%<br>Basketball: 0%<br>Other: 18%		1 [1-3] sessions/wk<br>12 [4-15] sessions/wk<br>4 [3.5-13] sessions/wk<br>4 [1.2-12] sessions/wk<br>1.5 [0.9-4.5] sessions/wk<br>12 [8-30]	60 [60-120] min<br>30 [20-40] min<br>30 [30-45] min<br>30 [20-60] min<br>45 [15-60] min<br>90 [55-180] min	
Lefevre et al. 2013	N: 8 judokas<br>Age: 72.8±5.2<br>25% bilateral	7±5.9 years	62.5%†		2.5±1 sessions/wk		0% competitive
Pioger et al. 2020	N: 143 active golfers<br>Age: 65.7±(43-85)<br>85.3% males<br>Bilateral: 0%	4.6 (2.0-14.2) years		10.2±6.6 hrs/wk			Handicap: 24.4±13.3
**Unicompartimental knee arthroplasty**							
General sports							
Canetti et al. 2018	N: 11, UCLA ≥5 preop<br>Age: 66.5±6.8<br>BMI: 24.2±4.3<br>18% male<br>Surgery: Robotic UKA	34.4±10.5 months	100%†				
	N: 17, UCLA ≥5 preop<br>Age: 59.5±9.9<br>BMI: 26.3±3.8<br>28% male<br>Surgery: normal UKA	39.3±15.5 months	94%†				
Ho et al. 2016	N: 33<br>Age: 60 (53-64)<br>33% male<br>BMI: 30.3 [27.6-33.7]<br>9% bilateral	4.0±1.2 years	72%†				

	Characteristics*	Time after surgery	Participation rate	Time spent playing sports	Frequency	Session length	Intensity level
Jahnke et al. 2015	N: 135<br>Age: 67.1 (38-88)<br>53.4% male<br>9% bilateral	2±1.47 years	93.2%,<br>87% weekly				
Kim et al. 2019	N: 42<br>Age: 63.6±5.5<br>17% male<br>BMI: 25.3±2.4<br>0% bilateral	24 months	76.2%†		3.0±1.3 sessions/wk	1.3±0.7 hours/session	
Kleeblad et al. 2020	N: 164<br>Age: 62.3±8.8<br>55% male<br>BMI: 27.6±4.4<br>9% bilateral	20.2 months	89.6%†				
Lo Presti et al. 2019	N: 53, did ≥1 sport preop<br>Age: 59.7 (46-66)<br>28.3% male	48±6 (24-72) months	90%†		1.9 sessions/wk	43 min	100% recreational
Panzram et al. 2018	N: 27<br>Age: 62.5±8.3 (49-76)<br>56% male<br>11% bilateral	60±8.3 (47-69) months	96%†				
Panzram et al. 2020	N: 177<br>Age at FU: 64.4±9.7 (38-82)<br>BMI at FU: 30.9±5.4<br>8% bilateral	37.1±9.8 (24-60) months	83.4%†		2.8±1.4 sessions/wk<br>15.6% >4 sessions/wk ≥60 min (%)		
Pietschmann et al. 2013	N: 131<br>Age: 65.3 (44-83)<br>44% male<br>Bilateral: 6%	4.2 (1-10) years	60%†				
Walker et al. 2015a (return to sports)	N: 45<br>Age: 60.1±10.5<br>42% male<br>BMI: 27<br>0% bilateral	3 (2.0-4.3) years	95.6%†		3 sessions/wk	44% >1 hour/session	
Walker et al. 2015b (Sports, PA)	N: 93<br>Age: 55 (36-60)<br>48% male<br>BMI: 32 (20-58)<br>17% bilateral	4.4±1.6 (2.3-8.4) years	91%†		53% ≥3 sessions/wk	45% ≥1 hour/sessions	

	Characteristics*	Time after surgery	Participation rate	Time spent playing sports	Frequency	Session length	Intensity level
Zimmerer et al. 2021	N: 19<br>Age: 26.7±10.5 (36.0-76.0)<br>26% male<br>BMI: 27.2±5.7 (17.3-38.0)	4.6±2.2 (2.0-9.7) years	68%†		2.3 sessions/wk	45.9 min/session	
Jogging							
Ho et al. 2016	N: 33<br>Age: 60 (53-64)<br>33% male<br>BMI: 30.3 [27.6-33.7]<br>9% bilateral	4.0±1.2 years	6% (n=2)		10 (8-12)	32 (20-45)	
Jahnke et al. 2015	N: 135<br>Age: 67.1 (38-88)<br>53.4% male<br>9% bilateral	2±1.47 years	24%		2.22<br>(0: 0 sessions/month<br>1: 1 sessions/month<br>2: 2 sessions/month<br>3: 1 session/wk<br>4: 2-3 sessions/wk<br>5: 2-3 sessions/wk<br>6: >4 sessions/wk)	2.16<br>(0= 0 min<br>1= <15 min<br>2= 15-60 min<br>3= 61-120 min<br>4= 121-180 min<br>5= >3 hrs)	
Tennis							
Ho et al. 2016	N: 33<br>Age: 60 (53-64)<br>33% male<br>BMI: 30.3 [27.6-33.7]<br>9% bilateral	4.0±1.2 years	0%				
Jahnke et al. 2015	N: 135<br>Age: 67.1 (38-88)<br>53.4% male<br>9% bilateral	2±1.47 years	6%		1.92<br>(0: 0 sessions/month<br>1: 1 session/month<br>2: 2 sessions/month<br>3: 1 session/wk<br>4: 2-3 sessions/wk<br>5: 2-3 sessions/wk<br>6: >4 sessions/wk)	1.62<br>(0 = 0 min<br>1= <15 min<br>2= 15-60 min<br>3= 61-120 min<br>4= 121-180 min<br>5= >3 hrs)	

	Characteristics*	Time after surgery	Participation rate	Time spent playing sports	Frequency	Session length	Intensity level
Other							
Ho et al. 2016	N: 33<br>Age: 60 (53-64)<br>33% male<br>BMI: 30.3 [27.6-33.7]<br>9% bilateral	4.0±1.2 years	Golf: 31%<br>Bowling: 8%<br>Cycling: 36%<br>Swimming: 14%<br>Hiking: 42%<br>Dancing: 6%<br>Basketball: 3% (n=1)<br>Other: 8%		4 [0.2-20] sessions/wk<br>3 (2-4) sessions/wk<br>8 [4-21] sessions/wk<br>4 [2.2-28.5] sessions/wk<br>4 [1-16] sessions/wk<br>6 [4-8] sessions/wk<br>1 session/wk<br>16 [12-16] sessions/wk	30 [30-60] min<br>100 (60-180) min<br>30[20-40] min<br>45 [17.5-60] min<br>60 [45-60] min<br>60 [60-60] min<br>60 min<br>45 [20-60] min	
Jahnke et al. 2015	N: 135<br>Age: 67.1 (38-88)<br>53.4% male<br>9% bilateral	2±1.47 years			Hiking: 3.02<br>Swimming: 2.37<br>Cycling: 3.34<br>CC skiing: 1.40<br>DH skiing: 0.58<br>Golf: 2.89<br>Dancing: 0.87<br>Tennis: 1.92<br>Ball games: 0.55<br>Other: 3.47<br>(0:0 sessions/month<br>1: 1 session/month<br>2: 2 sessions/month<br>3: 1 session/wk<br>4: 2-3 sessions/wk<br>5: 2-3 sessions/wk<br>6: >4 sessions/wk)	Hiking: 3.18<br>Swimming: 2.96<br>Cycling: 2.73<br>CC skiing: 2.20<br>DH skiing: 0.74<br>Golf: 4.00<br>Dancing: 1.13<br>Tannis: 1.62<br>Ball games: 0.55<br>Other: 2.88<br>(0 = 0 min<br>1= <15 min<br>2= 15-60 min<br>3= 61-120 min<br>4= 121-180 min<br>5= >3 hrs)	
**Mixed arthroplasty**							
General sports							
Naylor et al. 2019	N: 1289<br>Age: 67.2±9<br>45% men<br>BMI: 30.9±6<br>5% bilateral	3 years	THA: 67.3% ≥1 session/week<br>TKA: 60.6% ≥1 session/week				
Pisanu et al. 2020	N: 118<br>Age: 73 [67-77]<br>28.7% male<br>BMI: 29.1±4.7<br>3% bilateral	3.1±1.13 years	87%†				

	Characteristics*	Time after surgery	Participation rate	Time spent playing sports	Frequency	Session length	Intensity level
Jogging							
Abe et al. (2014)	Age: 63.8±11.7<br>55% male<br>BMI: 29.6±5.6	4.8 (2.3-7.8) years	3.8% participated in jogging at least 1x/week		4 (1-7) sessions/wk	29 (5-90) min	v: 7.7 (3-18) km/hour<br>s: 3.6 (0.5-15) km

*At time of surgery, unless indicated otherwise; † Definition not specified

Abbreviations: BMI: body mass index; FU: follow-up; HRA: hip resurfacing arthroplasty; THA: total hip arthroplasty; TKA: total knee arthroplasty; UKA: unicompartimental knee arthroplasty
