# Supplementary material for: Amount and type of physical activity and sports from one year forward after hip or knee arthroplasty—A systematic review
Source: PLoS One. 2021 Dec 28;16(12):e0261784. doi: 10.1371/journal.pone.0261784 (PMC8714096; doi:10.1371/journal.pone.0261784)
Supplement: S8 Appendix — (PDF) [file pone.0261784.s008.pdf]

Appendix 8. Participation rates per individual sport.

LOW-IMPACT

Arthroplasty/Study	Participants*	Low-impact total (%)	Cycling/ stationary cycling (%)	Fitness/ muscle training (%)	Golf (%)	Gymnastics (%)	Swimming (%)	Walking/ Nordic walking (%)	Aqua aerobics (%)	Other (%)
**Total hip arthroplasty**										
Batailler et al. 2019	N: 96<br>Age ~ 61<br>66% male<br>BMI: ~ 26<br>33% bilateral	82%								
Bonnin et al. 2018	N: 1042<br>Age: 60.6±8.8<br>57% male<br>BMI: 26.6±4.6<br>16% bilateral	51.1%	25.2% / 20.8%	41.5%	3.6%		19.4%			Fishing: 4.1%<br>Sailing: 1.0%<br>Yoga: 4.1%
Bonnin et al. 2020	N: 259, posterolateral approach<br>Age: 61±7.8 (39-78)<br>63% male<br>BMI: 27±4.2 (17-41)	49%	25% / 20%	15%	3%		17%			Fishing: 5%<br>Sailing: 1%<br>Yoga: 4%
	N: 259, anterolateral approach<br>Age: 62±8.1 (21-76)<br>59% male<br>BMI: 27±4.6 (18-45)	49%	24% / 22%	17%	3%		19%			Fishing: 4%<br>Sailing: 1%<br>Yoga: 4%
Donner et al. 2019	N: 51<br>Age: 63.1 (36.7-76.8)<br>56.9% male<br>BMI (median): 27.6 (16.6-41.8)		39%	33%	0%		26%		2%	Yoga: 2%
Hara et al. 2018	N: 524<br>Age: 62.9±10.1<br>16% male<br>BMI: 22.9±3.3<br>19% bilateral	4%		5%	3%	5%	12%	Walking: 16%		Bowling: 1%
Innmann et al. 2016	N: 86<br>Age: 52 (21-60)<br>61% male<br>BMI: 27 (18-39)<br>4% bilateral		42%	16%		17%	17%	Walking: 19%<br>Nordic walking: 7%	6%	

Arthroplasty/Study	Participants*	Low-impact total (%)	Cycling/ stationary cycling (%)	Fitness/ muscle training (%)	Golf (%)	Gymnastics (%)	Swimming (%)	Walking/ Nordic walking (%)	Aqua aerobics (%)	Other (%)
Madrid et al. 2019	N: 535<br>Age: 67 (13 -91)<br>31% male<br>BMI: 25.5±3.9		0%		3%		1%			Horseback riding: 0%
Ollivier et al. 2014	N: 571<br>Age: 61.3±10.9<br>Gender: 52% male<br>BMI: 27±3.2<br>0% bilateral		51.8% / 55.9%	52.7%	8.4%	72.4%	57.5%			Bowling: 23.1%<br>Stretching: 63.9%
Ortmaier et al. 2017	N: 137<br>Age: 65.6±12.4<br>BMI: 26.6±4		41% / 25%	12%	2%	19%	38%	Nordic walking: 30%	1%	Yoga: 1%<br>Rowing: 0%<br>Inline/ice skating: 0%<br>Horseback riding: 0%<br>Fishing: 2%
Payo-Ollero et al. 2020	N: 46<br>Age: 41 (37-48)<br>72% male<br>BMI: 26.1 (24.5-29)<br>25% bilateral		9% / 26%		4%		41%	28%		
Pritchett 2018	N: 160 needing unrestricted activity<br>Age: 43 (19-76)<br>48% male		3% (professional)			1% (teacher)	2%			Yoga: 7.%
Rolving et al. 2013	N: 95<br>Age: 72.3±6.2<br>33% male		48.4%		6.3%	19%		Walking: 79%		Swimming/aqua aerobics: 16.8%
Schmidutz et al. 2012	N: 68<br>Age: 55±12<br>60% male<br>BMI: 26±4<br>12% bilateral		69%	38%	1%	26%	56%	Nordic walking: 18%		Bowling: 6%<br>Riding: 3%
**Hip resurfacing arthroplasty**										
Amstutz et al. 2019	N: 661<br>Age: 51.9 (14-78)<br>70% male<br>BMI: 26.5 (16.7-46.5)	10.1 (1-16) years	32%	33%	14%		18%			Canoeing/ Kayaking/ Rowing: 1%<br>Flexibility/balance: 11%<br>Horseback riding: 1%
Banerjee et al. 2010	N: 138<br>Age: 52.6 (38-71)<br>Male hips: 59%		81% / 40.8%	25%	11.2%		57.9 %	Nordic walking: 20.4%	3.9%	

Arthroplasty/Study	Participants*	Low-impact total (%)	Cycling/ stationary cycling (%)	Fitness/ muscle training (%)	Golf (%)	Gymnastics (%)	Swimming (%)	Walking/ Nordic walking (%)	Aqua aerobics (%)	Other (%)
Fisher et al. 2011	N: 117<br>Age: 54 (30-73)<br>57% male<br>14% bilateral		16%		15%	5%	21%			Bowling: 4%
Sandiford et al. 2015	N: 79 active patients<br>Age: 54.9 (34.5-73.6)<br>67% male<br>BMI: 25.2 (19.8-31.9)<br>1% bilateral		13%		22%		23%	Walking: 15%		Yoga: 5%<br>Sailing: 4%<br>Horseback riding: 4%
**Total knee arthroplasty**										
Bonnin et al. 2010	N: 347<br>Age at FU: 74.8 (28-94)<br>35% male<br>BMI: 27.9±4.6	56%	13% / 24%	5%	2%	17%	22%			Sailing: 2%<br>Stretching: 17%
Chang et al. (2014)	N = 369<br>Age: 68.8 (50-83)<br>8% male<br>BMI: 27.4 (19.3-39.1)		21.7%		0.5%	4.6%	23.0%	Walking: 59.9%		Stretching: 5.5%<br>Gate ball: 1.1%
Hepperger et al. 2018	N: 200<br>Age: 72±7.7<br>40% male<br>BMI: 27.2±5.0<br>17.5% bilateral	Summer: 9%<br>Winter: 3%	55%				38%			
Ho et al. 2016	N: 39<br>Age: 59 (57-64)<br>30% male<br>BMI: 32.5 [28.8-38.4]<br>3% bilateral		38%		18%		22%			Bowling: 0%
Jones et al. 2012	N: 83<br>Age: 66±9.7<br>46% male<br>0% bilateral		24.1%	Stairmaster: 2.4%	3.6%		7.2%	Walkig: 63.9%	4.8%	Bowling: 2%<br>Fishing: 3.6<br>Canoeing/Rowing/ Kayaking; 1.2%<br>Yoga: 2.4%<br>Scuba diving: 0%
Mayr et al. 2015	N: 81<br>Age at FU: 71.8±5.4<br>47% male<br>BMI: 28.4	52%	1yr: 68%<br>5yr: 94%	1yr: 18%<br>5yr: 33%	1yr: 5%<br>5yr: 11%		1yr: 69%<br>5yr: 76%	Nordic walking:<br>1yr: 10%<br>5yr: 31%	1yr: 15%<br>5yr: 26%	

Arthroplasty/Study	Participants*	Low-impact total (%)	Cycling/ stationary cycling (%)	Fitness/ muscle training (%)	Golf (%)	Gymnastics (%)	Swimming (%)	Walking/ Nordic walking (%)	Aqua aerobics (%)	Other (%)
Plassard et al. 2020	N: 443<br>Age: 69 (41-90)<br>36.6% male<br>BMI: 29.3 (19-46)		27.0%	7.2%	2.0%		12.6%	59.3%		Petanque: 1.6%
Vielgut et al. 2016	N: 236<br>Age: 62.7±11.4<br>18% male<br>10% bilateral	71.3%	26.1%	3.3%	2.2%	21.7%	19.6%	Nordic walking: 21.7%		Bowling: 3.3%<br>Horseback riding: 1.1%
**Unicompartimental knee arthroplasty**										
Canetti et al. 2018	N: 28, UCLA ≥5 preop<br>Age: (35-79)<br>BMI: (20-36)<br>25% male		50%	Fitness/Yoga: 39%			14%			
Felts et al. 2010	N: 62<br>Age: 54.7±5<br>47% male<br>BMI: 28±4<br>5% bilateral		51%	14%	6%	3%	43%	Exercise walking: 35%		Horseback riding: 6%
Ho et al. 2016	N: 33<br>Age: 60 (53-64)<br>33% male<br>BMI: 30.3 [27.6-33.7]<br>9% bilateral		36%		31%		14%			Bowling: 8%
Jahnke et al. 2015	N: 135<br>Age: 67.1 (38-88)<br>53.4% male<br>9% bilateral		72%		6%		72%			
Kim et al. 2019	N: 42<br>Age: 63.6±5.5<br>17% male<br>BMI: 25.3±2.4<br>0% bilateral		7%	0%	7%		17%	Walking: 69%		Gymnastic riding: 24%
Kleeblad et al. 2020	N: 164<br>Age: 62.3±8.8<br>55% male<br>BMI: 27.6±4.4<br>9% bilateral	93.3%	45% / 27%				38%			Low-impact aerobics: 15%

Arthroplasty/Study	Participants*	Low-impact total (%)	Cycling/ stationary cycling (%)	Fitness/ muscle training (%)	Golf (%)	Gymnastics (%)	Swimming (%)	Walking/ Nordic walking (%)	Aqua aerobics (%)	Other (%)
Lo Presti et al. 2019	N: 53 practicing ≥1 sport preoperatively<br>Age: 59.7 (46-66)<br>28.3% male		26%				28%			
Panzram et al. 2018	N: 27<br>Age: 62.5±8.3 (49-76)<br>56% male<br>11% bilateral		63%	19%	11%	15%	15%	Walking: 33%<br>Nordic walking:	15%	
Panzram et al. 2020	N: 177<br>Age at FU: 64.4±9.7 (38-82)<br>BMI at FU: 30.9±5.4<br>8% bilateral		49%	21%	2%	7%	8%	Extended walks: 35%<br>Nordic Walking: 7%	8%	
Pietschmann et al. 2013	N: 131<br>Age: 65.3 (44-83)<br>44% male<br>6% bilateral		34%	8%	2%	9%	10%	Nordic walking: 8%		
Walker et al. 2015a	N: 45<br>Age: 60.1±10.5<br>42% male<br>BMI: 27<br>0% bilateral		73%	38%			33%	Walking: 44%<br>Nordic walking: 24%	18%	
Walker et al. 2015b	N: 93<br>Age: 55 (36-60)<br>48% male<br>BMI: 32 (20-58)<br>17% bilateral		68%	22%			48%	Walking: 52%<br>Nordic walking: 19%	22%	
**Mixed arthroplasty**										
Cowie et al. (2013)	N: 239<br>Age: 55.2±7.2<br>35.2% male<br>BMI: 28.2±4.9		12%		15%	15%	19%	Light walking: 16%<br>Exercise walking: 64%		Archery: 1%<br>Bowling: 3%<br>Yoga: 2%
Jassim et al. 2019	N: 64<br>Age: 55.7±7.5<br>45% male		6%		17%	14%	2%	Walking: 9%		Yoga: 3%<br>Scuba diving: 2%
Karampinas et al. 2017	N: 48<br>Age: 65 (52-70)<br>60% male<br>15% bilateral	52%								

Arthroplasty/Study	Participants*	Low-impact total (%)	Cycling/ stationary cycling (%)	Fitness/ muscle training (%)	Golf (%)	Gymnastics (%)	Swimming (%)	Walking/ Nordic walking (%)	Aqua aerobics (%)	Other (%)
Naylor et al. 2019	N: 1289<br>Age: 67.2±9<br>45% men<br>BMI: 30.9±6<br>5% bilateral		7%		6%	10%		Walking: 47%		Swimming/<br>Water aerobics: 9%<br>Yoga: 1%<br>Lawn bowls: 4%
Pisanu et al. 2020	N: 118<br>Age: 73 [67-77]<br>28.7% male<br>BMI: 29.1±4.7<br>3% bilateral		3% / 1%				3%	Walking: 74%	3%	Bowling: 4%

*At time of surgery, unless indicated otherwise.
Abbreviations: BMI: body mass index; FU: follow-up; UCLA: University of California Los Angeles activity scale

MEDIUM-IMPACT

Arthroplasty/Study	Participants	Medium-impact total (%)	Aerobics (%)	Dancing (%)	Hiking (%)	Rock/Mountain climbing (%)	Skiing (%)	Other* (%)
**Total hip arthroplasty**								
Bonnin et al. 2018	N: 1042<br>Age: 60.8±8.8<br>8% male<br>BMI: 26.6±4.6	72.7%		3.6%	41.5%	0.8%	CC: 4.3%<br>DH: 11.3%<br>Ski touring: 2.7%	Hunting: 3.7%<br>Tai-Chi-Chuan: 2.0%
Bonnin et al. 2020	N: 259, posterolateral approach<br>Age: 61±7.8 (39-78)<br>63% male<br>BMI: 27±4.2 (17-41)	69%		5%	41%	1%	CC: 4%<br>DH: 8%<br>Ski touring: 3%	Hunting: 5%<br>Tai-Chi-Chuan: 1%
	N: 259, anterolateral approach<br>Age: 62±8.1 (21-76)<br>59% male<br>BMI: 27±4.6 (18-45)	71%		6%	39%	1%	CC: 7%<br>DH: 15%<br>Ski touring: 5%	Hunting: 3%<br>Tai-Chi-Chuan: 1%
Donner et al. 2019	N: 51<br>Age: 63.1 (36.7-76.8)<br>56.9% male<br>BMI (median): 27.6 (16.6-41.8)			0%	19%		6%	
Hara et al. 2018	N: 524<br>Age: 62.9±10.1<br>16% male<br>BMI: 22.9±3.3<br>19% bilateral		1%	3%				
Innmann et al. 2016	N: 86<br>Age: 52 (21-60)<br>61% male<br>BMI: 27 (18-39)<br>4% bilateral				14%	1%	CC: 1%<br>DH: 1%	Table tennis: 1%
Madrid et al. 2019	N: 535<br>Age: 67 (13 -91)<br>31% male<br>BMI: 25.5±3.9				1%			Polo: 2.6%<br>Weightlifting: 2.6%
Ollivier et al. 2014	N: 571<br>Age: 61.3±10.9<br>Gender: 52% male<br>BMI: 27±3.2<br>0% bilateral			16.3%	52.7%		CC: 22.7%<br>DH: 36.4%	Hunting: 17.7%<br>Sailing: 22%

Arthroplasty/Study	Participants	Medium-impact total (%)	Aerobics (%)	Dancing (%)	Hiking (%)	Rock/Mountain climbing (%)	Skiing (%)	Other* (%)
Ortmaier et al. 2017	N: 137<br>Age: 65.6±12.4<br>BMI: 26.6±4		0%	4%	54%	0%	CC: 2%<br>DH: 14%<br>Ski tour: 2%	Waterskiing: 0%<br>Table tennis: 1%<br>Snowboarding: 0<br>Mountain biking: 1%
Payo-Ollero et al. 2020	Patients: 46<br>Age: 41 (37-48)<br>72% male<br>BMI: 26.1 (24.5-29)<br>25% bilateral		15%	4%	24%		4%	
Pritchett 2018	N: 160 needing unrestricted activity<br>Age: 43 (19-76)<br>48% male							Ballet: 3%<br>Pilates: 4%<br>Paddle board: 1%<br>Surfing: 5%
Rolving et al. 2013	N: 95<br>Age: 72.3±6.2<br>33% male			5.3%				
Schmidutz et al. 2012	N: 68<br>Age: 55±12<br>60% male<br>BMI: 26±4<br>12% bilateral			22%	57%	1%	CC: 15%<br>DH: 16%	Inline skating: 1%
**Hip-resurfacing arthroplasty**								
Amstutz et al. 2019	N: 661<br>Age: 51.9 (14-78)<br>70% male<br>BMI: 26.5 (16.7-46.7)		8%		Walking/ hiking: 54%		6%	Motor sports: 1%<br>Surfing: 2%
Banerjee et al. 2010	N: 138<br>Age: 52.6 (38-71)<br>Male hips: 59%		3%				CC: 7.9%<br>DH: 13.8%	Riding: 2.0%<br>Ice/inline skating: 5.3%<br>Snowboarding: 0%
Fisher et al. 2011	N: 117<br>Age: 54 (30-73)<br>57% male<br>14% bilateral			7%	13%			
Girard et al. 2013	N: 50 participating in high-impact sport pre-surgery<br>Age: 51.5 (30.8-64.8)<br>90% male<br>BMI: 23.7 (21.7-33.6)<br>10% bilateral							Surfing: 4%

Arthroplasty/Study	Participants	Medium-impact total (%)	Aerobics (%)	Dancing (%)	Hiking (%)	Rock/Mountain climbing (%)	Skiing (%)	Other* (%)
Sandiford et al. 2015	N: 79 active patients<br>Age: 54.9 (34.5-73.6)<br>67% male<br>BMI: 25.2 (19.8-31.9)<br>1% bilateral		29%	5%			4%	
**Total knee arthroplasty**								
Bonnin et al. 2010	N: 347<br>Age at FU: 74.8 (28-94)<br>35% male<br>BMI: 27.9±4.6	66%		3%	26%		CC: 11%<br>DH: 6%	
Chang et al. (2014)	N = 369<br>Age: 68.8 (50-83)<br>8% male<br>BMI: 27.4 (19.3-39.1)				6.0%			Table tennis: 0.8%
Hepperger et al. 2018	N: 200<br>Age: 72±7.7<br>40% male<br>BMI: 27.2±5.0<br>17.5% bilateral	Summer: 86%<br>Winter: 43%			74%		CC: 17%<br>DH: 70%	
Ho et al. 2016	N: 39<br>Age: 59 (57-64)<br>30% male<br>BMI: 32.5 [28.8-38.4]<br>3% bilateral			28%	12%		0%	
Jones et al. 2012	N: 83<br>Age: 66±9.7<br>46% male<br>0% bilateral		1.2%	7.2%	3.6%	0%	CC: 0%<br>DH: 0%	Tai Chi: 2.4%<br>Calisthenics: 32.5%<br>Hunting: 3.6%<br>Skating: 0%<br>Waterskiing: 0%
Mayr et al. 2015	N: 81<br>Age at FU: 71.8±5.4<br>47% male<br>BMI: 28.4	57%		1yr: 19%<br>6yr: 26%	1yr: 27%<br>6yr: 70%	1 yr: 3%<br>6 yr: 9%	CC: 1yr: 16%, 6yr: 27%<br>DH: 1yr: 17%, 6yr: 25%	
Plassard et al. 2020	N: 443<br>Age: 69 (41-90)<br>36.6% male<br>BMI: 29.3 (19-46)			0.5%	12.2%	0.3%	5.2%	Fishing/hunting: 1.2%

Arthroplasty/Study	Participants	Medium-impact total (%)	Aerobics (%)	Dancing (%)	Hiking (%)	Rock/Mountain climbing (%)	Skiing (%)	Other* (%)
Vielgut et al. 2016	N: 236<br>Age: 62.7±11.4<br>18% male<br>10% bilateral	43.7%		8.7%	26.4%	0%	CC: 0%<br>DH: 0%	Inline skating: 1.1%
**Unicompartimental knee arthroplasty**								
Canetti et al. 2018	N: 28, UCLA ≥5 preop<br>Age: (35-79)<br>BMI: (20-36)<br>25% male				82%		18%	
Felts et al. 2010	N: 62<br>Age: 54.7±5<br>47% male<br>BMI: 28±4<br>5% bilateral		5%	6%	57%	15%	CC: 8%<br>DH: 23%	
Ho et al. 2016	N: 33<br>Age: 60 (53-64)<br>33% male<br>BMI: 30.3 [27.6-33.7]<br>9% bilateral			6%	42%		0%	
Jahnke et al. 2015	N: 135<br>Age: 67.1 (38-88)<br>53.4% male<br>9% bilateral			20%	81%		CC: 4%<br>DH: 4%	
Kim et al. 2019	N: 42<br>Age: 63.6±5.5<br>17% male<br>BMI: 25.3±2.4<br>0% bilateral		0%	2%	29%	7%		
Kleeblad et al. 2020	N: 164<br>Age: 62.3±8.8<br>55% male<br>BMI: 27.6±4.4<br>9% bilateral	63.9%			24%			
Lo Presti et al. 2019	N: 53 patients doing ≥1 sport preop<br>Age: 59.7 (46-66)<br>28.3% male			15%		6%	CC: 2%<br>DH: 4%	
Panzram et al. 2018	N: 27<br>Age: 62.5±8.3 (49-76)<br>56% male<br>11% bilateral				22%	4%	CC: 0%<br>DH: 4%	

Arthroplasty/Study	Participants	Medium-impact total (%)	Aerobics (%)	Dancing (%)	Hiking (%)	Rock/Mountain climbing (%)	Skiing (%)	Other* (%)
Panzram et al. 2020	N: 177<br>Age at FU: 64.4±9.7 (38-82)<br>BMI at FU: 30.9±5.4<br>8% bilateral				23%	1% mountaineering	CC: 3%<br>DH: 2%	Inline skating: 1%
Pietschmann et al. 2013	N: 131<br>Age: 65.3 (44-83)<br>44% male<br>6% bilateral				10%	2%	CC: 2%<br>DH: 6%	Table tennis: 1%
Walker et al. 2015a	N: 45<br>Age: 60.1±10.5<br>42% male<br>BMI: 27<br>0% bilateral		20%		40%		CC: 2%<br>DH: 0%	
Walker et al. 2015b	N: 93<br>Age: 55 (36-60)<br>48% male<br>BMI: 32 (20-58)<br>17% bilateral		15%		35%		CC: 5%<br>DH: 5%	
**Mixed arthroplasty**								
Cowie et al. (2013)	N = 239<br>Age: 55.2±7.2<br>35.2% male<br>BMI: 28.2±4.9			3%	5%		0.1%	Horseback riding: 1%<br>Pilates: 3%<br>Weightlifting: 2%
Jassim et al. 2019	N: 64<br>Age: 55.7±7.5<br>45% male			5%				Pilates: 5%<br>Table tennis: 0%
Karampinas et al. 2017	N: 48<br>Age: 65 (52-70)<br>60% male<br>15% bilateral	38%						
Naylor et al. 2019	N: 1289<br>Age: 67.2±9<br>45% men<br>BMI: 30.9±6<br>5% bilateral							Tai-Chi: 1%<br>Pilates: 1%
Pisanu et al. 2020	N: 118<br>Age: 73 [67-77]<br>28.7% male<br>BMI: 29.1±4.7<br>3% bilateral				2%			Calisthenics: 2%<br>Weightlifting: 1%<br>Hunting: 3%

*At time of surgery, unless indicated otherwise. Abbreviations: BMI: body mass index; CC: Cross-country; DH: Downhill; FU: follow-up; UCLA: University of California Los Angeles activity scale

High-impact							
Arthroplasty/Author	Participants	High-impact total (%)	Contact sports (%)	Jogging (%)	Martial arts/Judo/Karate (%)	Racket sports (%)	Other
**Total hip arthroplasty**							
Bonnin et al. 2018	N: 1042<br>Age: 60.8±8.8<br>8% male<br>BMI: 26.6±4.6	19.9%	1.1%	4.4%	Judo/Karate: 0.7%	Tennis: 2.3%	Mountain run: 0.9%
Bonnin et al. 2020	N: 259, posterolateral approach<br>Age: 61±7.8 (39-78)<br>63% male<br>BMI: 27±4.2 (17-41)	19%	Team ball games: 2%	Running >500m: 7%<br>Trail/Mountain run: 2%	Judo/Karate: 1%	Tennis/Squash: 3%	
	N: 259, anterolateral approach<br>Age: 62±8.1 (21-76)<br>59% male<br>BMI: 27±4.6 (18-45)	24%	Team ball games: 1%	Running >500m: 4%<br>Trail/Mountain run: 1%	Judo/Karate: 0%	Tennis/Squash: 2%	
Donner et al. 2019	N: 51<br>Age: 63.1 (36.7-76.8)<br>56.9% male<br>BMI (median): 27.6 (16.6-41.8)	9.8%	Soccer: 2%<br>Basketball/<br>Handball: 2%	4%		Tennis: 4%	
Hara et al. 2018	N: 524<br>Age: 62.9±10.1<br>16% male<br>BMI: 22.9±3.3<br>19% bilateral	2.5%	Ball games: 2%	2%		Racket sports: 2%	
Innmann et al. 2016	N: 86<br>Age: 52 (21-60)<br>61% male<br>BMI: 27 (18-39)<br>4% bilateral		Soccer: 0%<br>Basketball: 0%<br>Handball: 0%	5%		Tennis: 6%	
Madrid et al. 2019	N: 535<br>Age: 67 (13 -91)<br>31% male<br>BMI: 25.5±3.9		Basketball: 0%<br>Soccer: 0%	0%	Martial arts: 0%	Tennis: 0.01%	
Ollivier et al. 2014	N: 571<br>Age: 61.3±10.9<br>Gender: 52% male<br>BMI: 27±3.2<br>0% bilateral		Football: 11.5%<br>Basketball/<br>Volleyball/<br>Handball: 16.7%	26.7%	Martial arts: 4.4%	Tennis/squash: 45%	

Arthroplasty/Author	Participants	High-impact total (%)	Contact sports (%)	Jogging (%)	Martial arts/Judo/Karate (%)	Racket sports (%)	Other
Ortmaier et al. 2017	N: 137<br>Age: 65.6±12.4<br>BMI: 26.6±4		Soccer: 0%<br>Handball/<br>Volleyball/<br>Basketball:<br>1%	2%		Tennis: 3%	Athletics: 0%
Payo-Ollero et al. 2020	N: 46<br>Age: 41 (37-48)<br>72% male<br>BMI: 26.1 (24.5-29)<br>25% bilateral		2%	4%		Singles tennis: 7%	High-impact aerobics: 4%
Pritchett 2018	N: 160 needing unrestricted activity<br>Age: 43 (19-76)<br>48% male				Martial arts: 6%		Bunjee jumping: 1%<br>Extreme skiing: 6%<br>Acrobatics: 1%<br>Triathlon: 5%<br>Rodeo: 2%
Rolving et al. 2013	N: 95<br>Age: 72.3±6.2<br>33% male			2.1%		Tennis/<br>badminton: 4.2%	
Schmidutz et al. 2012	N: 68<br>Age: 55±12<br>60% male<br>BMI: 26±4<br>12% bilateral		Handball: 0%<br>Basketball: 1%<br>Soccer: 1%	3%	Martial arts: 1%	Squash: 0%<br>Badminton: 3%<br>Tennis: 3%	Volleyball: 3%
**Hip-resurfacing arthroplasty**							
Amstutz et al. 2019	N: 661<br>Age: 51,9 (14-78)<br>70% male<br>BMI: 26,5 (16,7-46,5)	10.1 (1-16) years		4%	Martial arts: 3%	Racket sports: 7%	Team sports: 5%
Banerjee et al. 2010	N: 138<br>Age: 52.6 (38-71)<br>Male hips: 59%		Soccer: 3.9%<br>Basketball: 0.7%<br>Handball: 0%<br>Volleyball: 2.6%<br>Hockey: 0.6%	11.2%	Martial arts: 0%	Badminton: 3.9%<br>Squash: 0%<br>Tennis: 5.9%	
Fisher et al. 2011	N: 117<br>Age: 54 (30-73)<br>57% male<br>14% bilateral		Football: 2%	2%		Badminton: 1%<br>Squash: 2%<br>Tennis: 5%	

Arthroplasty/Author	Participants	High-impact total (%)	Contact sports (%)	Jogging (%)	Martial arts/Judo/Karate (%)	Racket sports (%)	Other
Girard et al. 2013	N: 50 did high-impact sports preop<br>Age: 51.5 (30.8-64.8)<br>90% male<br>BMI: 23.7 (21.7-33.6)<br>10% bilateral		Basketball: 4%<br>Soccer: 22%<br>Handball: 4%<br>Rugby: 2%	76%	Martial arts: 4%	Squash: 4%<br>Tennis: 8%	High-impact dance: 8%
Le Duff & Armstutz, 2011	N: 201<br>Age: 49.6<br>74.6% male<br>BMI: 26.9 (19-46)<br>28% bilateral	1.8y: 12.4%<br>9.1y: 17.5%					
Sandiford et al. 2015	N: 79 active<br>Age: 54.9 (34.5-73.6)<br>67% male<br>BMI: 25.2 (19.8-31.9)<br>1% bilateral			30%		Badminton: 3%<br>Squash: 1%<br>Tennis: 18%	
**Total knee arthroplasty**							
Bonnin et al. 2010	N: 347<br>Age at FU: 74.8 (28-94)<br>35% male<br>BMI: 27.9±4.6	10%		2%		Tennis: 1%	
Chang et al. (2014)	N = 369<br>Age: 68.8 (50-83)<br>8% male<br>BMI: 27.4 (19.3-39.1)			1.4%		Badminton: 1.6%	
Hepperger et al. 2018	N: 200<br>Age: 72±7.7<br>40% male<br>BMI: 27.2±5.0<br>17.5% bilateral	Summer: 5%<br>Winter: 54%					
Ho et al. 2016	N: 39<br>Age: 59 (57-64)<br>30% male<br>BMI: 32.5 [28.8-38.4]<br>3% bilateral		Basketball: 0%	2%		Tennis: 2%	
Jones et al. 2012	N: 83<br>Age: 66±9.7<br>46% male<br>0% bilateral		Basketball: 0%<br>Football: 0%<br>Soccer: 0%	0%	Martial arts: 0%	Racquetball: 0%<br>Squash: 0%<br>Tennis: 0%	Volleyball: 0%<br>Fencing: 0%<br>Jumping rope: 0%<br>Baseball/Softball: 0%

Arthroplasty/Author	Participants	High-impact total (%)	Contact sports (%)	Jogging (%)	Martial arts/Judo/Karate (%)	Racket sports (%)	Other
Mayr et al. 2015	N: 81<br>Age at FU: 71.8±5.4<br>47% male<br>BMI: 28.4	25%				Tennis: 1yr: 6%, 6yr: 20%	
Plassard et al. 2020	N: 443<br>Age: 69 (41-90)<br>36.6% male<br>BMI: 29.3 (19-46)			0.3%		Tennis: 0.7%	
Vielgut et al. 2016	N: 236<br>Age: 62.7±11.4<br>18% male<br>10% bilateral	16.7%	Handball: 1.1%<br>Basketball: 0%<br>Soccer: 2.2%	2.2%	Martial arts: 0%	Badminton: 3.3%<br>Squash: 1.1%<br>Tennis: 0%	Volleyball: 1.1%
**Unicompartimental knee arthroplasty**							
Canetti et al. 2018	N: 28, UCLA ≥5 preop<br>Age: (35-79)<br>BMI: (20-36)<br>25% male			0%			
Felts et al. 2010	N: 62<br>Age: 54.7±5<br>47% male<br>BMI: 28±4<br>5% bilateral		Soccer: 12%<br>Handball/<br>Volleyball/<br>Basketball: 8%	23%			
Ho et al. 2016	N: 33<br>Age: 60 (53-64)<br>33% male<br>BMI: 30.3 [27.6-33.7]<br>9% bilateral		Basketball: 3%	6%		Tennis: 0%	
Jahnke et al. 2015	N: 135<br>Age: 67.1 (38-88)<br>53.4% male<br>9% bilateral		Ball games: 1%	24%		Tennis: 6%	
Kim et al. 2019	N: 42<br>Age: 63.6±5.5<br>17% male<br>BMI: 25.3±2.4<br>0% bilateral		Soccer: 0%	2%		Tennis: 2%	

Arthroplasty/Author	Participants	High-impact total (%)	Contact sports (%)	Jogging (%)	Martial arts/Judo/Karate (%)	Racket sports (%)	Other
Kleeblad et al. 2020	N: 164<br>Age: 62.3±8.8<br>55% male<br>BMI: 27.6±4.4<br>9% bilateral	32.7%					
Lo Presti et al. 2019	N: 53 did ≥1 sport preop<br>Age: 59.7 (46-66)<br>28.3% male	21%	Football: 6%	9%		Tennis: 4%	
Panzram et al. 2018	N: 27<br>Age: 62.5±8.3 (49-76)<br>56% male<br>11% bilateral		Soccer: 0%	0%		Tennis: 7%	Volleyball: 4%
Panzram et al. 2020	N: 177<br>Age at FU: 64.4±9.7 (38-82)<br>BMI at FU: 30.9±5.4<br>8% bilateral		Handball/<br>Volleyball/<br>Basketball: 3%<br>Soccer: 2%	3%		Tennis: 5%	
Pietschmann et al. 2013	N: 131<br>Age: 65.3 (44-83)<br>44% male<br>6% bilateral		Soccer: 0%			Tennis: 0%	
Walker et al. 2015a	N: 45<br>Age: 60.1±10.5<br>42% male<br>BMI: 27<br>0% bilateral		Soccer: 0%	7%		Tennis: 0%	
Walker et al. 2015b	N: 93<br>Age: 55 (36-60)<br>48% male<br>BMI: 32 (20-58)<br>17% bilateral		Soccer: 6%	3%		Tennis: 4%	
**Mixed arthroplasty**							
Cowie et al. (2013)	N: 239<br>Age: 55.2±7.2<br>35.2% male<br>BMI: 28.2±4.9		Football: 1%			Badminton: 2%	
Jassim et al. 2019	N: 64<br>Age: 55.7±7.5<br>45% male		Football: 3%<br>Rugby: 0%	9%	Judo: 0%	Badminton: 2%<br>Tennis: 13%	Triathlon: 3%

Arthroplasty/Author	Participants	High-impact total (%)	Contact sports (%)	Jogging (%)	Martial arts/Judo/Karate (%)	Racket sports (%)	Other
Karampinas et al. 2017	N: 48<br>Age: 65 (52-70)<br>60% male<br>15% bilateral	13%					
Naylor et al. 2019	N: 1289<br>Age: 67.2±9<br>45% men<br>BMI: 30.9±6<br>5% bilateral			1%		Squash: 0%<br>Tennis: 1%	
Pisanu et al. 2020	N: 118<br>Age: 73 [67-77]<br>28.7% male<br>BMI: 29.1±4.7<br>3% bilateral		Soccer: 1%	1%			Motorcross: 1%

*At time of surgery, unless indicated otherwise.
Abbreviations: BMI: body mass index; FU: follow-up; UCLA: University of California Los Angeles activity scale
